# Mucosal‐Associated Invariant T Cells in Health and Disease

**DOI:** 10.1002/mco2.70996

**Published:** 2026-09-15

**Authors:** Yu Zhao, Feng‐Mi Ou, Dan Liang, Yang Gao, Qing‐Qing Li, Yi‐Qun Kuang

**Affiliations:** ^1^ Research Center for Clinical Medicine First Affiliated Hospital of Kunming Medical University Kunming China; ^2^ Yunnan Province Clinical Research Center For Laboratory Medicine Kunming China; ^3^ Yunnan Key Laboratory of Stem Cell and Regenerative Medicine Kunming Medical University Kunming China

**Keywords:** homeostasis maintenance, infectious and immune diseases, MAIT cells, MR1, therapeutic application

## Abstract

Mucosal‐associated invariant T (MAIT) cells are a population of evolutionarily conserved, unconventional innate‐like T lymphocytes that are restricted by major histocompatibility complex Class I‐related (MR1) molecules. They can be activated either in a T‐cell receptor–MR1‐ or cytokine‐dependent manner. Activated MAIT cells play crucial roles in maintaining homeostasis and the fine regulation of the immune system. This review presents a systematic exploration of the development, biological characteristics, and multiple effects of MAIT cells in health and various diseases. In healthy individuals, MAIT cells maintain mucosal barrier integrity, participate in tissue repair after injury, and prevent excessive inflammation. In microbial infections, MAIT cells eliminate infected cells and modulate immune responses or become pathogenic when overactivated by superantigens. In the context of immune‐related diseases, MAIT cells migrate to inflamed tissues and become pathogenic by secreting tissue‐damaging cytokines or protective by modulating immune responses. Moreover, MAIT cells have been demonstrated to have therapeutic applications in controlling diseases through their use as cellular adjuvants, their increased activation by engineered bacteria, and their modification with chimeric antigen receptors. This review synthesizes the functional plasticity of MAIT cells in health and disease, highlighting the therapeutic efficacy and limitations of engineered MAIT cells for precise intervention.

## Graphical abstract

The roles of MAIT cells in health and disease and their therapeutic applications. In healthy individuals, riboflavin metabolites secreted by commensal bacteria at mucosal sites are presented by MR1, leading to the activation of MAIT cells. Activated MAIT cells can maintain the barrier integrity of mucosal tissues in a steady state or migrate to the wound to perform tissue repair. In addition, activated MAIT cells can suppress excessive inflammation through secreting IL‐10. In infectious and immune‐related diseases, pathogens can synthesize and secrete riboflavin metabolites, which are presented by MR1 to activate MAIT cells in a TCR‐dependent manner. Whereas viruses, exogenous ligands, and cytokines stimulate immune cells to secrete IL‐12 and IL‐18, which can direct activate MAIT cells in a TCR‐independent manner. After activation, MAIT cells can exert protective effects by inhibiting viral replication and secreting cytotoxic factors that induce apoptosis or autophagy in infected cells. Additionally, activated MAIT cells can also contribute to pathogenesis by secreting IL‐17, IL‐23, and TNF‐α, thereby increasing inflammation and pathogenesis. MAIT cells can also promote angiogenesis and cell proliferation in tumor diseases. Moreover, the use of MAIT cells has led to the development of several therapeutic strategies. Activation of MAIT cells can be achieved either by stimulation with cytokines IL‐12, IL‐18, and IFN‐I produced following vaccine immunization, or by recognition of riboflavin metabolites secreted by engineered bacteria and then presented by MR1. Activated MAIT cells can also trigger the activation and expansion of other immune cells (e.g. CD8^+^T cells and B cells) and secrete IFN‐γ, GzmB, and TNF to combat infection. Additionally, CAR‐modified MAIT cells represent a promising strategy for killing tumor or pathogen‐infected cells and for controlling autoimmune diseases.

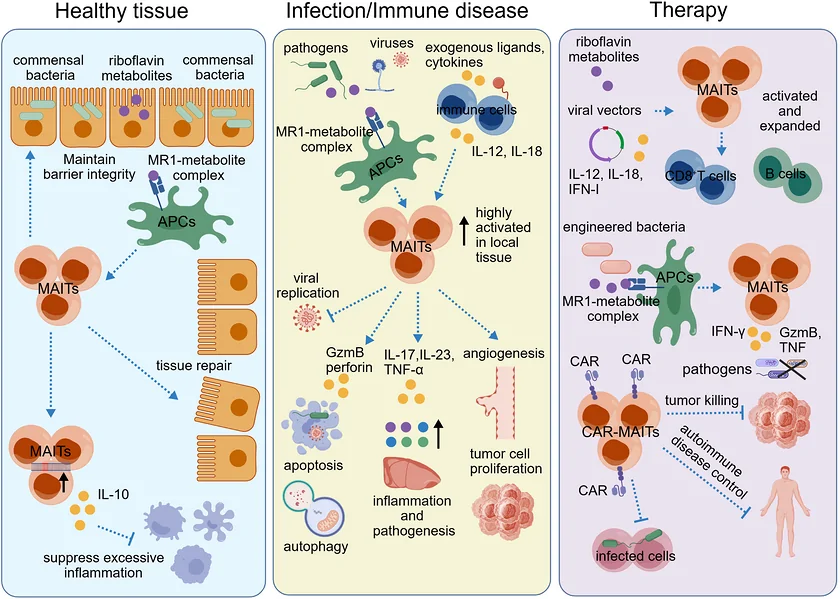

## 1 Introduction

Mucosal‐associated invariant T (MAIT) cells are a class of unconventional innate‐like T lymphocytes that are highly enriched in mucosal sites of the intestine, skin, respiratory, oral, and female genital tracts [1, 2]. Unlike conventional T cells, which recognize specific antigenic peptides presented by the major histocompatibility complex (MHC), MAIT cells recognize antigens derived from microbe‐derived intermediates of the riboflavin (microbial vitamin B_2_) synthesis pathway that are presented by MHC Class I‐related protein 1 (MR1) [3]. MAIT cells can be activated in a T‐cell receptor (TCR)‐dependent or TCR‐independent manner. TCR–MR1 signaling can fully activate MAIT cells, and costimulation with CD28, Toll‐like receptor (TLR) agonists, bacterial products, or cytokines increases their activation. Activated MAIT cells induce rapid innate‐like effects and then produce proinflammatory cytokines such as interferon (IFN)‐γ, tumor necrosis factor (TNF)‐α, interleukin (IL)‐17, and the cytotoxic molecules granzyme B (GzmB) and perforin [4]. The expression of chemokine receptors and integrins on MAIT cells allows them to migrate to inflammatory tissues [5, 6]. MAIT cells can migrate to the peripheral blood or tissues and exert anti‐infective effects [7].

**FIGURE 1:**
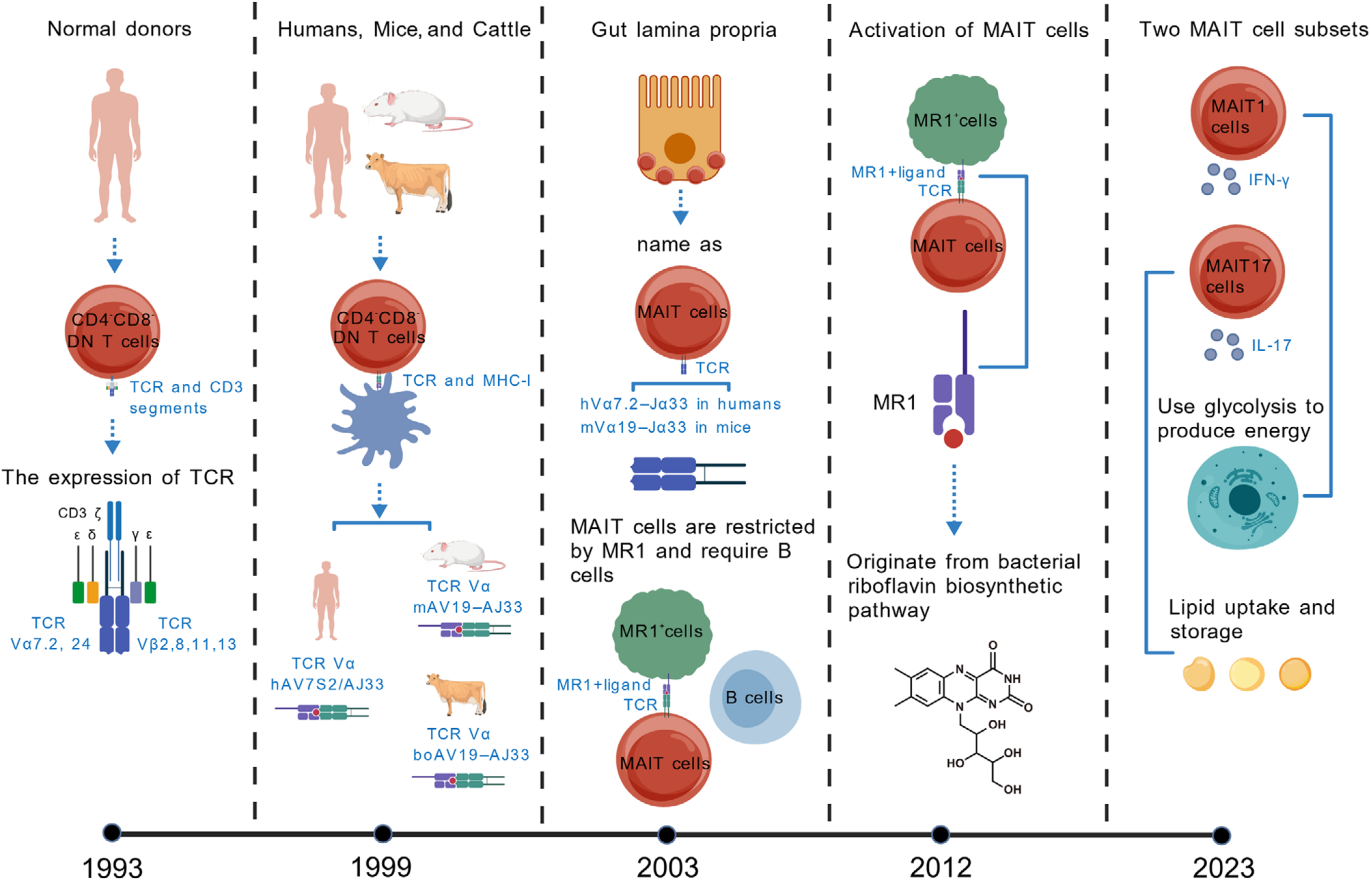
History of research on MAIT cells. The figure illustrates five milestones related to the discovery of MAIT cells, ranging from their phenotype, TCR rearrangement, restriction by MR1, and the different transcriptomes and metabolic states of two MAIT cell subsets. *Abbreviation*: DN T, double negative T cells.

MAIT cells have been studied for over three decades. In 1993, Porcelli et al. purified CD4^−^CD8^−^ (double negative, DN) α/β T cells from five normal donors and assessed their TCR expression, reporting that the expression of TCR Vα 7.2, 24 and TCR Vβ 2, 8, 11, and 13 was markedly increased [8]; however, the specific antigens or antigen‐presenting molecules for their recognition were unknown. In 1999, Tilloy et al. reported a new T‐cell population that existed in humans, mice, and cattle [9]. These cells were CD4^−^CD8^−^ DN T cells that express a canonical TCR α chain containing hAV7S2 and AJ33 in humans and the homologous AV19–AJ33 in mice and cattle with a constant length complementarity determining region 3 (CDR3). In 2003, Treiner and colleagues revealed that T cells expressing the canonical TCR segments hVα7.2–Jα33 or mVα19–Jα33 were preferentially located in the lamina propria of gut tissues; these T cells were named MAIT cells [10]. Furthermore, they reported that these MAIT cells were selected and restricted by MR1 and that their expansion required B lymphocytes, indicating that MAIT cells play a role in the antipathogen response. Further work in 2012 revealed that MR1 was ideally suited to bind to ligands originating from vitamin metabolites, and MR1‐restricted vitamin derivatives originated from the bacterial riboflavin (vitamin B2) biosynthetic pathway, which could specifically and potently activate MAIT cells and alter immunosurveillance [11]. An investigation of the developmental pathways of MAIT cells revealed that two predominant types of MAIT cells, a subset that produces IFN‐γ (MAIT1) and a subset that produces IL‐17 (MAIT17), have different transcriptomes and metabolic states. MAIT1 cells maintain a low‐energy steady state before they are activated, and the activated MAIT1 cells rely on glycolysis for energy production. In contrast, MAIT17 cells are highly activated and exhibit increased lipid uptake and lipid storage. These different metabolic processes drive MAIT cells to use different mechanisms to fight microbial infection [12, 13] (Figure 1).

As unconventional T cells, MAIT cells can maintain homeostasis in healthy individuals [5, 14]. Tissue repair is a critical function of MAIT cells, and MAIT cells express tissue repair‐associated genes such as IL‐17, IL‐22, vascular endothelial growth factor B (VEGFB), and granulocyte‒macrophage colony‐stimulating factor (GM‐CSF) to improve barrier integrity [5]. Moreover, the maintenance of MAIT cells in healthy individuals can be supported by a diverse gut microbiome, which was confirmed by the ability of metabolites from aerotolerant bacteria in the colonic mucosa to activate MAIT cells under physiological conditions [15]. Furthermore, MAIT cells can upregulate the expression of CD69, CD103, human leukocyte antigen‐DR (HLA‐DR), and programmed cell death protein 1 (PD‐1) to obtain tissue residence and an activated phenotype and to exhibit a multifunctional response profile [16]. The regulation of tissue structure and function can be achieved directly through the modulation of the proliferation and survival of structural cells, the differentiation of stem cells, and the expression of tight junction proteins or indirectly through the recruitment and differentiation of immune cells that promote repair [17]. In addition to relying on MR1, proinflammatory cytokines are needed for initiating and amplifying tissue repair programs in the steady state [18].

Alternatively, owing to their unique MR1‐mediated recognition capabilities and responsiveness to cytokine stimulation, MAIT cells play significant protective roles against various infections and immune‐related diseases [19]. After infection, MAIT cells are activated through direct recognition of microbial ligands or stimulation by inflammatory cytokines and then secrete cytokines such as IFN‐γ, TNF‐α, and IL‐17 as well as cytotoxic factors to induce antibacterial immune responses or inhibit viral replication [20]. Moreover, cytokines induce the activation of MAIT cells in the context of various immune‐mediated chronic diseases through the production of large amounts of cytokines and cytotoxic molecules, and increased expression of chemokine receptors and integrins on MAIT cells can promote the recruitment and activation of other immune cells, such as monocytes [21]. However, MAIT cells have shown pathogenic roles in certain contexts. For instance, MAIT cells can increase the fibrogenic function of hepatic myofibroblasts or primary human hepatic stellate cells after liver injury, which is characterized by increased cell proliferation and collagen secretion and activated proinflammatory responses [22, 23]. Additionally, activated MAIT cells secrete proinflammatory cytokines and cytotoxic factors that can lead to immune‐mediated damage in hosts and are associated with disease severity [14, 24, 25]. Thus, balancing the protective and pathogenic effects is a significant challenge for utilizing MAIT cells as targets for immunotherapy.

Although the general functions of MAIT cells have been elucidated, their unique regulatory roles in healthy individuals or individuals with various diseases have not. In addition, technical developments and applications related to MAIT cells are rapidly progressing. Thus, a systematic exploration of the characteristics and detailed effects of MAIT cells in health and disease is needed. In this review, we first describe the characteristics of MAIT cells, including their differentiation, activation mechanisms, and distributions. Next, we elucidate the regulatory roles of MAIT cells in healthy individuals and in individuals with diverse microbial infections and immune‐related diseases. In addition, we focus on the technological development and application of MAIT cells in disease contexts. This review not only provides a reference for investigators and medical personnel to understand the multifaceted effects of MAIT cells but can also be used to develop novel strategies for treating various diseases.

## 2 Fundamental Characteristics of MAIT cells

As an evolutionarily conserved cell population, MAIT cells express a unique semi‐invariant TCR consisting of an invariant TCRα chain and a restricted TCRβ chain. Changes in the expression of the TCRαβ chains and MAIT cell phenotypes occur throughout the entire differentiation process. Moreover, the unique activation mode of MAIT cells indicates that they can use specialized MHC Class I‐like molecules to recognize nonpeptide antigens. MAIT cells and MR1‐expressing cells are widely distributed in human and mouse tissues. The detailed fundamental characteristics of MAIT cells are described below.

### 2.1 Phenotypes of MAIT Cells and Their Development

MAIT cells are an evolutionarily conserved subpopulation of T cells with a unique semi‐invariant TCR consisting of an invariant TCRα chain and a limited TCRβ chain. In humans, MAIT cells express TCR Vα7.2–Jα33/Jα12/Jα20 paired with restricted Vβ2 or Vβ13, whereas in mice, TCR Vα19–Jα33 is paired with restricted Vβ6 or Vβ8 [7]. Mature MAIT cells also highly express a C‐type lectin‐like receptor (CD161); thus, the phenotype of these MAIT cells is CD3^+^ Vα7.2^+^ CD161^hi^. According to the expression of CD4 and CD8, MAIT cells can be categorized into five subpopulations, namely, CD4^+^ CD8^−^, CD4^+^ CD8^+^, CD4^−^ CD8αα^+^, CD4^−^ CD8αβ^+^, and CD4^−^ CD8^−^. In human peripheral blood, CD4^−^ CD8αα^+^ or CD4^−^ CD8αβ^+^ MAIT cells account for approximately 80% of the total MAIT cells, whereas CD4^−^ CD8^−^ MAIT cells account for approximately 15%, and the CD4^+^ CD8^−^ and CD4^+^ CD8^+^ subsets account for a small fraction (less than 5%) [24].

**FIGURE 2:**
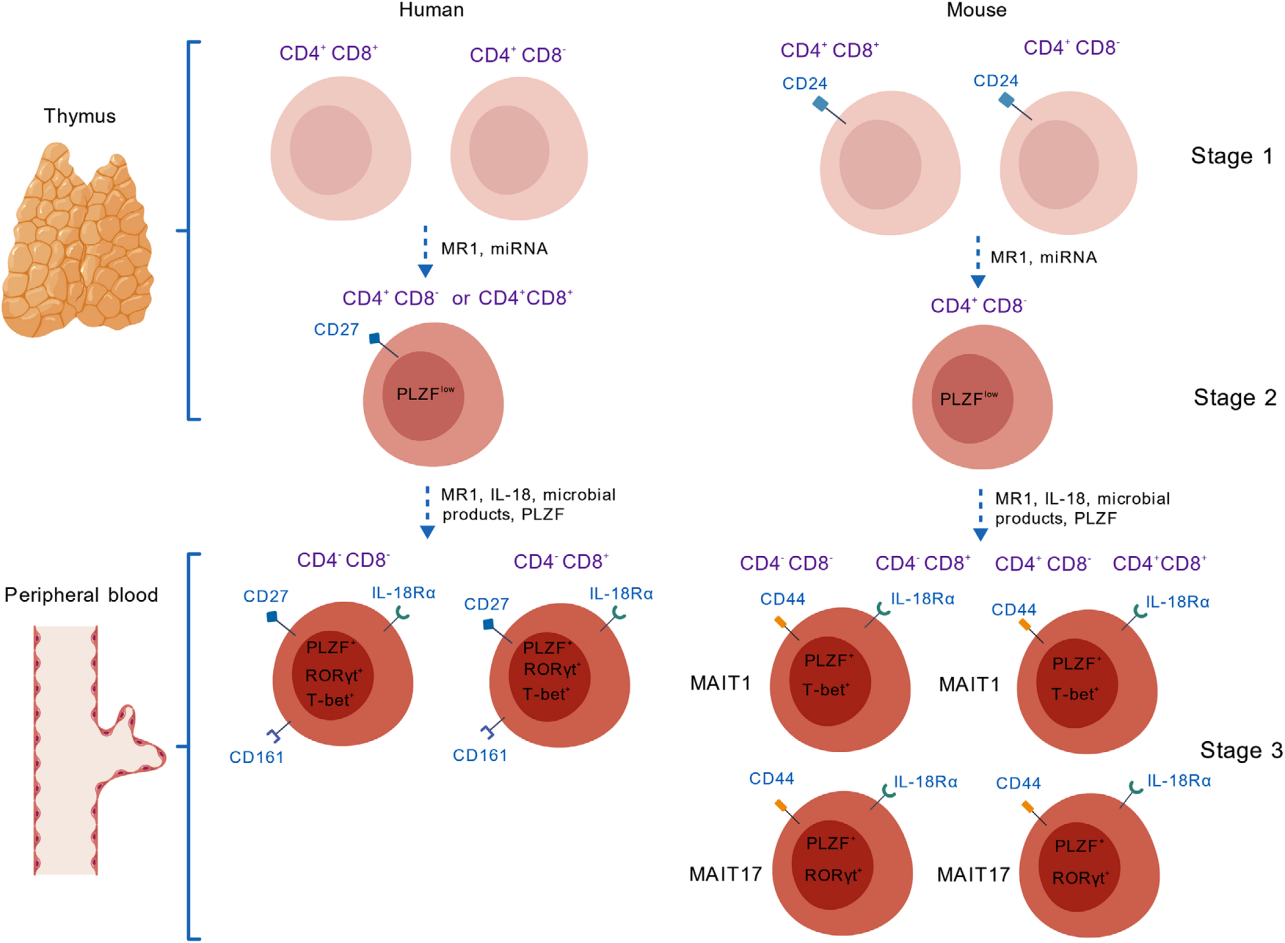
Three stages of MAIT cell differentiation and development in humans and mice. MAIT cells originally develop in the thymus. After positive selection, MAIT cells undergo intrathymic differentiation and then form CD4^+^ CD8^+^ and CD4^+^ CD8^−^ cell populations in Stage 1, while CD24 is highly expressed by MAIT cells in this stage in mice. By regulating microRNAs (miRNAs), MAIT cells express low levels of PLZF and exhibit CD4^+^ CD8^−^ (human and mouse) or CD4^+^ CD8^+^ (human) phenotypes in Stage 2; additionally, CD27 is highly expressed in human MAIT cells. PLZF is a known suppressor of the naïve T‐cell program and inducer of effector genes. After migrating into the peripheral blood, MAIT cells begin to express the transcription factor T‐bet and RORγt in humans, or either T‐bet or RORγt in mice. At this stage, both human and mouse MAIT cells highly express PLZF and IL‐18Rα. Additionally, human MAIT cells express CD161, and mouse MAIT cells express CD44 and IL‐18Rα. MR1 expression appears to be required at each stage of MAIT cell differentiation and development.

The maturation of MAIT cells can be divided into three stages [24, 26, 27]. In humans, the earliest cells (Stage 1) are defined as CD3^+^ and MR1 tetramer‐positive (MR1‐tet^+^), lacking the expression of the surface markers CD27 and CD161 and are predominantly CD4^+^ CD8^+^ or CD4^+^ CD8^−^ thymocytes. Developing MAIT cells in Stage 2 express CD27 but not CD161 and are mostly CD4^+^ CD8^−^ or CD4^+^ CD8^+^ thymocytes, with a small percentage migrate to the periphery. At this stage, MAIT cell development is regulated by microRNAs, which can also regulate promyelocytic leukemia zinc finger (PLZF) expression. After Stage 2, the progression of MAIT cells can depend on MR1, suggesting that microbial antigens play significant roles in this stage. At Stage 3, MAIT cells begin to migrate from the thymus to the periphery and are predominantly CD4^−^ CD8^−^ or CD4^−^ CD8^+^ cells that coexpress IL‐18 receptor alpha (IL‐18Rα), CD27, and CD161. Although different markers are used, the development of mouse MAIT cells closely parallels that of human MAIT cells. In mice, the earliest Stage 1 cells are also MR1‐tet^+^ and express CD24 but not CD44. In Stage 2, CD24 expression is deficient. MAIT cells begin to migrate to the peripheral blood for expansion at Stage 3, where they express classical markers such as IL‐18Rα, PLZF, and CD44. The expression of PLZF, IL‐18Rα, the transcription factor T‐Box transcription factor 21 (T‐bet) and the retinoid‐related orphan receptor γt (RORγt) indicates their functional maturation. An important distinction in MAIT cell development between mice and humans is the emergence of functional branches of MAIT cells at Stage 3 in mice; that is, the MAIT1 subseries and the IL‐17A‐producing MAIT17 subseries. Recent studies reported that most MAIT cells in the human thymus were CD8αβ, whereas those in the peripheral blood were mainly CD8αα, suggesting that CD8αα MAIT cells may have originated from CD8αβ MAIT cells [20, 24] (Figure 2).

The development of MAIT cells from stem cells to functional mature cells is influenced by the microbiota and depends on its secretion of riboflavin metabolites. Riboflavin metabolites such as 5‐(2‐oxopropylideneamino)‐6‐d‐ribitylaminouracil (5‐OP‐RU) can be transported to the thymus to trigger the thymic expansion of RORγt^+^ MAIT cells. Moreover, microbial colonization can normalize the MAIT cell levels in tissues such as the thymus and spleen. The initial microbiota colonization during early life can quickly establish and expand MAIT cells, promoting MAIT cell maturation in the peripheral tissues of hosts [28].

### 2.2 Activation of MAIT Cells

**FIGURE 3:**
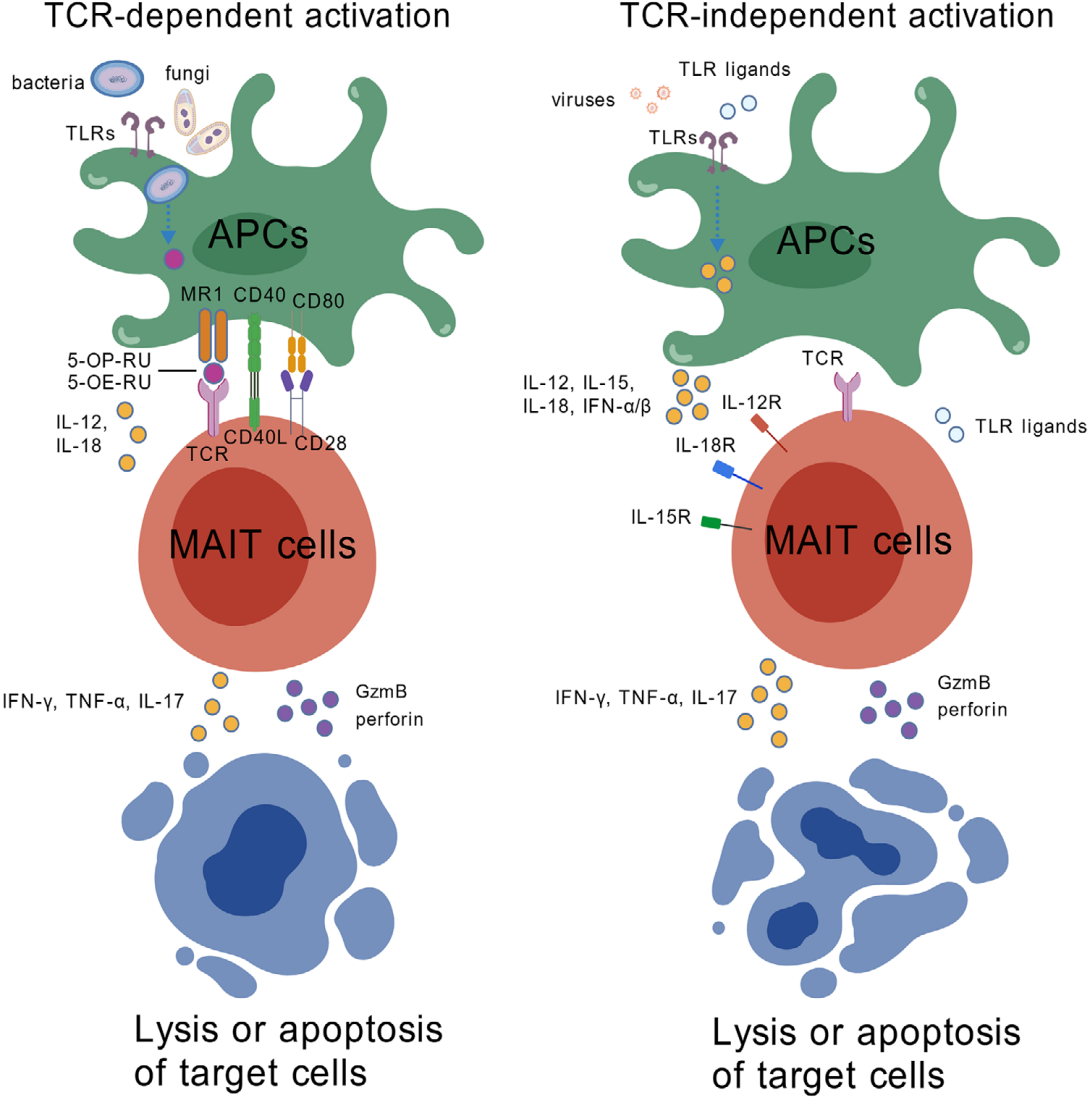
The processes of MAIT cell activation and its main effects. In the mode of TCR‐dependent activation, bacteria and fungi participate in the synthesis of riboflavin, the riboflavin intermediates are presented by MR1 under costimulatory signals and TLRs, and 5‐OP‐RU or 5‐OE‐AU is loaded into MR1 tetramers to identify the TCR and then mediate MAIT cell activation. With respect to TCR‐independent activation, MAIT cells can be directly activated by the cytokines IL‐12, IL‐15, and IL‐18, which are produced by APCs and in response to TLR ligands. After MAIT cells are activated, they secrete the cytokines IFN‐γ, TNF‐α, and IL‐17, as well as cytotoxic factors GzmB and perforin, which can kill and lyse the infected cells. *Abbreviation*: APC, antigen‐presenting cell.

MAIT cells are activated in a TCR‐dependent manner during infection by many bacteria and fungi, which have an intact riboflavin biosynthetic pathway, and the ligands produced by bacteria and fungi can be presented by MR1. However, some bacteria can also stimulate innate and adaptive immune cells to produce IFNs and cytokines, which directly activate MAIT cells. Besides, MAIT cells are activated solely by cytokines during viral infection because viruses cannot synthesize riboflavin‐derived ligands. For nonpathogenic diseases, MAIT cells can be activated by either cytokines or TCR–MR1 interactions, or synergy between the two modes. The synergy of the two activation modes occurs after the in vivo administration of synthetic 5‐OP‐RU to mice with the codelivery of TLR agonists, or after in the in vitro presentation of multiple ligands involving both TCR and IL‐12/IL‐18 signals [14]. The intermediate 5‐amino‐6‐d‐ribitylaminouracil (5‐A‐RU) produced in the riboflavin biosynthetic pathway is a highly unstable compound that can interact with α‐dicarbonyl compounds formed by mammalian glycolysis or bacterial metabolism, methylglyoxal (MG) and glyoxal, to produce 5‐OP‐RU and 5‐(2‐oxoethylideneamino)‐6‐d‐ribitylaminouracil (5‐OE‐RU), respectively, through nonenzymatic condensation reactions [29]. The generation of 5‐A‐RU requires pyrimidine deaminase/reductase, which is encoded by the key gene of the pathway, diaminohydroxyphosphoribosylaminopyrimidine deaminase/5‐amino‐6‐(5‐phosphoribosylamino) uracil reductase RibD (RibD). RibD expression is associated with the activation of MAIT cells, and bacteria that lack genes encoding components of this full riboflavin pathway, such as *Enterococcus faecalis* (*E. faecalis*) and *Listeria monocytogenes* (*L. monocytogenes*), are unable to activate MAIT cells [7, 30]. MR1 is a low polymorphic β2‐microglobulin (β2 m)‐associated antigen‐presenting molecule that is widely expressed in a variety of tissues. MAIT cells are restricted by MR1, recognize antigens presented by MR1, such as 5‐OP‐RU or 5‐OE‐RU, and are activated to induce immunological responses. MR1 binds to various metabolites of vitamin B, including the photodegradation product of vitamin B9 (folic acid), 6‐formylpterin (6‐FP) and its acylated form, acetyl‐6‐FP (Ac‐6‐FP), as well as pyrimidine‐based riboflavin intermediates and various ribityllumazines [11, 31]. Both 6‐FP and Ac‐6‐FP can upregulate the expression of MR1. However, they do not activate MAIT cells because they lack ribityl tails that can interact with the TCR of MAIT cells; instead, they competitively inhibit the activation of MAIT cells through binding to MR1 [31, 32]. Thus, 6‐FP and Ac‐6‐FP serve as inhibitory ligands for MAIT cells. The activation of MAIT cells is driven by MR1, which presents 5‐OP‐RU or 5‐OE‐RU to the semi‐invariant TCR, in conjunction with costimulatory signals from CD40–CD40L and CD80–CD28 binding. Besides, MAIT cell activation can also directly be driven by inflammatory cytokines produced from TLR‐expressing antigen‐presenting cells (APCs), IFNs, or TLR ligands. Once activated, MAIT cells secrete cytokines such as IFN‐γ, TNF‐α, and IL‐17 as well as cytotoxic factors such as GzmB and perforin to lyse or induce the apoptosis of target cells (Figure 3).

### 2.3 Distributions of MAIT and MR1‐Expressing Cells

**FIGURE 4:**
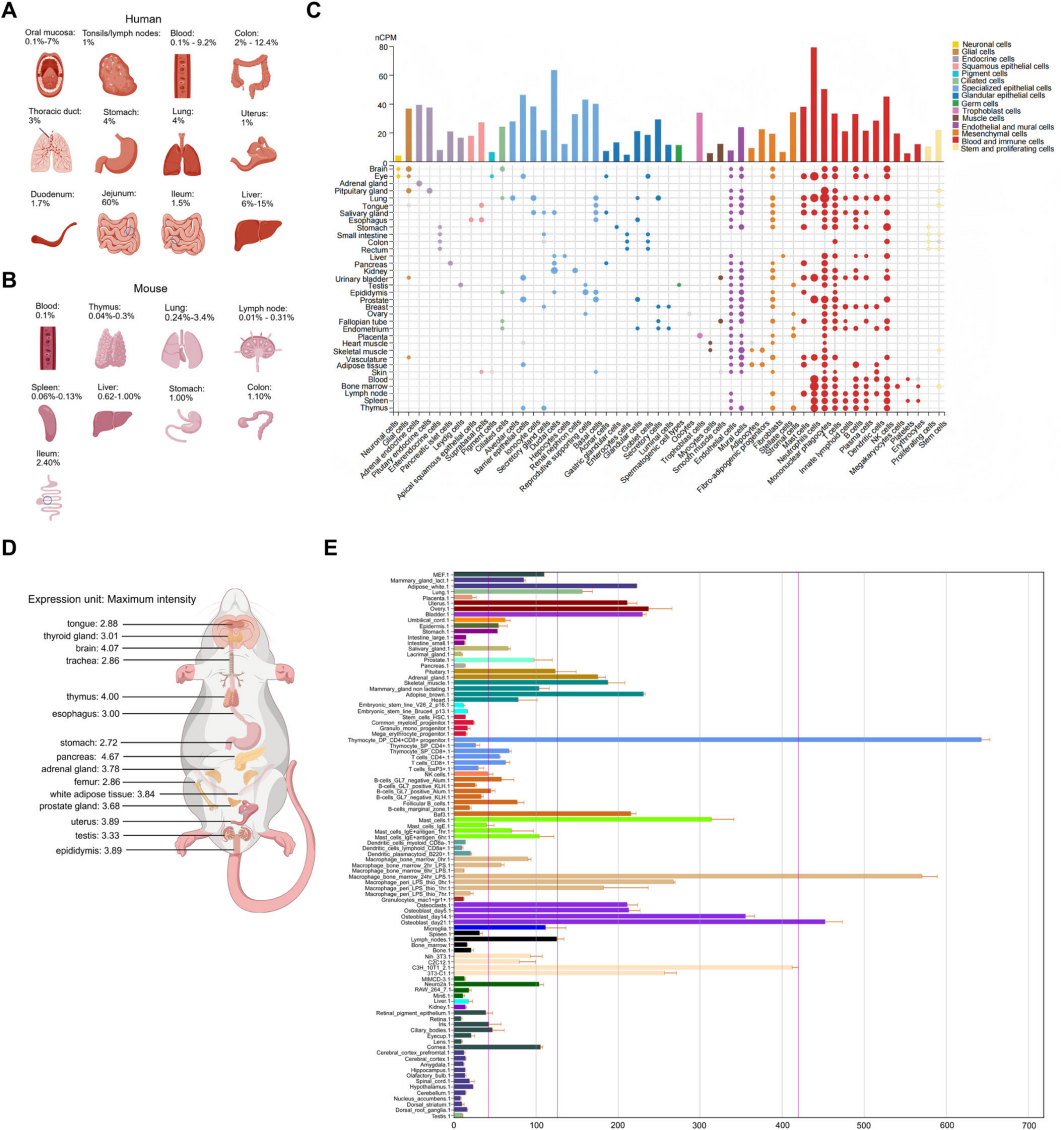
Distributions of MAIT cells in MR1‐expressing tissues and cells in humans and mice. (A) Proportions of MAIT cells among the total CD3^+^ T cells in each type of human tissue. (B) The proportions of MAIT cells among the total αβ T cells in each type of mouse tissue. (C) Expression levels of MR1 in human tissues and cells. The histogram shows the expression level of MR1 in each tissue, wherein the nCPM is used to reflect the level of MR1 expression in each cell type. The bubble diagram shows the expression level of MR1 in each cell type and tissue. The area of the circles reflects the proportion of MR1‐expressing cells among all cells. Data were obtained from the Human Protein Atlas [36, 37] (https://www.proteinatlas.org/ENSG00000153029‐MR1/single+cell), image available from v25.proteinatlas.org. (D) Expression levels of MR1 in mouse tissues. The maximum intensity was used to reflect the level of MR1 expression. Expression data of MR1 were obtained from the ProteomicsDB [38, 39, 40, 41] (https://www.proteomicsdb.org/protein/273369/summary), the image was designed and edited by BioGDP. (E) Expression levels of MR1 in mouse tissues and cells. Image was sourced from BioGPS (The Scripps Research Institute) [42, 43, 44] (http://biogps.org/#goto=genereport&id=15064;probeset:1421899_a_at), used under the terms of use, with all copyright notices retained.

MAIT cells are distinctly distributed in different human tissues. Among CD3^+^ T cells, MAIT cells account for 0.1–7% in the oral mucosa, less than 1% in the tonsils and lymph nodes, 0.1–9.2% in the blood, 2–12.4% in the colon, approximately 3% in the thoracic duct, 4% in the lung, 4% in the stomach, 1.7% in the duodenum, 1.5% in the ileum, and 1% in the uterus. The liver contains a relatively high proportion of MAIT cells, comprising 6–15% of the total CD3^+^ T cells. MAIT cells constitute the greatest proportion (up to 60%) of CD4^−^ CD3^+^ T cells in the jejunum (Figure 4A) [14, 19]. Moreover, in terms of total αβ T cells in mice, MAIT cells account for 0.04–0.3% in the thymus, 0.24–3.4% in the lung, 0.01–0.31% in the lymph nodes, approximately 0.1% in the blood, 0.06–0.13% in the spleen, 0.62–1.00% in the liver, 1.00% in the stomach, 1.10% in the colon, and 2.40% in the ileum [19, 33, 34, 35] (Figure 4B).

Furthermore, MR1 is widely expressed in various tissues and cell populations, as documented in the Human Protein Atlas database [36, 37] (https://www.proteinatlas.org/ENSG00000153029‐MR1/single+cell). In human body, high MR1 expression was observed among blood and immune cells within the eye, lung, salivary gland, stomach, urinary bladder, skin, blood, bone marrow (BM), lymph nodes, spleen, and thymus tissues. Compared with other blood and immune cells, neutrophils, mononuclear phagocytes, and natural killer (NK) cells have high normalized counts per million (nCPM) of MR1. These findings indicate that blood immune cells with high MR1 expression primarily activate MAIT cells (Figure 4C). Moreover, data from ProteomicsDB [38, 39, 40, 41] (https://www.proteomicsdb.org/protein/273369/summary) and BioGPS [42, 43, 44] (http://biogps.org/#goto = genereport&id = 15064; probeset: 1421899_a_at) revealed high MR1 expression were detected among pancreas, brain, thymus, uterus, adrenal gland, prostate gland, epididymis, and white adipose tissues in mice, and median or low expression of MR1 was detected in the thyroid gland, esophagus and testis. Additionally, high MR1 expression was also detected in thymocyte CD4^+^ CD8^+^ double‐positive cells, osteoclasts, and mast cells, and moderate or low MR1 expression was detected in B cells and CD4^+^ and CD8^+^ single‐positive (SP) T cells (Figure 4D,E). These findings suggest that MR1 is expressed mainly in digestive, endocrine, reproductive, and hematopoietic or immune cell lineage tissues.

Many questions about the effects and functions of MAIT cells in healthy individuals and patients have been raised. For instance, do these MAIT cells have the same or different activation modes or responses? What are the roles of microbial antigens in MAIT cell‐dependent responses? How does disease progression influence the protective or pathogenic roles of MAIT cells? The following section elaborates on the roles of MAIT cells in the context of different microbial infections and immune‐related diseases.

## 3 Effects of MAIT Cells in Healthy Individuals

As unconventional T cells, MAIT cells constitute an important part of the immunological microenvironment in mucosal tissues, such as the gastrointestinal tract and liver. MAIT cells play protective roles in maintaining mucosal integrity and repairing injured sites. The detailed mechanisms are discussed below.

### 3.1 Maintenance of Mucosal Homeostasis

Human mucosal tissues, such as the intestines, rectal mucosa, and vaginal epithelium, are colonized by commensal bacteria and contain MAIT cells. The ability of MAIT cells to home to these tissues is important for maintaining mucosal homeostasis [45]. The distinct distribution of MAIT cells in different parts of the gut is influenced by the varying compositions of bacteria in these sections, which depend on the amount of riboflavin secreted by certain microbiota taxa [5]. The microbiota serves as a rich source of riboflavin early in life and can promote intrathymic MAIT cell development and maturation, as well as sustain a life‐long population, all of which are beneficial for maintaining barrier function and the integrity of the microbial community [46]. MAIT cell homing to the gut is regulated by complex interactions involving chemokines, chemokine receptors, and tissue adhesion molecules, which are crucial for immune surveillance in mucosal tissues. During homeostasis, the homing of MAIT cells to the stomach relies on the chemokine receptor G protein‐coupled receptor 25 (GPR25) and its ligand CXCL17, whereas MAIT cell homing to the large intestine relies on the chemokine receptor G protein‐coupled receptor 15 (GPR15) and its ligand GPR15LG. Through the interaction of integrin α4β7 with mucosal addressin cell adhesion molecule‐1 or vascular cell adhesion molecule‐1 on endothelial cells, MAIT cells can transmigrate from the gut microvasculature [5]. Previous work has shown that MR1^−/−^ NOD mice exhibit increased intestinal barrier permeability, an increased number of goblet cells, and decreased expression of occludin and mucin 2 in gut epithelial cells [47]. Moreover, under steady‐state conditions, MAIT cells come into close contact with commensals in mucosal tissues without being activated by the riboflavin presented by MR1. One possible explanation for this phenomenon is that encountering only commensal‐derived antigens is insufficient to activate MAIT cells in the absence of inflammatory cytokines [45], which is supported by the observation IL‐15 can increase TCR‐mediated MAIT cell function by amplifying bacteria‐induced IFN‐γ secretion, granzyme production and cytolytic activity [48]. In addition, MAIT cells display a partially activated phenotype in normal liver tissues, but they can be quickly activated and dramatically increase Th1 and Th17 cytokine production in a TCR‐dependent manner in synergy with IL‐7 stimulation [49]. Although it is unclear why the effector function of MAIT cells is attenuated in healthy mucosal barrier tissues despite commensals continuously present antigens and basal inflammation remain, some unknown intrinsic and extrinsic mechanisms may help to regulate MAIT cell properties; therefore, further study is needed to clarify this issue.

### 3.2 Tissue Repair

MAIT cells play significant roles in maintaining tissue integrity and contributing to the regeneration of different organs. Owing to their abundance in the mucosa of tissues, MAIT cells can sense and respond to ligands produced by commensal populations, which may play a predominant role in maintaining barrier integrity without inflammation [17, 50]. MAIT cells serve as a scaffold for the migration and attachment of progenitor cells during wound healing [51]. MAIT cells largely exhibit a CD4^−^CD8^−^ phenotype and require IL‐23 for homeostasis [52]. Additionally, commensal *Staphylococcus epidermidis* colonizes the skin and promotes MAIT cell expansion in a TCR‐ and IL‐18‐dependent manner [52]. Tissue homeostasis maintained by MAIT cells can be achieved through their activated by endogenous or microbe‐derived ligands and then they express a tissue repair transcriptional program, or through the broad range of cytokines that activate MAIT cells [20, 50]. MAIT cells maintain tissue homeostasis and angiogenesis through the upregulation of the expression of signature genes such as TNF, colony stimulating factor 2 (CSF2), hypoxia inducible factor 1 subunit alpha, VEGFB, prostaglandin E synthase 2, platelet‐derived growth factor subunit B, transforming growth factor beta 1 (TGFB1), matrix metallopeptidase 25, high mobility group box 1, Furin, and CCL3 [53]. In gut tissue, tissue repair is performed by MAIT cells with a mixed Type 1/17 phenotype [20]. Skin excision experiments have shown that MAIT cells can migrate to wounds in a CXCR6‐dependent manner and promote keratinocyte proliferation and the secretion of amphiregulin to accelerate wound healing [54]. In addition to their roles in the skin and gut tissues, MAIT cells in the meninges of aged mice express high levels of antioxidant molecules and inhibit neuroinflammation in the brain, whereas the lack of MAIT cells results in increased levels of reactive oxygen species (ROS) in the meninges, reduced expression of junctional proteins, and leads to meningeal barrier leakage and cognitive function deficits [55].

### 3.3 Immunoregulation

MAIT cell effector function is strongly influenced by the local microenvironment. MAIT cells can be driven by antigen recognition and JAK/STAT signaling cytokines, such as IL‐27, IL‐21, and IL‐15, to secrete low levels of inflammatory cytokines and IL‐10; additionally, IL‐10 secretion can be increased by the addition of IL‐12 [56]. However, the combination of IL‐18 and antigen recognition can abrogate IL‐10 production by MAIT cells and induce IL‐17A expression instead. Additionally, IL‐10 secreted by MAIT cells can exert a paracrine effect by promoting the anti‐inflammatory polarization of monocytes [56]. MAIT cells can upregulate the expression of forkhead box protein P3 (FOXP3) during early and prolonged activation while simultaneously express the activation/exhaustion markers T‐cell immunoreceptor with Ig and ITIM domains (TIGIT), ectonucleoside triphosphate diphosphohydrolase 1, inducible T‐cell costimulator, and IL‐2 receptor subunit alpha, all of which are biomarkers of the conventional regulatory T cell lineage [57]. Moreover, MAIT cells can interact with other immune cells to exert immunomodulatory effects. MAIT cells can promote dendritic cell (DC) maturation in a CD40L‐ and MR1‐dependent manner, resulting in the secretion of IL‐12 [58].

In healthy individuals, MAIT cells home to distinct sites within mucosal tissues. Commensal bacteria in mucosal tissues secrete riboflavin to influence the distribution of MAIT cells. Activated MAIT cells help sustain the barrier integrity of mucosal tissues in a steady state. After injury occurs, MAIT cells migrate to the wound site to restore the target tissue by upregulating the expression of repair genes and promoting cell proliferation. Moreover, activated MAIT cells can exert immunomodulatory effects by upregulating FOXP3 and IL‐10, which may reduce the excessive inflammation induced by other immune cells. Collectively, these functions comprise a critical homeostatic mechanism to maintain health.

## 4 Effects of MAIT Cells After Microorganism Infection

**FIGURE 5:**
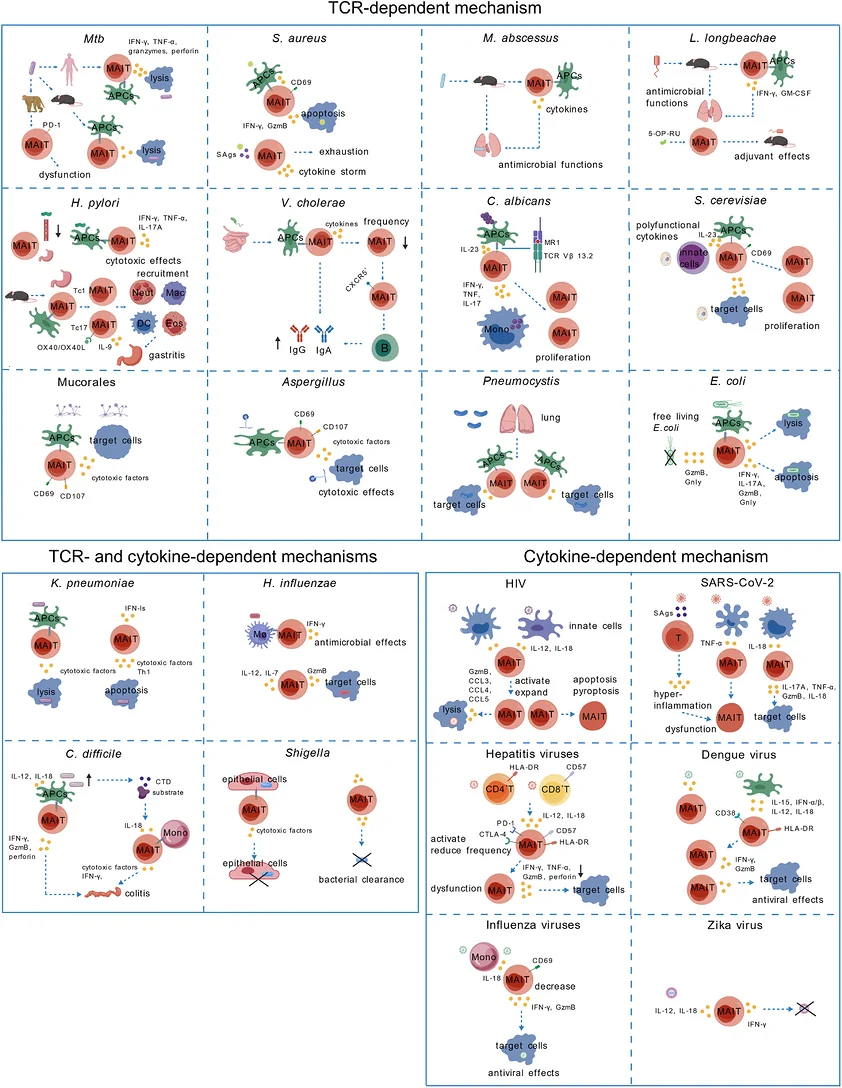
Responses of MAIT cells to microbial infection. Bacteria and fungi synthesize riboflavin intermediates, which are presented by MR1 to activate MAIT cells. Activated MAIT cells secrete cytokines and cytotoxic factors, which trigger infected cells to undergo apoptosis or lysis or recruit other immune cells to exert antimicrobial effects. In some circumstances, the upregulation of PD‐1 expression leads to MAIT cell dysfunction. MAIT cells can be activated by either ligands presented by MR1 or IFNs or cytokines secreted by immune cells in a TCR‐dependent or TCR‐independent manner. In the context of *C. difficile* infection, cytokines and cytotoxic factors secreted by MAIT cells play detrimental roles in inflammation‐driven immunopathology in colitis. During virus infection, MAIT cells can only be activated by IFNs and cytokines. Although the activated MAIT cells can secrete cytokines and cytotoxic factors to kill infected cells, cytokine dysregulation can lead to MAIT cell dysfunction under some circumstances. The SAgs from *S. aureus* and SARS‐CoV‐2 stimulate the secretion of a large quantity of cytokines by MAIT cells, and this cytokine release may lead to a cytokine storm. *Abbreviations*: *Mtb*, *Mycobacterium tuberculosis*; *S. aureus*, *Staphylococcus aureus*; *M. abscessus*, *Mycobacterium abscessus*; *L. longbeachae*, *Legionella longbeachae*; *H. pylori*, *Helicobacter pylori*; *V. cholerae*, *Vibrio cholerae*; *C. albicans*, *Candida albicans*; *S. cerevisiae*, *Saccharomyces cerevisiae*; *E. coli*, *Escherichia coli*; *K. pneumoniae*, *Klebsiella pneumoniae*; *H. influenzae*, *Haemophilus influenzae*; *C. difficile*, *Clostridium difficile*; HIV, human immunodeficiency virus; SARS‐CoV‐2, severe acute respiratory syndrome coronavirus 2; ZIKV, Zika virus; SAgs, superantigens; Neut, neutrophil; Mac, macrophage; DC, dendritic cell; Eos, eosinophil; Mono, monocyte; B, B cell; T, T cell; APC, antigen‐presenting cell; GM‐CSF, granulocyte‒macrophage colony stimulating factor.

As described above, MAIT cells can respond to infection by various bacteria, fungi, and viruses. Upon activation, MAIT cells can secrete IFN‐γ, IL‐17, GzmB, and other effector molecules, leading to the lysis of the infected cells to combat various infectious diseases, although their frequency and function can be altered (Figure 5). Additional details are provided in the following sections.

### 4.1 TCR‐Dependent Mechanism

#### 4.1.1 Mycobacterium Tuberculosis

*Mycobacterium tuberculosis* (*Mtb*) is the pathogen that causes tubercular diseases, and although 90% of *Mtb*‐infected individuals can immunologically control *Mtb* infection, tuberculosis (TB) remains a major concern because *Mtb* patients coinfected with human immunodeficiency virus (HIV) are at high risk of death [59]. MAIT cells respond to mycobacterial antigens in a TCR‐dependent manner, with several exceptions. For example, the partially efficacious TB vaccine bacille Calmette‐Guérin (BCG) activates MAIT cells to induce IFN‐γ in vitro in a TCR‐independent manner, and this activation is mediated by IL‐12 and IL‐18 [60]. In *Mtb*‐infected patients, the proportion and absolute number of MAIT cells in the peripheral blood are significantly lower than they are in healthy controls, suggesting that MAIT cells may be recruited to the site of infection during the course of infection [61]. MAIT cells are activated at the site of infection to produce IFN‐γ, TNF‐α, granzymes, and perforin. These effector factors can lyse infected cells and exert antimicrobial effects [30, 62, 63, 64]. After MAIT cell activation upon exposure to *Mtb*, CD4^+^ MAIT cells are relatively enriched early, but CD8^+^ MAIT cells are relatively scarce, suggesting that different subsets of MAIT cells exhibit distinct response profiles [65]. The number of MAIT cells is reduced in the circulation but increased in the upper respiratory tract of patients with active TB infection compared with those in healthy donors [66]. The frequency of endogenous MAIT cells in the lungs, which constitute 1–2% of TCRβ^+^ cells and are unaltered after *Mtb* infection, does not affect host protection against *Mtb* infection following prophylactic vaccination with 5‐OP‐RU [67]. MAIT cell vaccination before infection delays the initiation of *Mtb*‐specific CD4^+^ T cells in a TGF‐β‐dependent manner. However, in the context of chronic *Mtb* infection, MAIT cell expansion is promoted, triggering an IL‐17A‐dependent reduction in the bacterial load upon exogenous vaccination with 5‐OP‐RU [67]. In addition, *Mtb* infection can result in the overexpression of bifunctional riboflavin biosynthesis GTP cyclohydrolase II (ribA2), a key gene in the riboflavin synthesis pathway. ribA2 promotes MAIT cell activation, and the increased abundance of metabolites in this pathway reduced *Mtb* toxicity in a murine model of TB infection [68]. In a study of simian immunodeficiency virus (SIV) and *Mtb* coinfection in a crab‐eating monkey model, increased expressions of PD‐1 and the tyrosine inhibitory motif structural domains of TIGIT are observed in MAIT cells. This upregulation impaired the ability of MAIT cells to secrete TNF‐α [59]. Further study reveals that treatment of *Mtb*‐infected rhesus monkeys with 5‐OP‐RU does not increase the number of MAIT cells but instead results in MAIT cell dysfunction, as evidenced by the upregulation of PD‐1 expression and the loss of the ability to produce a variety of cytokines, a phenotype resembling T‐cell exhaustion [69]. Another study reports that the frequency of MAIT cells does not consistently change in the peripheral blood of *Mtb*‐infected macaques, whereas a notable increase in non‐MAIT CD8^+^ T cells is detected [70]. When 5‐OP‐RU is loaded onto MR1 tetramers, only some of the *Mtb*‐infected macaques present a detectable increase in the number of MAIT cells in bronchoalveolar lavage fluid (BALF), and lower expression of Ki‐67 and GzmB was detected. The tissue residency of MAIT cells is correlated with CD69 expression rather than bacterial load [71]. 5‐OP‐RU treatment does not increase infection control in *Mtb*‐infected rhesus monkeys, whereas treatment of *Mtb*‐infected mice with 5‐OP‐RU promotes MAIT cell expansion and reduced the bacterial load in the lungs [67], which may reflect fundamental differences in the biology of MAIT cells between these two species. The markedly distinct outcomes of 5‐OP‐RU treatment in mice and macaques are a significant concern and may be due to distinct microbiota compositions or a history of MAIT cell stimulation. Overall, the higher expression of PD‐1 in the MAIT cells of macaques than in those of mice may be related to the differences in TCR signaling between these two species, and persistent TCR stimulation under homeostasis leads to different functional states because of differences in TCR levels [69]. Owing to the similar phenotypes and functions of MAIT cells in human and nonhuman primates [72], the distinct outcomes of the effects of MAIT cells in *Mtb*‐infected macaques hinder the development of targeted therapies using MAIT cells to treat *Mtb* infection [69, 71]. Thus, the precise regulatory mechanisms of MAIT expansion in macaques need to be fully elucidated, which represents a critical step in obtaining expanded MAIT cells, to verify whether large populations of MAIT cells can play a protective role in *Mtb*‐infected macaques.

#### 4.1.2 Staphylococcus Aureus

The bacterium *Staphylococcus aureus* (*S. aureus*) is the main cause of life‐threatening toxic shock syndrome in humans [73]. During *S. aureus* infection, MAIT cell activation does not occur by cytokines alone through an innate‐like response but depends mainly on direct cell–cell contact mediated by TCR–MR1 and IL‐12 [2]. Activated MAIT cells produce IFN‐γ, GzmB, and CD69, exhibiting a more cytotoxic phenotype, and undergo significant degranulation, ultimately leading to the apoptosis of *S. aureus*‐infected cells and reducing the persistence of *S. aureus* within these cells [74]. However, *S. aureus* can secrete superantigens (SAgs), and MAIT cells can unexpectedly contribute to the SAg response in a cytokine‐driven manner; this process can cause a systemic inflammatory cytokine storm and render MAIT cells hyporesponsive [73]. After SAg stimulation, MAIT cells exhibit an exhausted phenotype and do not participate in antimicrobial defense [75].

#### 4.1.3 Mycobacterium Abscessus

*Mycobacterium abscessus* (*M. abscessus*) is the infectious agent of nontuberculous mycobacterial lung disease [76]. Patients infected with *M. abscessus* often have chronic lung diseases or impaired immune systems, and treatment of *M. abscessus* is less effective because of the multidrug resistance of this pathogen, which can evade most therapeutic approaches [77]. Previous report has indicated that MAIT cells play protective roles in *M. abscessus*‐infected mice in an MR1‐dependent manner [78]. MAIT cells can exert antimicrobial functions in the context of *M. abscessus*‐infected mice [79]. When MAIT cells are reprogrammed into induced pluripotent stem cells (iPSCs) and then undergo redifferentiation, they can protect hosts against *M. abscessus* infection [78].

#### 4.1.4 Legionella Longbeachae

*Legionella* is a significant cause of Legionnaires’ disease globally; elderly and immunosuppressed individuals and individuals with preexisting lung disease are more susceptible to *Legionella longbeachae* (*L. longbeachae*) infection, which results in symptoms of community‐acquired pneumonia [80]. In a mouse model of *L. longbeachae* infection, MAIT cells can be activated in an MR1‐dependent manner, rapidly accumulate at the site of pulmonary infection, and then subsequently secrete IFN‐γ and GM‐CSF to protect against *L. longbeachae* infection [81]. Additionally, the frequency of MAIT cells can be increased in mice by exposure to the 5‐OP‐RU ligand before infection with *L. longbeachae*, suggesting that MAIT cells can be used as potential adjuvants [82].

#### 4.1.5 Helicobacter Pylori

*Helicobacter pylori* (*H. pylori*) infection is the main cause of gastroduodenal ulcer disease and gastric carcinoma; *H. pylori* can induce chronic gastritis and, in turn, promote the development of dyspepsia, ulcers, gastric carcinoma, and mucosa‐associated lymphoid tissue lymphoma [83]. MAIT cells play both protective and pathogenic roles under distinct conditions following *H. pylori* infection. MAIT cells with CD8^+^ and CD4^−^ CD8^−^ phenotypes respond to *H. pylori* infection in an MR1‐dependent manner, and MAIT cells can secrete IFN‐γ, TNF‐α, and IL‐17A to exert cytotoxic effects. Although the frequency of blood MAIT cells in *H. pylori*‐infected individuals was significantly lower than that in uninfected individuals, there was no significant difference in the frequency of gastric MAIT cells between *H. pylori*‐infected and uninfected individuals [84]. Furthermore, in an *H. pylori*‐infected mouse model, MAIT cells contribute to chronic gastritis in an MR1‐dependent manner, and these gastric MAIT cells exhibit an effector memory Type 1 cytotoxic T lymphocyte/Type 17 cytotoxic T lymphocyte (Tc1/Tc17) phenotype and are associated with the acceleration of gastritis characterized by increased recruitment of neutrophils, macrophages, DCs, eosinophils, and non‐MAIT T cells [34]. Finally, OX40/OX40L signaling can promote mucosal MAIT cell proliferation and IL‐9 secretion in the context of *H. pylori*‐induced gastritis, which leads to mucosal inflammation and aggravates mucosal injury [85].

#### 4.1.6 Vibrio Cholerae

*Vibrio cholerae* (*V. cholerae*) is the causative agent of cholera, a severe diarrheal disease transmitted through contaminated water, food, and contact with infected individuals. Untreated patients may quickly die following *V. cholerae* infection [86]. Clinical data have shown that *V. cholerae* infection activates MAIT cells but decreases their frequency and increases the levels of IgA and IgG antibodies in patients [87]. MAIT cells primarily accumulate in the lamina propria of the duodenum of patients with culture‐confirmed *V. cholerae* O1 infection, and the depletion of duodenal MAIT cells increase intestinal permeability and inflammation [88]. Additionally, MAIT cells with a CXCR5^+^ T follicular helper (Tfh) phenotype can provide B‐cell help by facilitating *V. cholerae*‐specific IgA responses after mucosal infection with this pathogen [89]. These findings suggest that MAIT cells play protective roles against *V. cholerae* infection.

#### 4.1.7 Candida Albicans

*Candida albicans* (*C. albicans*) is a commensal fungus that colonizes the genital or gastrointestinal mucosa of individuals [90, 91]. In most healthy individuals, *C. albicans* cannot cause disease, but in immunodeficient hosts, such as HIV‐positive individuals who have not undergone antiretroviral (ARV) therapy (ART), *C. albicans* becomes pathogenic and is highly concerning [92]. Compared with those of bacteria such as *Escherichia coli* (*E. coli*), the recognition of *C. albicans* by MAIT cells is less sensitive; MAIT cells stimulated with *C. albicans* secrete lower levels of IFN‐γ, TNF, and IL‐17, while the MAIT cells that respond to *C. albicans*‐fed monocytes have a different TCR Vβ bias; in addition, Vβ13.2^+^ MAIT cells display slightly greater MR1‐dependent secretion of IFN‐γ and TNF [30, 93]. In individuals with multiple sclerosis (MS), exposure to *C. albicans* induces the extensive proliferation of activated MAIT cells, and 50% of the MAIT cells from MS patients are activated by IL‐23, which results not only in the upregulation of CD69 expression but also in increased MAIT cell proliferation [94].

#### 4.1.8 Saccharomyces Cerevisiae

As a food‐grade budding yeast, *Saccharomyces cerevisiae* (*S. cerevisiae*) has been used in many fields, such as fermentative foods, beverages, and biofuels, as well as in the fermentation industry and as a model organism in the laboratory [95]. MAIT cells stimulated by *S. cerevisiae* upregulate CD69 expression and increase their proliferation. Furthermore, MAIT cells can be activated by the fungal‐induced IL‐23 secretion by innate immune cells [94].

#### 4.1.9 Mucorales

Mucorales are pathogenic microorganisms that cause invasive fungal disease and threaten people's lives [96]. Previous studies have shown that Mucorales species such as *Mucor circinelloides* (*M. circinelloides*), *Rhizopus arrhizus* (*R. arrhizus*), and *Rhizopus microspores* (*R. microspores*) can stimulate rapid responses in human MAIT cells, which is followed by the upregulation of CD69 and CD107a expression, whereas the expression of intracellular perforin and granzyme A are reduced; the inhibition of MR1 can abrogate MAIT cell activation [97].

#### 4.1.10 Aspergillus

*Aspergillus* is another pathogenic microorganism that causes various diseases ranging from allergic syndrome to chronic pulmonary pathogenesis and invasive infections [98]. *Aspergillus* conidia can activate MAIT cells in a TCR‐dependent manner, and the increases in CD107a expression, degranulation, and release of cytolytic proteins such as perforin and granzymes indicate the degranulation of intracellular vesicles in activated MAIT cells. Such activation is mediated by APCs and is dependent on cell–cell contact [99].

#### 4.1.11 Pneumocystis

Pneumocystis is a pathogenic fungus that causes potentially life‐threatening pneumonia in immunodeficient individuals, such as HIV‐infected patients or those with malignancies, transplants, and autoimmune diseases [100]. In C57BL/6 mice, Pneumocystis infection increased the number of MAIT cells in lung tissue, which tended to continue to increase for many weeks after the Pneumocystis infection was cleared. However, in mice deficient in MAIT cells (MR1^−/−^), the clearance kinetics were similar to those in wild‐type C57BL/6 mice, indicating that MAIT cells may not be able to control Pneumocystis infection but may prolong their presence in the lungs, suggesting potential roles against other pathogens [100].

#### 4.1.12 Escherichia Coli

MAIT cells have shown direct antibacterial activity against *E. coli* that is dependent on TCR‐mediated MR1‐restricted antimicrobial activity [101]. MAIT cells can secrete IFN‐γ, IL‐17A, GzmB, and granulysins (Gnly) when cocultured with *E. coli*‐infected cells, thereby killing the infected cells in a cytolysin‐dependent manner. Moreover, the combined Gnly‐ and GzmB‐mediated antimicrobial activity of MAIT cells significantly increases the bactericidal activity of carbapenem antibiotics against carbapenem‐resistant bacteria such as *E. coli* [101]. Furthermore, although carbapenem‐resistant *E. coli* (CREC) requires treatment with a class of last‐resort antibiotics that can improve carbapenem permeability [102], MAIT cells can efficiently kill the free form of the CREC strains or CREC‐infected cells by secreting the Gnly and GzmB proteins and by reducing bacterial loads in cells in an MR1‐dependent manner [103]. In addition, MAIT cell‐derived Gnly and GzmB can restore the bactericidal efficacy of carbapenem antibiotics against CREC strains. A possible mechanism is that impairment of the cell wall caused by Gnly can increase carbapenem penetration into bacterial cells and decrease bacterial viability through GzmB [101, 104].

### 4.2 TCR‐ and Cytokine‐Dependent Mechanisms

#### 4.2.1 Klebsiella Pneumoniae

*Klebsiella pneumoniae* (*K. pneumoniae*) is the causative agent of many diseases, such as pneumonia, urinary tract infections, bacteremia, and liver abscesses [105]. A previous study identified genes encoding enzymes involved in riboflavin biosynthesis in *K. pneumoniae* [106], suggesting that MAIT cells may be activated via an MR1‐dependent mechanism. MR1 deficiency in mice was found to increase their susceptibility to *K. pneumoniae* [107]. However, recent studies have shown that in *K. pneumoniae*‐infected individuals, the activation of MAIT cells is independent of MR1 and driven primarily by Type I IFNs (IFN‐Is), and IFN‐I stimulation can induce a Th1/cytotoxic transcriptional program and modulate the localization of MAIT cells within the lungs [108]. The adoptive transfer of or increase in pulmonary MAIT cells can protect hosts against *K. pneumoniae* infection.

#### 4.2.2 Clostridium Difficile

*Clostridium difficile* (*C. difficile*) is a well‐established pathogen of both humans and animals that resides mainly in contaminated foods and the environment. *C. difficile*‐infected individuals present symptoms such as diarrhea, toxic megacolon, and colonic perforation and even face the threat of death [109, 110]. After *C. difficile* infection, this pathogen can synthesize riboflavin, thereby activating MAIT cells in an MR1‐dependent manner and synergizing with IL‐12 and IL‐18 to upregulate the expression of CD69, IFN‐γ, GzmB, and perforin. Furthermore, compared with low‐virulence strains, infection with hypervirulent *C. difficile* can provoke stronger MAIT cell activation and effector function, suggesting that MAIT cells play a detrimental role in inflammation‐driven immunopathology in nosocomial *C. difficile‐*associated colitis and may be used as a novel biomarker of disease severity [111]. Moreover, the binary toxin *C. difficile* transferase (CDT), which is produced by hypervirulent *C. difficile* strains, binds to its substrate to induce significant MAIT cell activation; this activation does not rely on MR1, which presents bacterial‐derived metabolite antigens; instead CD14^+^ monocytes can be targeted by CDT to induce MAIT cell activation and the secretion of cytotoxic factors in an MR1‐dependent manner [112]. IL‐17A and IFN‐γ proinflammatory cytokines are secreted by the MAIT cells of *C. difficile*‐infected patients [113].

#### 4.2.3 Haemophilus Influenzae

*Haemophilus influenzae* (*H. influenzae*) is a facultatively anaerobic, gram‐negative bacterium that causes chronic airway diseases in infected individuals and exacerbates the inflammatory response [114, 115]. Nontypeable *H. influenzae* infection (NTHi) causes chronic obstructive pulmonary disease (COPD) and is a likely target of MAIT cell immunity. NTHi‐infected macrophages can upregulate the expression of MR1 and activate MAIT cells to produce IFN‐γ, whereas treatment with steroids decreases the frequency of MAIT cells and impairs MAIT cell responses [116]. Moreover, without antigen presentation, IL‐12 and IL‐7 can synergistically control the production of GzmB to kill NTHi‐infected cells, suggesting that MAIT cells can be activated in a cytokine‐dependent manner [117].

#### 4.2.4 Shigella

*Shigella* is the cause of shigellosis, which can lead to invasive bloody diarrhea in children under 5 years of age. It is transmitted through person–person contact or the fecal–oral route through contaminated food and drinking water [118]. Infection of epithelial cells by the invasive bacterium *Shigella flexneri* (*S. flexneri*) can promote endogenous MR1 expression; the specific ligand produced by *Shigella* is presented to MR1 and activates MAIT cells; subsequently, infected epithelial cells can be killed by cytotoxic MAIT cells [119]. In MR1‐knockout mice, the pathogen *Shigella dysenteriae* (*S. dysenteriae*) is cleared at a normal rate, suggesting that MAIT cell activation upon *S. dysenteriae* infection occurs in a TCR‐independent manner [107].

### 4.3 Cytokine‐Dependent Mechanism

#### 4.3.1 Human Immunodeficiency Virus

HIV infection remains a serious global health concern, with 39 million people living with HIV infection worldwide in 2022, including 1.3 million newly infected individuals [120]. CD4^+^ T cells are the main target of HIV, and massive depletion of CD4^+^ T cells is a hallmark of HIV infection [121]. ART can reduce plasma HIV RNA levels below the detection limit in infected hosts. However, once treatment is interrupted, viremia will rebound within a few weeks because of the persistence of latent viral reservoirs in resting CD4^+^ T cells [122]. A previous study revealed that MAIT cells were strongly activated during HIV‐1 infection, and in vitro experiments revealed that the activation of MAIT cells was dependent on the coordinated effects of IL‐12 and IL‐18 [123]. Activated MAIT cells secrete GzmB and upregulate the antiviral chemokines CCL3, CCL4, and CCL5 to promote antiviral activity. CCL4 is the ligand for CCR5, a coreceptor of HIV‐1; by competitively binding to CCR5, CCL4 can prevent viral binding and entry into target cells, thereby inhibiting HIV‐1 infection [123].

Studies have shown that MAIT cells in peripheral blood are activated and expanded during early acute HIV‐1 infection but subsequently severely decrease in number and function [124, 125]. Successful ART only partially recovers the function of MAIT cells but fails to restore normal MAIT cell numbers in peripheral blood [124, 126, 127, 128]. In contrast to the loss of MAIT cells in the peripheral blood, MAIT cell numbers are relatively preserved in the cervical and intestinal mucosa of women, suggesting different degrees of MAIT cell depletion in different tissues [129, 130]. The mechanism of MAIT cell loss has not been fully elucidated but may be related to cytokine dysregulation, the sustained activation of MAIT cells by microbial metabolites due to microbial translocation, the downregulation of CD161 expression that impairs the function of MAIT cells, the recruitment of MAIT cells to inflammation sites, and activation‐induced apoptosis and pyroptosis [127, 131, 132]. In addition, MAIT cells are highly activated in the peripheral blood of HIV‐1‐infected patients, with the upregulation of markers related to systemic T‐cell activation, microbial translocation, and intestinal damage in patients receiving ART for the first time, as well as poor immune reconstitution in patients who have received ART long‐term [130]. Consequently, activation‐induced pyroptosis results in MAIT cell loss in HIV‐1‐infected patients, which may potentiate disease progression and poor immune reconstitution. Given that MAIT cells exhibit numerical loss and dysfunction after HIV infection, restoring their number and function may help improve mucosal and antiviral immunity and reduce the risk of microbial coinfections in HIV‐positive individuals. In a model of SIV‐infected rhesus monkeys, mucosal adenovirus (Ad)‐SIV recombinant vaccination increased the frequency of MAIT cells, as well as the expression of IFN‐γ and TNF‐α, and the expression of the proliferation marker Ki‐67 in the blood and BALF [133]. In HIV‐infected patients, the plasma IL‐7 level is positively correlated with the MAIT cell frequency, and treatment with IL‐7 helps to restore the number of MAIT cells in the peripheral blood of individuals with chronic HIV‐1 infection [134].

#### 4.3.2 Hepatitis Viruses

Hepatitis viruses can be categorized into five types: hepatitis A virus (HAV), hepatitis B virus (HBV), hepatitis C virus (HCV), hepatitis D virus (HDV), and hepatitis E virus (HEV). These viruses are RNA viruses, except for HBV, which is a DNA virus. HAVs and HEVs infections are usually self‐limiting, and a complete cure can be achieved in the majority of patients [135]. HDV infections require specific treatment, while HBV, HCV, and HDV infections can progress to chronic liver disease, ultimately leading to the development of cirrhosis and hepatocellular carcinoma (HCC) [136]. Studies have shown that in patients with chronic hepatitis virus infection, the frequency of MAIT cells in the peripheral blood is significantly reduced, and the expression of CD57, PD‐1, cytotoxic T‐lymphocyte‐associated antigen‐4 (CTLA‐4), and HLA‐DR and CD38 on MAIT cells is significantly increased, promoting the acquisition of an immune senescence phenotype. Additionally, the expression level of PD‐1 is positively correlated with the plasma level of HBV‐DNA, whereas the expression of T‐cell immunoglobulin and mucin domain 3 (TIM3) and PD‐1 is negatively correlated with the serum level of alanine transaminase. These findings suggest that PD‐1 expression on MAIT cells can play a significant role in persistent infection. Increased expression of PD‐1 impairs the function and decreases the frequency of MAIT cells by triggering exhausted and dysfunctional phenotypes [137, 138]. However, the frequency of MAIT cells is not related to the HBV viral load but is inversely related to HLA‐DR on CD4^+^ T cells or MAIT cells and CD57 on CD8^+^ T cells, suggesting that the depletion of MAIT cells may not be caused by direct HBV infection but may be driven by HBV‐induced chronic immune activation [137]. Furthermore, MAIT cells also exhibit dysfunction, which manifests as mainly low levels of IFN‐γ, TNF‐α expression [137]. Another study indicated that the numbers of circulating MAIT cells are significantly reduced in patients with HBV‐related liver failure, but plasma IL‐12 and IL‐18 levels are elevated in these patients, suggesting that the depletion of circulating MAIT cells might be attributable to IL‐12 and IL‐18 stimulation [139]. Furthermore, the number of MAIT cells is correlated with the severity of disease, which suggests that the number of circulating MAIT cells may serve as a predictor of HBV‐associated liver failure [139]. HCV‐induced responses can activate MAIT cells and limit HCV replication in an IFN‐γ‐dependent manner [140]. In patients with chronic HCV infection, the frequency of MAIT cells is significantly reduced in both the peripheral blood and liver, and after antiviral therapy, the frequency of MAIT cells in the liver increases whereas hepatic inflammation decreases, but the number of MAIT cells in the blood does not change. In addition, the function of MAIT cells in the peripheral blood is impaired after *E. coli* and HCV coinfection, suggesting that the defense mechanism of MAIT cells against bacterial infection is impaired [141]. Further study revealed that the frequency and function of circulating MAIT cells can be partially restored after successful antiviral treatment of single HCV infection [142]. Compared with those in healthy controls, the number of MAIT cells in the peripheral blood and liver is dramatically lower in patients with chronic HDV infection, but the levels of the proinflammatory cytokines IL‐12 and IL‐18 are increased, suggesting that these cytokines may contribute to MAIT cell activation and subsequent depletion during HDV infection [138]. Additionally, in response to *E. coli* stimulation, MAIT cells from HDV‐infected patients fail to upregulate CD69 and CD25 and produce lower levels of IFN‐γ, GzmB, and CD107a, suggesting that the function of MAIT cells in chronic HDV infection is impaired in response to MR1‐restricted bacterial antigens [138].

#### 4.3.3 Influenza Viruses

Influenza viruses are highly contagious respiratory viruses. Influenza is among the most prevalent and transmissible diseases worldwide; it causes seasonal influenza every year and severe illness and life‐threatening complications in high‐risk individuals [143]. Studies have shown that the number of MAIT cells in the peripheral blood of patients with severe influenza is significantly reduced and correlated with disease severity. In vitro coculture experiments revealed that the activation of MAIT cells is regulated by IL‐18 produced by influenza A virus, which infects CD14^+^ monocytes [144], and that this activation results in the upregulation of the expression of CD69, IFN‐γ, and GzmB in MAIT cells, which thus function as antiviral effectors with protective effects during human influenza virus infection [144, 145]. In an in vivo mouse experiment, compared with those in wild‐type mice, the accumulation and activation of MAIT cells in the lungs during influenza virus infection in MR1^−/−^ mice resulted in greater weight loss and higher mortality after influenza virus infection; these effects were ameliorated after the transfer of lung MAIT cells via intravenous transfusion, which demonstrated that MAIT cells play a host‐protective role during influenza virus infection and help prevent lethal viral attack [145].

#### 4.3.4 Severe Acute Respiratory Syndrome Coronavirus 2

Severe acute respiratory syndrome coronavirus 2 (SARS‐CoV‐2) infection has been a global pandemic with a high number of fatalities since 2020; infection with SARS‐CoV‐2 is associated with both mild symptoms and severe disease, and the immune system plays a significant role in controlling the progression of SARS‐CoV‐2 [146]. In acute SARS‐CoV‐2 infection, MAIT cells are highly activated, leading to the secretion of high levels of the proinflammatory cytokines IL‐17A and TNF‐α; moreover, the expression of the activation marker HLA‐DR is positively correlated with disease severity [146]. Other studies have shown that SARS‐CoV‐2 infection leads to a rapid decrease in the number of circulating MAIT cells in the peripheral blood. However, these cells are significantly enriched in the airways and display a strongly activated and cytotoxic phenotype; notably, the frequency and function of MAIT cells in the blood are positively correlated with the severity of SARS‐CoV‐2 infection and mortality [147, 148, 149]. In individuals with severe SARS‐CoV‐2 infection, the progressive IFN‐α shift to IL‐18 secretion is associated with the activation of MAIT cells and their cytotoxic phenotype [148]. In peripheral blood, MAIT cells with increased cytotoxicity, chemotaxis, and apoptosis are observed, which is consistent with disease severity in SARS‐CoV‐2 patients; furthermore, the frequency of pyroptotic MAIT cells increases, which is one of the leading causes of MAIT cell death after SARS‐CoV‐2 infection [150]. This may lead to a rapid decrease in the number of MAIT cells in peripheral blood. Moreover, the immunological dysfunction and depletion of MAIT cells during SARS‐CoV‐2 infection suggest that SAgs or SAg‐like proteins may trigger a superantigenic host response [151]. Previous studies have indicated that SARS‐CoV‐2 spike (S) proteins contain a sequence and structural motifs (the S1–S2 cleavage site) that are highly similar to those of staphylococcal enterotoxin B, which promotes the cytotoxic adaptive immune response through the binding of pathogenic SAgs to TCR and MHC‐II molecules. These sequences and structural motifs can directly bind TCRs and cause severe hyperinflammation in COVID‐19 patients, exaggerated T‐cell responses, and the formation of autoantibodies against cardiovascular, gastrointestinal, and endothelial antigens, which are the main characteristics of multisystem inflammatory syndrome [152, 153, 154]. The SAg‐like effect of the SARS‐CoV‐2 S protein can be identified by detecting V‐segment skewing in patients with severe COVID‐19 [155]. Another report indicated that a SARS‐CoV‐2‐infected woman diagnosed with pityriasis rubra pilaris may have been mediated by a SAg of SARS‐CoV‐2, given the clinical similarity of the prodromic scarlatiniform phase with other skin disorders mediated by the SAgs; although the superantigenic nature of SARS‐CoV‐2 is still controversial, at least one unique SAg‐like motif has been confirmed to exist within the virus [156].

Although MAIT cell activation and altered phenotypes and functions are associated with COVID‐19 severity, MAIT cells can also play crucial roles in the host response to SARS‐CoV‐2, and an increase in the number of MAIT cells in the lungs enhances the ability of MAIT cells to clear virus‐infected cells through the secretion of cytokines such as IL‐18, IL‐17A, and GzmB. In vitro coculture of MAIT cells with SARS‐CoV‐2‐infected macrophages confirmed these functions of MAIT cells [147, 148, 149, 157]. MAIT cells can synergize with SARS‐CoV‐2 vaccines to induce protective immunity. In a prime and twofold boost immunization approach involving immunization with inactivated SARS‐CoV‐2 vaccines, MAIT cells were observed to expand and differentiate over time, suggesting that antiviral factors promote MAIT cell activation [158]. Other types of SARS‐CoV‐2 vaccines, such as the mRNA vaccine BNT162b2, can present signatures of activation, proliferation and TNF expression, which are correlated with spike‐specific antibody responses; the transcriptional signatures of effector responses (*IFNG*, *TNF*, and *XCL2*); and the expression of memory‐associated genes (*IL7R*, *TCF7*, *GATA3*, *EOMES*, and *CXCR3*), all of which are also present in MAIT cells [159, 160]. In addition, MAIT cells can be used as potential adjuvants for immunization strategies. Rashu et al. combined 5‐OP‐RU with several vaccines for influenza viruses and SARS‐CoV‐2 based on recombinant vesicular stomatitis virus. Investigators reported that 5‐OP‐RU can be incorporated into select vaccine formulations and expand tissue MAIT cells, resulting in an inflammatory phenotype and eliciting conventional virus‐specific T‐cell responses [161]. Pankhurst et al. combined 5‐A‐RU and MG as adjuvants for SARS‐CoV‐2 spike targets and reported that they can induce protective humoral immunity and immunoglobulin A production and that the activity of MAIT cells is mediated by CD40L‐dependent activation of DCs, followed by the activation of Tfh cells [162].

#### 4.3.5 Dengue Virus

Dengue virus (DENV) is the causative agent of dengue fever, which is transmitted mainly through the bite of infected female *Aedes* species of mosquitoes. Dengue fever is the fastest‐spreading viral disease and mosquito‐borne infection in tropical and subtropical regions; in most individuals, the immune system is able to successfully control the replication of the virus within a few days of infection, and infection is thus asymptomatic, whereas in some populations, it may manifest as mild febrile dengue fever or progress to more severe dengue hemorrhagic fever and dengue shock syndrome [163]. Studies have demonstrated that MAIT cells can respond to DENV infection and that their activation is dependent on the synergistic effect of IL‐18 together with IL‐12, IL‐15, or IFN‐α/β [140]. Expression of the MAIT cell proliferation and activation markers Ki‐67, CD38, and HLA‐DR is elevated during acute DENV infection, but there is no significant difference in the MAIT cell frequency between DENV‐infected patients and healthy controls [164, 165]. In vitro, activated MAIT cells produce IFN‐γ and GzmB when cocultured with APCs treated with DENV, suggesting a potential role for MAIT cells in host defense against viral infection, although MAIT cells in the acute phase of DENV infection produce less IFN‐γ after *E. coli* stimulation [140, 164].

#### 4.3.6 Zika Virus

Zika virus (ZIKV) is a mosquito‐borne virus that causes neurological complications in adults and congenital malformations in infants [166]. Patients infected with ZIKV may exhibit lethargy with severe morbidity and dengue shock syndrome and may even die in some cases. In an in vitro ZIKV infection model, MAIT cells produced IFN‐γ in response to ZIKV, and the activation of MAIT cells was dependent on IL‐12 and IL‐18 but not MR1 [164].

### 4.4 Distinct Roles of Other Unconventional T Cells in Microbial Infections

In addition to MAIT cells, other unconventional T cells can also sense diverse lipids and peptides to activate adaptive immunity during microbial infections. These unconventional T cells can be grouped into three types: Type 1 T cells are characterized by semi‐invariant TCR usage, which includes Type 1 NK T (NKT) cells, MAIT cells, germline‐encoded mycolyl‐reactive (GEM) T cells and TCRγδ T cells (Vγ4, Vγ5, and Vγ7); Type 2 T cells are characterized by diverse TCR usage with respect to both the TCR gene segments and the CDR3 sequences, which includes H2–M3‐restricted T cells, HLA‐E‐restricted T cells, and TCRαβ CD8αα intraepithelial T lymphocytes (IELs); and Type 3 T cells are characterized by diverse TCRs that can bind to CD1 and MR1, which includes Type 2 NKT cells, MR1‐restricted T cells, and TCRγδ T cells [167].

NKT cells are an important subset of immune cells that can bridge the innate and adaptive immunity of hosts. Unlike MAIT cells, NKT cells can respond to lipid‐based antigens, which are restricted by CD1d. There are two different subsets of NKT cells, Type I NKT or invariant NKT (iNKT) cells, which carry a semi‐invariant Vα14/Jα18 with Vβ segments 2, 7, and 8 TCRs in mice and Vα24/Jα18 with Vb11 in humans, and Type II NKT cells, which have different TCRs on their cell surface [168]. In humans, Type II NKT cells are more common than Type I NKT cells are and can respond to diverse repertoires of lipid antigens, whereas iNKT cells have limited antigen specificity and reactivity to auto and foreign antigens [168]. During infection, iNKT cells can recognize microbial lipid antigens with their TCRs or, through a cytokine‐driven mechanism involving the recognition of CD1d, present autoantigens, which are similar to those in MAIT cells [169]. Additionally, the synergy of CD1d‐presented lipid antigens and cytokine‐mediated stimulation regulated by TLR ligands has been confirmed after infection with the spirochete *Borrelia burgdorferi* (*B. burgdorferi*) [170]. After activation, iNKT cells play protective roles in host defense against infections, several of which are pathogenic, such as *S. pneumoniae* and DENV infection [171, 172]. Type II NKT cells can either promote protective innate and adaptive immune responses, such as by reducing the pathogen‐induced cytokine storm and improving survival in *S. aureus* infection, or contribute to inflammation and pathogen‐mediated tissue injury, as observed after infection with *Schistosoma mansoni* (*S. mansoni*) and *Trypanosoma cruzi* (*T. cruzi*) [173]. Notably, compared with their mammalian counterparts, the diverse bacterial lipids recognized by Type II NKT cells could be more potent antigens [174].

GEM‐T cells constitute another conserved human T‐cell population; they carry an invariant TRAV1‐2/TRAJ9 TCRα chain and are restricted to CD1b [175, 176]. Like MAIT cells, the TCRs of GEM T cells are conserved, but they can recognize bacterial glycolipids such as mycolic acid (MA) or glucose monomycolate antigens presented by CD1b and secrete IFN‐γ and IL‐2 after stimulation [175]. During *Mtb* infection, MA can be esterified to glycerol (Gro‐MM), glucose (GMM), or trehalose (TMM) and presented by CD1b to activate GEM T cells. The conserved TCR within GEM‐T cells can respond to *Mtb* infection via clonal expansion and the secretion of antimycobacterial cytokines [177]. Although the frequency of GEM‐T cells is ∼10‐fold lower than that of NKT cells, GEM‐T cells still constitute a normal component of the human immune system [178].

TCRγδ T cells constitute a small T‐cell population that accounts for 2–10% of the total CD3^+^ T cells in human peripheral blood and mainly reside in the mucosa and skin [179]. They express semi‐invariant TCRs composed of γ and δ and are involved in a wide range of homeostatic processes in hosts [180]. TCRγδ T cells can recognize lipids and phosphoantigens and are restricted by CD1d. Phosphoantigens are usually natural metabolites, including (E)‐4‐hydroxy‐3‐methyl‐but‐2‐enyl pyrophosphate (HMB‐PP) and isopentenyl pyrophosphate. Unlike MAIT cells, which respond only to riboflavin metabolites or cytokines for activation, the activation of TCRγδ T cells requires a second stimulation signal, which involves the expression of costimulatory molecules such as CD2, CD28, and CD137 [181]. After activation, TCRγδ T cells can exert direct anti‐infective functions by secreting cytotoxic proteins to kill infected cells or secreting proinflammatory cytokines to inhibit the propagation of pathogens, such as influenza virus and *P. falciparum* [182, 183, 184]. Moreover, circulating and resident TCRγδ T cells can also exert indirect anti‐infective activity by improving the anti‐infection capabilities of neighboring immune cells, such as DC cells and macrophages [185]. Furthermore, TCRγδ T cells can act as professional APCs that present antigens from free viral particles and infected cells to other effector immune cells, thereby promoting their antibacterial function [185]. These findings suggest that TCRγδ T cells can utilize various approaches to fight microbial infection. However, the activation of TCRγδ T cells can also exacerbate inflammation, such as that observed during HCV infection [186], or can lead to the generation of excessively activated and exhausted phenotypes, such as those observed during SARS‐CoV‐2 infection [187, 188].

H2–M3‐restricted T cells can also constitute a nonredundant “early” component of the antimicrobial response. H2–M3 is an MHC Class Ib molecule that can present N‐formylated peptides or nonformylated peptides to induce cytotoxic T lymphocyte (CTL)‐mediated cytotoxicity [189], and activated cells are subsequently involved in immunity against pathogens. Primary *L. monocytogenes* infection can induce a rapid response in H2–M3‐restricted T cells, which have cell surface memory markers and longevity similar to those of classically restricted T cells [190].

HLA‐E‐restricted T cells are a minor population of unconventional T cells that recognize antigens in the context of nonpolymorphic MHC‐I molecules. HLA‐E has two different functions: one involves the presence of signal sequence‐derived peptides of other HLA Class I molecules to inhibit NK cell lysis, and the other involves self‐peptides or pathogen peptides derived from antigen‐specific CD8^+^ T cells, which, unlike MAIT cells, lack the ability to produce typical CTL‐associated cytokines and to bind peptides containing canonical amino acids [191]. After presenting pathogen‐derived sequences via HLA‐E, T cells can induce various functions, such as IFN‐γ secretion (*Mtb* and HCV infection), T‐cell proliferation (Epstein Barr virus infection), and cell lysis (*Salmonella* infection) [191].

TCRαβ CD8ααIELs constitute a unique T‐cell population derived from immature thymocytes that express autoreactive TCRs [192]. They are embedded within the intestinal epithelial layer and play significant roles in response to various infections and inflammatory diseases [193]. Additionally, their suppressive and regulatory effects are mediated by IL‐10 [193]. Like CD4^+^ T cells, TCRαβ CD8αα IELs express identical αβTCRs, the expression of which can increase in parallel with a surge in the diversity of the microbial flora [192].

Overall, different types of unconventional T cells can recognize various lipids and peptides, which are presented by diverse semi‐invariant or invariant TCR segments for activation. The activation of unconventional T cells generally relies on a single type of stimulation signal, except for TCRγδ T cells, which require secondary stimulation by costimulatory molecules. Although the functions of other activated unconventional T cells are similar to those of MAIT cells, which primarily kill infected cells and inhibit the propagation or replication of pathogens, some unconventional T cells play immune regulatory and inhibitory roles, such as TCRαβ CD8αα and HLA‐E‐restricted T cells, suggesting that these small cell populations significantly affect host homeostasis.

Above all, MAIT cells have various functions related to microbial infection. First, microbe stimulation induces MAIT cells to secrete various cytokines, such as IFN‐γ, TNF‐α, IL‐17, and IL‐10. IFN‐γ can promote the death of infected cells through autophagy and reduce viral replication in infected hosts [140, 194]. High levels of TNF‐α can induce TNF receptor‐dependent apoptosis in immune cells [195]. The expression of IL‐17 is correlated with chronic inflammatory conditions and disease pathology [196]. Additionally, IL‐10 derived from MAIT cells can promote monocytes to exhibit an anti‐inflammatory phenotype and upregulate the expression of markers such as CD163 [56]. These distinct secreted cytokines can shape the plasticity and primary function of MAIT cells within the microenvironment. Second, microbe stimulation induces the secretion of cytotoxic proteins such as GzmB, perforin, and Gnly by MAIT cells, and an increase in perforin expression in the plasma membrane forms pores to deliver granzymes to induce target cell apoptosis [197], while Gnly induced by microbial exposure can permeabilize cholesterol‐poor microbial membranes and deliver death‐inducing granzymes [198]. Third, activated MAIT cells can secrete chemokines such as CCL3 and CCL20. These chemokines can recruit immune cells and mediate inflammatory responses, such as the recruitment of monocytes to inflammatory sites [21]. Finally, during HIV infection, MAIT cell‐derived cytokines can impede viral entry into CD4^+^ T cells by competitively blocking the CCR5 receptor. These findings suggest that MAIT cells can play a protective role against microbial infection by killing infected cells, promoting inflammatory responses, and inhibiting viral replication [199].

In contrast, MAIT cells can also play pathogenic roles during microbial infection. As powerful polyclonal lymphocyte activators, SAgs can lead to anergy, inflammation, cytotoxicity, and the depletion of T cells in various microbial diseases [151]. The response of MAIT cells to SAgs can be rapidly amplified through the interaction of MHC Class II and the secretion of proinflammatory cytokines, after which MAIT cells are hyperactivated, leading to the development of exhausted and immunosuppressed phenotypes. The robust proinflammatory cytokine response can lead to cytokine storms and life‐threatening effects in hosts, as has been observed in several microbial infections [75, 151, 200]. In addition, SAgs can increase the bacterial burden or viral replication by suppressing factors and modulating the immune response to effectively clear microbes, thereby suggesting another mechanism by which SAgs promote infection [201, 202]. Moreover, chronic infection negatively affects MAIT cells by decreasing the frequency of circulating MAIT cells; upregulating perforin, GzmB, CD69, and PD‐1; inducing intestinal dysbiosis; and decreasing the ability to control translocating bacteria [46]. Long durations of excessive activation, exhaustion, and depletion of MAIT cells, even during treatment, can impair the usefulness of MAIT cells as biomarkers for chronic disease [203]. Therefore, blocking the overactivation of MAIT cells may help restore the number and function of MAIT cells, which in turn improves the antiviral function of MAIT cells and reduces the risk of microbial coinfections. In addition, the number of MAIT cells and the level of cytokine secretion are helpful for predicting disease progression and for providing a basis for selecting appropriate disease control strategies.

## 5 Effects of MAIT Cells on Immune‐Related Diseases

**FIGURE 6:**
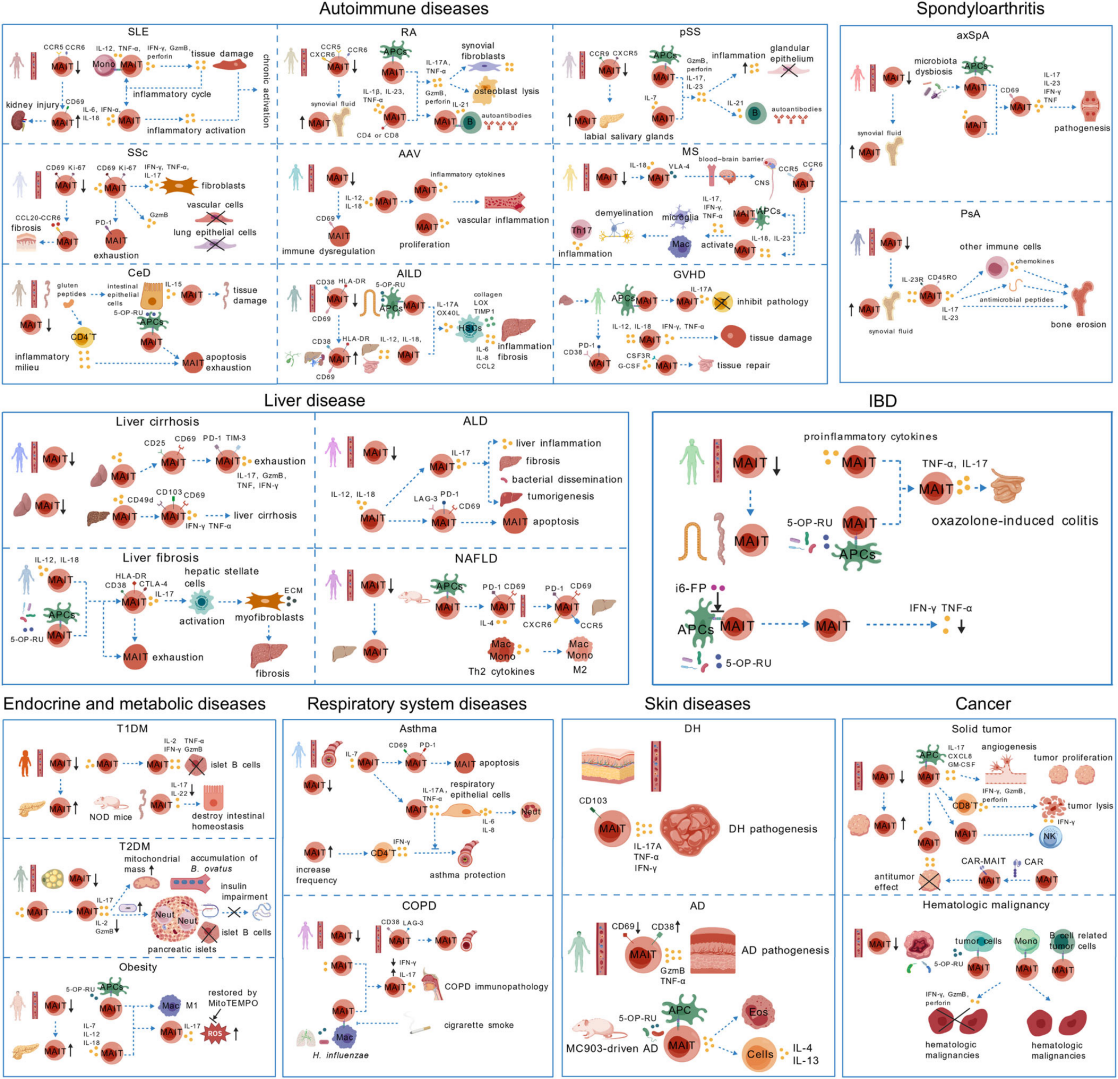
Responses of MAIT cells in immune‐related diseases. In autoimmune diseases, the frequencies of MAIT cells are decreased in blood but increased in tissues. The activated MAIT cells secrete proinflammatory cytokines and cytotoxic factors to promote tissue inflammation, damage, and fibrosis. In spondyloarthritis, a decreased MAIT cell frequency in blood and an increased MAIT cell frequency in tissues such as synovial fluid have been shown, and MAIT cells secrete proinflammatory cytokines and cytotoxic factors to promote pathogenesis, bone erosion, and joint deformity. In the context of liver disease, decreased frequencies of MAIT cells are detected in both blood and liver tissue. Activated MAIT cells secrete proinflammatory cytokines and cytotoxic factors to promote liver inflammation, cirrhosis, and fibrosis. The upregulated expression of PD‐1 and TIM3 can trigger MAIT cell exhaustion. In IBD, decreased frequencies of MAIT cells are detected in both the blood and gut tissue. Activated MAIT cells secrete IL‐17 and TNF‐α to promote colitis. In patients with endocrine and metabolic diseases such as T1DM and obesity, the frequency of MAIT cells in the blood is decreased but that in the pancreas is increased. Activated MAIT cells secrete proinflammatory cytokines and cytotoxic factors to damage islet B cells within the pancreas or induce the production of high levels of ROS. In the context of respiratory system diseases, MAIT cells can exacerbate asthma and COPD immunopathology through the secretion of IFN‐γ and IL‐17. In skin diseases, MAIT cells can secrete proinflammatory cytokines and cytotoxic factors to promote the pathogenesis of DH and AD. In cancer patients, MAIT cells have dual effects: they can secrete IFN‐γ and cytotoxic factors to lyse tumor cells or secrete IL‐17, CXCL8, and GM‐CSF to promote angiogenesis and tumor proliferation. *Abbreviations*: SLE, systemic lupus erythematosus; RA, rheumatoid arthritis; pSS, primary Sjögren's syndrome; SSc, systemic sclerosis; AAV, antineutrophil cytoplasmic autoantibody (ANCA)‐associated vasculitis; MS, multiple sclerosis; CeD, celiac disease; AILD, autoimmune liver disease; GVHD, graft‐versus‐host disease; axSpA, axial spondyloarthritis; PsA,; psoriatic arthritis; ALD, alcohol‐associated liver disease; NAFLD, nonalcoholic fatty liver disease; T1DM, Type 1 diabetes mellitus; T2DM, Type 2 diabetes mellitus; COPD, chronic obstructive pulmonary disease; DH, dermatitis herpetiformis; AD, atopic dermatitis; HSCs, hepatic stellate cells; IBD, inflammatory bowel disease; GM‐CSF, granulocyte‒macrophage colony stimulating factor; Mono, monocyte; B, B cell; Mac, macrophage; APC, antigen‐presenting cell; HSC, hepatic stellate cells; Neut, neutrophil.

Immune‐related diseases are a common, clinically diverse group of conditions that share similar pathogenetic characteristics of immune signaling pathways but also present unique characteristics [204]. They include autoimmune diseases, spondyloarthritis (SpA), liver disease, inflammatory bowel disease (IBD), and endocrine and metabolic diseases. In many diseases, the number of MAIT cells is decreased in the blood but increased in tissues. Activated MAIT cells can promote inflammatory responses, tissue damage, and autoantibody production through the secretion of cytotoxic molecules. In liver diseases, MAIT cells can drive inflammation, fibrosis, and anti‐infective effects. In metabolic and respiratory diseases, they contribute to the secretion of IL‐17 and IFN‐γ (Figure 6). The following sections describe the functions and effects of MAIT cells in various immune‐related diseases.

### 5.1 Autoimmune Diseases

Autoimmune diseases are a group of chronic, multisystem disorders caused by immune system dysfunction. Common examples include systemic lupus erythematosus (SLE), rheumatoid arthritis (RA), primary Sjögren's syndrome (pSS), and systemic sclerosis (SSc). The core pathology of these diseases involves chronic, systemic inflammation driven by aberrant immune responses, which can lead to irreversible organ damage and contribute to high rates of morbidity, disability, and mortality [205]. Although their pathogenesis is not fully understood, various immune cells, particularly T cells, are known to be central to their development [206, 207, 208, 209]. Accumulating evidence highlights the important role of MAIT cells in these disorders, identifying them as potential therapeutic targets [210]. The following sections review the activation, phenotype, distribution, function, and role of MAIT cells in autoimmune diseases.

#### 5.1.1 Systemic Lupus Erythematosus

SLE is a systemic autoimmune disease characterized by the production of autoantibodies against nuclear antigens, affecting multiple organs, including the skin and kidneys [211]. Recent studies have revealed pathological dynamic changes in peripheral blood MAIT cells during SLE flares.

Research has indicated that the frequency and absolute number of MAIT cells in the peripheral blood of patients with SLE are significantly lower than those in healthy controls [212]. Further analysis revealed increased surface expression of apoptosis‐related markers (Fas, Caspase‐3, and 7‐AAD) on these cells, suggesting a close association with activation‐induced cell death. While the number of MAIT cells is correlated with age and lymphocyte count, the frequency of these cells decreases as the expression of the activation marker CD69—which is positively correlated with the SLE disease activity index—increases [213], indicating the potential of these cells as biomarkers of disease activity.

The chronic activation of MAIT cells in SLE may occur via two mechanisms. First, monocytes in SLE patients exhibit increased MR1 antigen‐presenting capacity and secrete substantial amounts of IL‐12 and TNF‐α. These cytokines promote the production of IFN‐γ, GzmB, and perforin by MAIT cells, creating a self‐amplifying inflammatory cycle that mediates tissue damage [210]. Second, in the absence of exogenous antigens, inflammatory cytokines such as IL‐6, IL‐18, and IFN‐α can directly activate MAIT cells. This mechanism implies that systemic inflammation during active SLE is a key upstream driver of their chronic activation [213]. Notably, plasma IL‐18 levels are negatively correlated with peripheral MAIT cell frequency, further suggesting that inflammatory activation accelerates their depletion.

Despite their activated phenotype, MAIT cells exhibit impaired effector functions, including significantly reduced IFN‐γ secretion, which has been largely attributed to inherent defects in the Ca^2^^+^/calmodulin/NFAT1 signaling pathway. Furthermore, dysfunction of NKT cells may exacerbate MAIT cell impairment via intercellular networks [212].

Peripheral blood MAIT cells from SLE patients also highly express the chemokine receptors CCR5 and CCR6 [214], which favor their migration and enrichment in inflammatory tissues such as the intestine and the liver. In the FcγRIIb^−/−^ Yaa lupus mouse model, the proportion of MAIT cells expressing IL‐17 or IFN‐γ in the spleen and kidney is increased [215]. These cells can interact with B cells via TCR–MR1 or CD40L–CD40 costimulation pathways, promoting autoantibody production. Consistently, MR1^−/−^ lupus mice, which are deficient in MAIT cells, exhibit reduced disease severity, including decreased autoantibody production, lower glomerulonephritis scores, and attenuated germinal center and T‐cell responses [215].

In summary, MAIT cells contribute to SLE progression through multiple mechanisms including enhanced antigen presentation, inflammatory microenvironmental drivers, signaling pathway defects, and cellular network dysregulation. Therefore, targeting MAIT cells or their key regulatory nodes (e.g., MR1, IL‐12, and NFAT1) represents a potential therapeutic strategy for SLE.

#### 5.1.2 Rheumatoid Arthritis

RA is a systemic inflammatory disease characterized by chronic synovitis and the formation of pannus. This pathological process drives the progressive destruction of bone and cartilage, ultimately resulting in joint deformity and loss of function [216, 217]. Evidence suggests that MAIT cells drive joint inflammation and bone destruction via a pathological sequence.

Clinically, the MAIT cell frequency and absolute number are reduced in the peripheral blood of RA patients, which is correlated with disease activity [218]. MAIT cells express high level of chemokine receptors (e.g., CCR5, CCR6, and CXCR6), which recognize ligands such as E‐selectin and CCL20 in the inflammatory joint microenvironment, facilitating their migration from the circulation [212, 219]. Within joints, MAIT cell activation is driven not only by the classical MR1‐dependent TCR recognition of microbial metabolites but also, more critically, by cytokines such as IL‐1β, IL‐23, and TNF‐α [220]. This non‐TCR‐dependent abnormal activation not only induces a phenotypic shift in MAIT cells from CD8^+^ to CD4^+^, along with the downregulation of CD161 and the formation of a dysfunctional CD161^−^ subset [218, 221], but also directly contributes to persistent inflammation and tissue damage [222].

Activated MAIT cells exhibit a strong proinflammatory profile and secrete large amounts of IL‐17A and TNF‐α [223]. Furthermore, MAIT cells can interacting with B cells via CD40L–CD40 signaling and IL‐21 to promote autoantibody production and amplify systemic autoimmunity [224]. Animal studies have confirmed that the severity of collagen‐induced arthritis is reduced in MR1^−^/^−^ mice lacking MAIT cells, whereas reinfusion of MAIT cells restores disease activity [220].

In summary, in the context of RA, MAIT cells are recruited to joints via the inflammatory microenvironment, undergo abnormal cytokine‐driven activation, and differentiate into proinflammatory effector cells. Through multiple mechanisms, including the secretion of key inflammatory factors, and assisting autoantibody production, they collectively drive persistent synovitis, cartilage destruction, and bone erosion, thereby playing key pathogenic roles in both local and systemic RA immunopathology.

#### 5.1.3 Primary Sjögren's Syndrome

pSS is an autoimmune disorder characterized by lymphocyte infiltration leading to exocrine gland damage [225]. Additionally, pSS is frequently associated with a range of extraglandular or systemic complications, such as renal tubular acidosis, cryoglobulinemia, thrombocytopenia, hypergammaglobulinemia, and lymphoma [226, 227]. In pSS, MAIT cells contribute to glandular immunopathology owing to alterations in their distribution, phenotype, and function.

Studies have shown that the MAIT cell frequency and absolute number are significantly reduced in the peripheral blood of pSS patients, whereas notable MAIT cell infiltration is detected in target tissues such as labial glands—a finding that is uncommon in healthy controls and nonpSS sialadenitis patients [225]. This tissue‐specific accumulation is associated with high MAIT cell expressions of the homing receptors CCR5, CCR6, CCR9, and CXCR6 can selectively mediate MAIT cell migration and infiltration [228].

Peripheral blood MAIT cells in pSS patients display an immature or hyporesponsive phenotype, marked by an increased proportion of CD45RA^+^CCR7^+^ cells and a relative increase in CD4^+^ subsets, whereas their capacity to secrete activation markers (e.g., CD69 and CD154) and certain proinflammatory cytokines (e.g., TNF and IFN‐γ) is reduced [225]. Once MAIT cells migrate to the salivary glands, they can be polarized into IL‐17‐secreting effector cells in an IL‐7/IL‐23‐rich inflammatory microenvironment. This polarization occurs via the non‑TCR‑dependent activation of the STAT3/HIF‑1α pathway and upregulation of the key transcription factor RORγt [229]. Furthermore, in CD8^+^ MAIT cells in the context of pSS, the downregulation of IL‑7R, which is associated with lymphocyte focus scores and B‐cell hyperactivation, can be triggered by two distinct signals: first, ligand‑induced feedback resulting from elevated IL‑7 levels [230, 231, 232], and second, TCR‑mediated activation itself [233]. Thus, reduced IL‑7R expression serves as a functional marker, indicating that in patients with pSS, MAIT cells receive stimulatory signals via either IL‑7‐dependent or TCR‑dependent pathways [234].

Activated MAIT cells contribute to pSS pathogenesis through several mechanisms. First, they secrete substantial amounts of IL‐17, exacerbating local inflammation, recruiting immune cells, and damaging the glandular epithelium [225, 229]. Second, MAIT cells derived predominantly from the CD8^+^CD4^−^ and DN subsets express high levels of TNF‐α, IFN‐γ, and GzmB, which can directly kill glandular epithelial cells via the perforin/granzyme pathway [235]. Third, they produce IL‐21, which facilitates B‐cell activation and promotes autoantibody production (e.g., anti‐SS‐A52 antibodies) [234]. Additionally, DN‐derived MAIT cells correlate positively with complement C4 levels, suggesting a potential role in complement activation and inflammatory amplification [236].

Clinically, the reduction in the number of peripheral blood MAIT cells is negatively correlated with disease activity indicators such as C‐reactive protein levels, erythrocyte sedimentation rates, autoantibody levels, and IgG levels [237], indicating the potential of these cells as disease activity biomarkers. Notably, disease‐modifying antirheumatic drugs such as leflunomide and hydroxychloroquine can modulate MAIT cell functions (e.g., IL‐21 production), offering clues for future therapeutic targeting.

In summary, in the context of pSS, MAIT cells migrate from the blood to inflamed glands, where local cytokines activate them and induce their polarization into IL‐17‐secreting effectors. By releasing proinflammatory factors, exerting direct cytotoxicity, and assisting autoantibody production, MAIT cells collectively drive glandular autoimmune inflammation and tissue destruction, making them key players that bridge innate and adaptive immunity and mediate tissue damage in pSS.

#### 5.1.4 Systemic Sclerosis

SSc is characterized by immune dysregulation, vascular dysfunction, and progressive fibrosis [238] and currently has a poor prognosis and limited effective treatment options [239]. In individuals with SSc, MAIT cells exhibit markedly abnormal distribution and altered activation states, contributing to disease progression, with a particularly crucial role in lung involvement [240].

Studies have shown that the frequency and absolute number of MAIT cells in the peripheral blood of patients with SSc are significantly reduced [241]. Moreover, greater disease severity is generally associated with a more pronounced reduction in MAIT cells, although a definitive statistical correlation remains to be established [240]. Despite this decrease, circulating MAIT cells are highly activated in SSc patients, as indicated by an increasing trend in surface CD69 and Ki‐67 expression [240]. The specific pathways driving this activation remain incompletely defined. The current understanding posits that the chronic inflammatory microenvironment in SSc provides persistent stimulation, potentially driving MAIT cells into a state of “functional adaptation.” It is thus hypothesized that in SSc patients, MAIT cells are more likely to be activated via cytokine‐mediated pathways [228, 242]. Recent evidence suggests that the loss of peripheral MAIT cells is likely not due to apoptosis but to their active recruitment to fibrotic target organs via the CCL20–CCR6 axis [240]. A plausible model is that inflamed lungs and skin, which highly express CCL20, attract CCR6‐expressing MAIT cells from the circulation.

In lung or skin tissues, activated (CD69^+^) MAIT cells may drive fibrosis through two main pathways: by producing proinflammatory/profibrotic cytokines (e.g., IFN‐γ, TNF‐α, and IL‐17) that stimulate fibroblast or by releasing cytotoxic molecules such as GzmB to directly injure lung epithelial or vascular cells, thereby triggering aberrant repair responses [240]. Further transcriptomic analyses revealed that SSc MAIT cells display an activated phenotype coupled with upregulated expression of the inhibitory molecule PD‐1, suggesting that chronic stimulation may induce a state of functional exhaustion or adaptation that contributes to pathogenesis [242].

In summary, MAIT cells have been implicated in SSc pathogenesis, particularly in SSc‐ILD. However, their precise functions within target tissues, the specific mechanisms by which they regulate fibrosis, and their utility as biomarkers for disease activity or prognosis require further investigation through direct study of patient tissues.

#### 5.1.5 ANCA‐Associated Vasculitis

Antineutrophil cytoplasmic autoantibody (ANCA)‐associated vasculitis (AAV) is an autoimmune disease characterized by necrotizing inflammation of small blood vessels and the presence of serum ANCAs [224]. It frequently involves the kidneys and lungs and manifests as immune‐mediated glomerulonephritis and alveolar capillaritis, respectively [243]. In AAV patients, MAIT cells undergo distinct numerical, phenotypic, and functional changes that may be linked to disease activity and potential relapse [244].

A key feature in AAV patients, particularly in those with granulomatosis with polyangiitis (GPA) and microscopic polyangiitis (MPA) subtypes [245], is a significantly reduced frequency of circulating MAIT cells [246]. This reduction persists even in remission, suggesting that it reflects underlying immune dysregulation. Circulating MAIT cells in AAV patients show evidence of persistent activation, with elevated surface CD69 expression during both the active phase and the remission phase. These findings indicate that MAIT cell activation is maintained by the chronic inflammatory environment rather than by acute antigen encounter, suggesting that these cells are skewed toward a chronic state of immune activation that does not rely on classical TCR‐dependent pathways [244]. Consistent with this mechanism, the cytokine‐rich microenvironment (e.g., IL‐12 and IL‐18) within giant cell arteritis lesions reprograms MAIT cells into effector cells with a proinflammatory phenotype and increases their proliferative capacity. This drives their participation in vascular inflammation, suggesting that direct activation via cytokines contribute to the inflammatory response [247]. Functionally, MAIT cells in AAV patients display a bias toward a Th1‐type response, resulting in increased IFN‐γ production upon stimulation. These findings align with the predominant Th1 immune environment observed in patients with AAV, particularly those with GPA, suggesting that MAIT‐derived inflammatory cytokines could exacerbate vascular inflammation [244].

Although the reduced MAIT cell frequency could be a nonspecific consequence of chronic inflammation, the persistently low frequency of these cells and their activated, Th1‐biased phenotype during remission may hold disease‐specific significance, potentially serving as a cellular indicator of relapse risk or ongoing immune dysregulation [246]. Future studies should clarify the specific localization, local functions, and interactions of MAIT cells with ANCAs within AAV vascular lesions (e.g., the kidney and lungs) to define their precise role in disease pathogenesis.

#### 5.1.6 Multiple Sclerosis

MS is an autoimmune disease that causes inflammatory demyelination in the central nervous system (CNS) [248], its main pathological features are include inflammatory cell infiltration, neuronal degeneration, axonal injury, and reactive glioma [228]. MAIT cells, whose distribution, activation, and function are altered, are key players in bridging peripheral immunity and CNS inflammation [249]. However, whether their net effect is predominantly proinflammatory or regulatory remains debated.

A consistent finding is that the peripheral blood MAIT cell frequency is significantly reduced in MS patients, particularly during active or relapsing phases, but can recover following clinical remission or treatment, indicating a dynamic correlation with disease activity [250]. Conversely, in MS patients, MAIT cells infiltrate the cerebrospinal fluid and active demyelinating lesions [251]. This migration involves several key steps. First, elevated levels of serum IL‐18 provide a TCR‐independent activation signal [252]. Activated MAIT cells subsequently upregulate the expression of the key integrin very late antigen‐4 (VLA‐4), which is critical for crossing the blood‒brain barrier [252]. Finally, MAIT cells highly express chemokine receptors (e.g., CCR6 and CCR5) creating an ability that guides their migration [253].

Within the CNS, MAIT cells can be further activated via the classical MR1–TCR pathway [253] and cytokine‐driven (e.g., IL‐18 and IL‐23) [250]. Their activation drives their differentiation into effector cells that express RORγt and produce a plethora of proinflammatory cytokines (e.g., IL‐17, IFN‐γ, and TNF‐α) [254]. These cytokines can activate microglia/macrophages, disrupt the blood‒brain barrier, and contribute directly or indirectly to oligodendrocyte damage and demyelination. They may also cooperate with other immune cells, such as Th17 cells, to amplify local inflammation.

Despite some evidence for a contact‐dependent regulatory function (e.g., inhibition of IFN‐γ production by pathogenic Th1 cells), which may be impaired in MS patients [250], the predominant evidence supports a proinflammatory role. This functional ambiguity may depend on disease stage, activation signal strength, and the local microenvironment.

Clinically, the dynamics of circulating MAIT cells correlate with disease activity and MS lesions, and effective treatment can deplete proinflammatory, IL‐17‐secreting MAIT cell subsets [250]. Furthermore, intestinal dysbiosis may influence MS pathogenesis by shaping MAIT cell functional polarization, providing a cellular immunology perspective on the gut‒brain axis [228].

In summary, in the context of MS, MAIT cells serve as a bridge between innate and adaptive immunity and potentially connect the gut microbiota with CNS inflammation, making these cells intriguing therapeutic targets.

#### 5.1.7 Celiac Disease

Celiac disease (CeD) is an organ‐specific autoimmune disorder triggered by the ingestion of gluten and related proteins, and is characterized by immune‐mediated pathology in the small intestine [255], which easily leads to intestinal diseases associated with bloating, diarrhea, or malabsorption symptoms [256]. MAIT cells display a distinctive “peripheral‒intestinal synchronous depletion” pattern, which may contribute to intestinal barrier dysfunction and local immune dysregulation.

Compared with healthy individuals, adult and pediatric CeD patients exhibit significant and synchronous decreases in the frequency of MAIT cells in both the peripheral blood and the small intestinal lamina propria, suggesting systemic disturbance rather than simple local redistribution [257]. The depletion of MAIT cells in CeD patients likely involves multiple mechanisms. One pathway is initiated by gluten peptides, which induce intestinal epithelial cells to express the cytokine IL‐15 and upregulate stress ligands such as HLA‐E and MICA/B. IL‐15 can mediate cytotoxicity and directly contribute to tissue damage by activating unconventional T cells, including MAIT cells and γδ T cells [256]. Furthermore, the gluten‐induced impairment of the intestinal epithelial barrier increases its permeability, facilitating the translocation of bacterial metabolites (e.g., 5‐OP‐RU). This leads to chronic MR1–TCR‐dependent activation of bacterial metabolite‐sensitive MAIT cells. Such persistent stimulation, similar to that observed in other chronic immune activation states, such as HIV infection, tends to drive apoptosis or functional exhaustion rather than proliferation [128, 257]. Finally, the inflammatory milieu in individuals with CeD is dominated by a strong adaptive immune response driven by gluten‐reactive CD4^+^ T cells, which alter the cytokine landscape [258] and may further compromise MAIT cell survival or recruitment.

Consequently, MAIT cell deficiency in CeD patients may impair innate immune surveillance [257] and disrupt regulatory networks, allowing proinflammatory responses (e.g., γδ T‐cell‐mediated IFN‐γ production) to prevail and indirectly exacerbate tissue damage [256].

Notably, the extent of the decrease in the number of MAIT cells correlates with intestinal inflammation. However, even when a gluten‐free diet improves symptoms [256], it often fails to normalize MAIT cell numbers, suggesting that these abnormalities may constitute a persistent immune imprint.

In summary, systemic MAIT cell depletion likely weakens intestinal defense in CeD patients, and their dysregulation may be linked to microbiota disruption and inflammation. Targeting MAIT cell homeostasis or its interplay with gluten‐specific immunity could inform novel interventions, although the precise mechanisms require further elucidation.

#### 5.1.8 Autoimmune Liver Disease

Autoimmune liver disease (AILD) encompasses autoimmune hepatitis (AIH), primary biliary cholangitis (PBC), and primary sclerosing cholangitis (PSC) [259]. AIH primarily targets hepatocytes but can also affect bile ducts [260]. PBC is characterized by specific destruction of interlobular bile ducts, leading to reduced bile secretion and accumulation of toxic substances within the liver tissues [261]. PSC predominantly affects large‐ and medium‐sized intrahepatic and extrahepatic bile ducts, causing concentric, obliterative fibrosis, and multifocal bile duct strictures [262]. Despite their differing primary targets, all three diseases share a common pathological character: unchecked inflammation drives progressive liver fibrosis, which can ultimately culminate in cirrhosis [259]. Under these conditions, MAIT cells, which are constitutively enriched in the healthy liver, comprising 20–50% of intrahepatic T cells [228, 263], undergo profound numerical, phenotypic, and functional alterations. These changes are closely linked to disease‐related inflammation and fibrosis [254].

A hallmark of AILD is a significant and persistent reduction in peripheral blood MAIT cell frequency [254], which often fails to normalize even after successful standard therapy (e.g., ursodeoxycholic acid), suggesting enduring immune dysregulation [264]. In contrast, MAIT cells are abnormally enriched in hepatic portal tracts and sites of bile duct injury [265]. This altered distribution is likely mediated by interactions between chemokines (e.g., CCL20) secreted by diseased bile duct epithelial cells and immune cells and corresponding homing receptors (e.g., CCR6, CXCR6, and α4β7 integrin) on MAIT cells [266].

Circulating and intrahepatic MAIT cells exist in a state of simultaneous chronic activation and functional exhaustion. They display high surface levels of activation markers (e.g., CD38, HLA‐DR, and CD69) [23, 266] but have impaired proliferative capacity and reduced production of effector molecules such as IFN‐γ and GzmB. This exhausted phenotype is further characterized by upregulated expression of inhibitory receptors (e.g., PD‐1 and CTLA‐4) [23, 267]. This activation is driven by two main pathways: first, bacterial metabolite translocation due to gut barrier dysfunction can activate MAIT cells via the MR1–TCR pathway [222]; and second, high levels of cytokines (e.g., IL‐12, IL‐18) in the liver and serum of individuals with AILD drive MAIT cell activation and IL‐17A production [23, 228].

Activated MAIT cells are key sources of IL‐17A and CD40L. These molecules directly activate hepatic stellate cells (HSCs), inducing the secretion of profibrotic (e.g., collagen, LOX, and TIMP‐1) and proinflammatory mediators (e.g., IL‐6, IL‐8, and CCL2), thereby driving liver inflammation and fibrosis. The frequency of activated MAIT cells is positively correlated with fibrosis severity [23].

In summary, in AILDs, MAIT cells transition from defensive immune residents to pathogenic drivers. Their dysregulation is correlated with disease activity, making them not only potential biomarkers for assessing liver fibrosis progression but also novel therapeutic targets (e.g., the IL‐17 pathway or upstream activation nodes) for intervention in AILD.

#### 5.1.9 Graft‐Versus‐Host Disease

Graft‐versus‐host disease (GVHD) is a serious complication following transplantation, in which donor‐derived effector T cells become activated and expand. These T cells subsequently recognize and attack the recipient's normal nonhematopoietic tissues, including the intestine, lungs, liver, and skin. The activation and expansion of these T cells trigger the secretion of many proinflammatory cytokines and the recruitment of more inflammatory effector cells to the target organs, resulting in a cascade amplification effect that ultimately leads to tissue damage [268]. MAIT cells play complex and crucial dual roles in GVHD because of their mucosal homing properties, rapid microbial responsiveness, and immunoregulatory potential [269]. They are particularly involved in the pathology of target organs such as the intestine and skin [270].

The functional impact of MAIT cells in GVHD is highly context dependent and influenced by their origin (host vs. donor), mode of activation, and local microenvironment. Host MAIT cells that survive preconditioning, particularly in the colon, can be activated via the MR1/TCR‐dependent pathway to become MAIT17 cells. These cells secrete IL‐17A to help regulate the local microbiota, inhibit the expansion of pathogenic donor T cells [271], and promote tissue repair [272]. Within the donor graft, granulocyte‐colony stimulating factor (G‐CSF) can promote the immunosuppressive and tissue‐repair functions of specific MAIT cell subsets (such as G‐MAIT) by binding to CSF3 receptor (CSF3R). These G‐MAIT cells then home to the intestine via the CXCR6‒CXCL16 axis to exert protective effects [269]. However, when strongly activated by inflammatory cytokines (e.g., IL‐12/IL‐18), MAIT cells can produce large amounts of cytotoxic cytokines such as IFN‐γ and TNF‐α. While this may contribute to the beneficial graft‐versus‐leukemiaeffect, it can also exacerbate tissue damage [273]. Adoptive transfer studies in preclinical xenogeneic GVHD models have demonstrated the therapeutic potential of MAIT cells, and show that adoptive transfer of third‐party donor MAIT cells can prevent or alleviate xenogeneic GVHD by inhibiting host‐reactive T‐cell proliferation, activation, and tissue infiltration [274].

The impact of MAIT cells on GVHD outcomes has not been fully elucidated, with studies reporting divergent findings. In animal models, reduced GVHD severity is correlated with increased intestinal MAIT cell infiltration. These findings suggest that their preferential migration and accumulation in the gut may be associated with a lower incidence of acute GVHD (aGVHD), particularly intestinal aGVHD [269]. Conversely, some studies have shown that a higher frequency of MAIT cells in the graft correlates with an elevated risk of aGVHD [275, 276]. This apparent paradox may be explained by activated MAIT cells can also secrete proinflammatory cytokines (e.g., IFN‐γ and TNF‐α) that can exacerbate tissue damage, depending on the microenvironment.

After transplantation, MAIT cell reconstitution in peripheral blood is often delayed, with cells displaying an activated and exhausted phenotype (e.g., CD38^+^, PD‐1^+^, TIM‐3^+^), great MAIT cell frequencies are linked to chronic GVHD development [276, 277], whereas early recovery has shown a complex relationship with infection risk and long‐term survival [275].

In summary, MAIT cells play a multifaceted role in GVHD through mechanisms shaped by their source, activation pathway, and interaction with the microbiota. Strategies to modulate their function, such as adoptive transfer or microbiota‐directed interventions, represent promising therapeutic approaches.

### 5.2 Spondyloarthritis

Spondyloarthritis (SpA), an inflammatory rheumatic disease, is classified into axial SpA (axSpA) and peripheral SpA subtypes [278]. Among these, ankylosing spondylitis (AS), and psoriatic arthritis (PsA), represent the most common forms of the disease [278]. Clinically, SpA manifests with musculoskeletal features (synovitis, sacroiliitis, spinal inflammation, mucositis, and dactylitis), affects axial bones, peripheral joints, and organs, and affects extra‐articular sites [279, 280]. MAIT cells are abundant in mucosal tissues, and activated MAIT cells are abundant in individuals with SpA and PsA, where they induce Th17 cytokines. Furthermore, both the number and function of circulating MAIT cells are altered in the contexts of SpA, and PsA [281]. The following sections discuss the changes in MAIT cell levels and functions in axSpA and PsA to explore their role in SpA.

#### 5.2.1 Axial SpA

axSpA, which primarily affects the axial spine [278], is a group of rheumatic immune diseases characterized by chronic inflammation of the sacroiliac joints and spinal insertions. It can lead to bone erosion, new bone formation, and spinal fusion, resulting in structural damage and functional impairment, threatening human health [279, 282]. In addition to their involvement in the innate and adaptive immune responses in axSpA pathogenesis, TNF‐α and IL‐17 (IL‐17A) have been identified as two major cytokines implicated in inflammatory responses and axSpA joint lesions [279, 283, 284]. IL‐17 increases the expression of LL37, an antimicrobial peptide and a psoriasis autoantigen that promotes the production of proinflammatory factor and exacerbates inflammation [285, 286]. IL‐23 is a key activating driver of MAIT cells, the IL‐23/IL‐17 axis has been recognized as another major pathway involved in the inflammatory response in the context of axSpA, which contributes to the pathogenesis of the disease [287, 288]. Studies have revealed that peripheral blood MAIT cells in axSpA patients are one of the most abundant IL‐17A‐producing cell populations. Following TCR‐dependent recognition of microbial antigens, MAIT cells perform effector functions by secreting proinflammatory cytokines (e.g., IL‐17) and producing cytotoxic molecules [24, 284], suggesting that MAIT cells may play a significant role in the pathological environment of axSpA. Clinical studies have revealed reduced MAIT cell counts in the blood of axSpA patients compared with those in healthy controls [279], whereas the synovial compartments are enriched in this cell subset [289]. These findings indicate the presence of MAIT cells within the synovial tissue of axSpA patients [283]. Activated MAIT cells express increased levels of the markers CD69 and produce IFN‐γ, TNF, IL‐17, and IL‐22 [289, 290]. These findings suggest that MAIT cells may contribute to axSpA pathogenesis via the IL‐23/IL‐17 axis.

Individuals with axSpA diseases, including their prototypical representative AS, have been shown to exhibit lower MAIT cell frequencies in peripheral blood than healthy controls do [290], indicating that MAIT cells may migrate to inflammatory sites and participate in disease progression in axSpA patients. Activated MAIT cells efficiently produce large amounts of inflammatory cytokines, whereas IL‐2‐, TNF‐α‐, and IFN‐γ‐producing MAIT cell populations do not significantly differ between AS patients and HCs. Compared with healthy individuals, AS patients exhibit a greater proportion of IL‐17‐producing MAIT cells [290]. Additionally, researchers have reported elevated IL‐12 levels in AS patients [291] along with gut microbiota dysbiosis [292]. These findings indicate that MAIT cells can be activated through bacterial riboflavin metabolite pathways and the IL‐12/IL‐18 cytokine pathway, continuously driving IL‐17 inflammatory responses within the “gut–joint axis” [290]. Therefore, MAIT cells participate in the pathogenesis of AS by robustly producing inflammatory cytokines, transforming from “bystanders” to core effector cells in axSpA/AS pathogenesis. Their altered phenotype and function are closely associated with disease activity, proinflammatory responses, and structural damage, making them potential therapeutic targets.

#### 5.2.2 Psoriatic Arthritis

PsA is a chronic inflammatory disease within the SpA family. PsA frequently occurs in the context of cutaneous psoriasis and commonly presents with features similar to juvenile idiopathic arthritis, with axial skeletal involvement alongside extra‐articular manifestations, including dactylitis, arthritis, and uveitis [293, 294, 295]. The primary cytokine involved in PsA pathogenesis is IL‐23, whereas Th17 cells that release IL‐17 also contribute to disease development [296], indicating that the IL‐23/IL‐17 axis is a core pathway involved in PsA pathogenesis. The cytokines IL‐23 and IL‐17 play crucial roles in chronic synovial inflammation and contribute significantly to skin lesions in PsA patients [296]. IL‐17 is highly expressed in the joints and skin, where it drives bone erosion by inducing antimicrobial peptides, chemokines, and proinflammatory factors, thereby amplifying inflammation and promoting bone destruction in both psoriatic tissues and tissues affected by PsA [297].

Studies have revealed that the frequency of MAIT cells is low in peripheral blood but relatively high in joint synovial fluid, indicating that these cells accumulate at the local pathological site—the joints. These cells predominantly present a CD8^+^ memory phenotype, with fewer CD4^+^ MAIT cells, while the remainder are CD4^−^CD8^−^. In PsA patients, most MAIT cells in synovial fluid exhibit a memory phenotype (CD45RO^+^) [286]. These synovial MAIT cells highly express functionally active IL‐23R and exhibit significant proliferation and produce large amounts of IL‐17A upon IL‐23 stimulation without requiring MR1–TCR pathway activation [224, 298]. These cells act as key effector cells in the IL‐23/IL‐17 axis.

In summary, MAIT cells in synovial fluid are a major source of IL‐17A and constitute part of the IL‐23/IL‐17 cytokine network, playing a crucial role in the pathogenesis of PsA.

### 5.3 Liver Disease

The liver is a vital immune organ that maintains homeostasis within the body. When the body is affected by bacterial or viral infection or other environmental factors, this equilibrium is disrupted, triggering a series of liver diseases. These diseases include alcohol‐associated liver disease (ALD), liver fibrosis, nonalcoholic fatty liver disease (NAFLD), AILD, and viral hepatitis [299, 300]. MAIT cells are abundant in the liver; in addition to secreting cytokines and cytotoxic effector molecules for host defense, MAIT cells exhibit tissue healing phenotypes and interact with parenchymal, myeloid, and stromal cells to induce fibrosis and promote wound repair [300]. The numbers and functions of MAIT cell populations change in the context of most liver diseases [300, 301]. This section explores the role of MAIT cells in these diseases below by elucidating the changes in MAIT cell frequency and function across various hepatic pathologies.

#### 5.3.1 Liver Cirrhosis

Liver cirrhosis is a serious, multisystem disease caused mainly by alcohol consumption [302]. Studies have indicated that in patients with cirrhosis, the MAIT cell counts in the blood and liver are significantly reduced [303, 304]. The vast majority of MAIT cells are CD8^+^ (nearly 80%) or DN (the remaining CD8^−^CD4^−^ up to 20%). In the blood of patients with cirrhosis, compared with that in healthy controls, MAIT cells exhibit an activated phenotype, with increased expression of CD25 and CD69, which is negatively correlated with the MAIT cell frequency and increased expression of IL‐17, GzmB, IFN‐γ, and TNF in liver tissue. MAIT‐cell numbers in the liver are greater than those in blood and exhibit elevated PD‐1 and TIM‐3 expression, indicating significantly increased MAIT cell exhaustion and increased IL‐17 expression; however, GzmB, IFN, and TNF levels are not significantly altered [22].

Patients with terminal cirrhosis complicated by ascites (decompensated cirrhosis) exhibit increased susceptibility to infection. MAIT cells are severely depleted in blood and liver tissue and are characterized by immune dysfunction, whereas peritoneal MAIT cells remain abundant, activated, and functionally intact [303]. The levels of multiple receptors of proinflammatory cytokines and chemokines are elevated, including markers such as CD69, as well as CCR6, CCR5, CXCR3, and CD25, which is accompanied by increased production of IL‐17 and GzmB [22, 304]. Compared with healthy MAIT cells, peritoneal MAIT cells respond to bacterial products, indicating that despite severely reduced MAIT cell numbers with impaired phenotypes in the circulation, functional MAIT cells in patients with decompensated cirrhosis preferentially migrate toward sites of infection, such as the peritoneal cavity. These cells exhibit an activated tissue‐resident phenotype characterized by CD69, CD103, and CD49a expression and maintain the effective expression of TNF‐α and IFN‐γ in an MR1–TCR‐independent manner to exert their anti‐infective effects [303, 304].

In summary, compared with those in the blood and liver of patients with cirrhosis, peritoneal MAIT cells exhibit relatively intact functions, highlighting the critical role of this immune cell subset in the context of severe cirrhosis. Targeting MAIT cells via costimulatory pathways for immunotherapy can increase their expansion and thereby improve infection control.

#### 5.3.2 Liver Fibrosis

Liver fibrosis is a common response to chronic liver injury, characterized by scarring and arising from prolonged parenchymal cell damage or inflammation. The fibrogenic response is characterized by the progressive accumulation of ECM components rich in fibrillar collagen and impaired matrix turnover. This process is driven by a heterogeneous population of hepatic myofibroblasts that are derived primarily from HSCs and portal fibroblasts [302]. In the context of chronic liver injury, MAIT cells are directly activated by IL‐12 and IL‐18 [23, 305], and MAIT cells modulate fibrosis progression through changes in cytokine expression and function. Substantial evidence indicates that MAIT cells promote liver fibrosis in individuals with chronic liver injury [22, 306].

Using AILD as an example, we illustrate the specific role of MAIT cells in chronic liver injury and the changes in their levels and functions. In AILD patients, the MAIT cell population is predominantly the CD8^+^ subtype, which is significantly depleted in both peripheral blood and liver tissue. In addition, the expression of IL‐17 and GzmB increases, and these MAIT cells exhibit an exhausted state, marked by reduced IFN‐γ production and high expression of the activation and exhaustion markers CD38, HLA‐DR, and CTLA‐4 [23]. In AILD patients, MAIT cells mediate the activation of HSCs through IL‐17 expression, leading to the transformation of activated nonparenchymal hepatocytes into myofibroblasts that secrete ECM [22, 23, 301, 307]. Although MAIT cells from individuals with AILD are in an exhausted state, repeated IL‐12/IL‐18 stimulation induces liver‐enriched MAIT cells to enter an “exhaustion–activation” dual state, further leading to their expression of the profibrotic cytokine IL‐17A and the formation of the “IL‐12–MAIT–IL‐17–HSC” axis [23]. Furthermore, during chronic liver injury, owing to increased intestinal permeability, bacterial overgrowth, and dysbiosis, vitamin B2 metabolites produced via bacterial metabolism accumulate in hepatic myofibroblasts after MR1‐dependent engagement with MAIT cells. In the presence of MR1‐neutralizing antibodies, hepatic myofibroblast proliferation is significantly attenuated, indicating that MAIT cells promote disease progression by stimulating fibrotic cell proliferation in an MR1‐dependent manner [22].

In summary, MAIT cells are activated during chronic liver injury via both TCR–MR1‐dependent and TCR–MR1‐independent pathways, and produce profibrogenic factors that activate HSCs and promote liver fibrosis.

#### 5.3.3 Alcohol‐Related Liver Disease

The liver is a vital organ that is responsible for alcohol metabolism and for filtering toxins from the bloodstream; it is particularly susceptible to alcohol‐induced damage, as the metabolic byproducts of alcohol can cause severe liver injury, leading to ALD [308, 309, 310]. This includes the progression from simple hepatic steatosis to alcoholic hepatitis and alcohol‐related cirrhosis (ALC) [311]. Excessive alcohol consumption also increases susceptibility to viral infections [312, 313].

In ALD patients, increased expression of CD69, PD‐1, and HLA‐DR is observed in circulating MAIT cells, and MAIT cells are significantly depleted, which is most pronounced in individuals with alcoholic hepatitis and alcoholic cirrhosis [314, 315, 316]. Peripheral blood CD4^+^ and CD8^+^ MAIT cell counts are lower in patients with alcoholic cirrhosis and mixed cirrhosis than in healthy controls [317]. Research suggests that apoptosis or cell exhaustion may be key mechanisms underlying the reduced MAIT cell frequency: the serum levels of IL‐12 and IL‐23 are significantly elevated in highly activated MAIT cells from patients with cirrhosis, whereas the IL‐18 concentration markedly increases across all groups. Following the sustained activation of MAIT cells by IL‐12 and IL‐18, cotreatment with IL‐6 and IL‐8 secrete by MAIT cells significantly promotes a transform of hepatic myofibroblasts to a proinflammatory phenotype [22, 318]. In ALD patients, functional MAIT cells are highly activated and express elevated levels of IL‐17 and perforin [319]. IL‐17 modulates alcohol‐induced liver inflammation, fibrosis, bacterial dissemination, and tumorigenesis [320, 321, 322]. As a key inflammatory cytokine involved in ALD pathogenesis, reduced TNF‐α secretion may compromise the early control of pathogens in the liver [317]. Further studies revealed that during the ALC stage, peripheral blood innate immune cells, including MAIT cells, are further depleted, although the expression of CD69, PD‐1 and lymphocyte activating 3 (LAG‐3) on circulating MAIT cells is markedly increased, suggesting coexisting persistent activation and exhaustion, along with elevated IL‐17 expression, whereas IFN‐γ and TNF‐α expression are not significantly altered [316], further confirming a functional shift toward the proinflammatory IL‐17 axis.

In summary, the reduced number and functional reprogramming of MAIT cells in ALD patients not only reflect the dynamic evolution of disease activity and the immune microenvironment but also provide potential biomarkers and therapeutic targets for clinically assessing ALD severity, monitoring disease progression, and developing immune‐targeted interventions.

#### 5.3.4 Nonalcoholic Fatty Liver Disease

NAFLD is among the most common chronic liver diseases worldwide, with a global prevalence of 25.24% [323]. It encompasses a spectrum of conditions, including simple steatosis, nonalcoholic steatohepatitis (NASH), cirrhosis, and HCC. Immune responses play critical roles in NAFLD progression, as infiltrating innate and adaptive immune cells may accelerate and amplify the extent of liver injury [324]. Mucosa‐associated innate T cells participate in the pathogenesis of multiple inflammatory diseases, including NAFLD.

Studies have shown that compared with healthy controls, NAFLD patients present a reduced proportion of circulating MAIT cells but an increased frequency of MAIT cells in the liver [22, 325]. Functional alterations, including elevated expression of the markers CD69 and PD‐1, increased IL‐4 cytokine production, and increased expression of the chemokine receptors CXCR6 and CCR5, have also been observed; these findings indicate that MAIT cells migrate to the liver and adipose tissue as inflammatory cells in NAFLD patients [325, 326]. Using animal models, researchers further discovered that mice lacking MAIT cells exhibited severe fatty liver and changes in inflammation after being fed a choline‐deficient diet (MCD) that impaired hepatic β‐oxidation. Additionally, circulating MAIT cells are recruited to the liver tissue and subsequently activated in an MR1‐dependent manner. Activated MAIT cells induce monocyte and macrophage differentiation into the CD206^+^ anti‐inflammatory M2 phenotype by inducing their polarization and producing regulatory cytokines such as Th2 cytokines. These effects protect against NAFLD‐related inflammation, suggesting a novel therapeutic strategy for NAFLD [325].

### 5.4 Inflammatory Bowel Disease

IBD is a chronic, relapsing‐remissive gastrointestinal inflammatory disease. It is caused by a dysfunctional immune response, which induces aberrant immune attack of the gut microbiota. IBD can be classified into ulcerative colitis (UC) and Crohn's disease (CD) on the basis of distinct tissue characteristics and the distribution of inflammation. CD is characterized by intercalated transmural inflammation and excessive Th1 and Th17 responses, which may involve any part of the gastrointestinal tract, whereas UC is characterized by uniform mucosal inflammation and a Th2‐type‐like cytokine profile that is limited to the colon and rectal tissues [327]. Genetic predispositions, environmental factors, and microbiome composition contribute to the development of IBD; these factors can lead to microbial imbalance, impairment of the gut mucosal integrity, innate immunodeficiency, and adaptive immune dysregulation [327].

In IBD patients, the proportion of MAIT cells in the peripheral blood is decreased, whereas the proportion of MAIT cells in the intestinal mucosa is relatively increased, indicating that MAIT cells are recruited from the circulation to inflamed tissues [328, 329]. Although the number of MAIT cells in the inflamed intestinal mucosa of UC and CD patients is increased, this number is still lower than that in healthy mucosa, and the MAIT cells in IBD patients exhibit proapoptotic features [330]. In the microcirculation under inflammatory conditions, MAIT cells can respond to signals from proinflammatory cytokines, which indicates that in the absence of TCR signals, MAIT cells are indirectly activated by inflammatory factors [328]. Moreover, in IBD patients, the abundance of MAIT cells is correlated with disease activity. Activated MAIT cells in IBD patients accumulate in the inflamed mucosa and increase their secretion of TNF‐α and IL‐17, which may be involved in the immunopathogenesis of this disease [331]. In addition, MAIT cells play a pathogenic role in oxazolone‐induced colitis, and oral administration of an MR1 antagonist (such as isobutyl 6‐formyl pterin (i6‐FP)) decreases the levels of IFN‐γ and TNF‐α in MAIT cells but does not affect gut integrity [332]. These findings suggest that MAIT cells can increase disease severity in IBD patients.

### 5.5 Endocrine and Metabolic Diseases

#### 5.5.1 Type 1 Diabetes Mellitus

Type 1 diabetes mellitus (T1DM) is a chronic autoimmune disease characterized by the destruction of pancreatic β‐cells, which can lead to absolute or near‐absolute insulin deficiency [333]. The clinical symptoms of T1DM are heterogeneous and include polyuria, polydipsia, polyphagia, and ketosis. At initial diagnosis, approximately 25–50% of children have diabetic ketoacidosis (DKA), which is higher than the percentage of adults with DKA, which accounts for 6–21% [333]. The pathogenesis involves autoimmune β‐cell destruction and persistent hyperglycemia, leading to DKA [333, 334].

In individuals with T1DM, the decreased frequency of MAIT cells is accompanied by alterations in the expression of chemokines and tissue‐resident factors, which may be attributed to the redistribution of MAIT cells from the circulation to sites of inflammation within tissues [335]. Moreover, the functional alteration of MAIT cells can be induced by increasing the levels of inflammatory cytokine IL‐18 [336]. The development of diabetes in nonobese diabetic (NOD) mice is related to a reduction in gut mucosal integrity, decreased levels of the cytokines IL‐17 and IL‐22 in gut‐resident MAIT cells, and increased systemic inflammation [337], whereas increased levels of IL‐17A and IL‐22 can maintain intestinal homeostasis and tissue integrity [47, 337]. In children with T1DM, the proportion of MAIT cells in the blood is reduced, which may correspond to pancreatic migration [337]. In individuals who have had T1DM for a long time, MAIT cells exhibit an activated and exhausted phenotype characterized by the upregulation of CD25 and PD‐1 expression and decreased levels of the proinflammatory cytokines IL‐2, IFN‐γ, and TNF‐α [338]. Conversely, the accumulation of GzmB‐ and IFN‐γ‐secreting MAIT cells in the islets of Langerhans in the pancreas can destroy β‐cells. These findings suggest that MAIT cells play dual roles in T1DM [336].

#### 5.5.2 Type 2 Diabetes Mellitus

Type 2 diabetes mellitus (T2DM) is generally a chronic disease related to genetic and environmental factors. It is a multisystemic metabolic disorder characterized by high blood glucose levels, which results from progressive insulin dysfunction or tissue resistance to insulin [339]. The deterioration of islet β‐cell function is caused by various factors, such as glucotoxicity, oxidative stress, the induction of disallowed genes and cell death [339]. Young T2DM patients have microvascular complications (such as diabetic kidney disease, retinopathy, and peripheral neuropathy) and macrovascular complications (such as cardiovascular and renal complications). These complications adversely affect the quality of life of T2DM patients, which raises the possibility of future public health catastrophes [340].

In T2DM patients, the number of MAIT cells in the blood and adipose tissue is reduced, and these cells exhibit proinflammatory phenotypes, which are related to obesity and insulin resistance [336]. Circulating MAIT cells in T2DM patients significantly increase IL‐17 levels and are not influenced by specific TCR activation [341]. Bariatric surgery can restore the frequency of MAIT cells and decrease IL‐2 and GzmB production to some extent, suggesting that the abnormalities in MAIT cells are attenuated [341]. In addition, a large proportion of IL‐17‐secreting MAIT cells in T2DM patients with the CD27^−^ phenotype have increased mitochondrial mass, and their expansion is associated with dysfunction of the gut barrier, which induces the accumulation of *Bacteroides ovatus* (*B. ovatus*) in the peripheral blood [342]. The secretion of IL‐17 by MAIT cells can activate the JNK pathway and induce neutrophils to infiltrate pancreatic islets, resulting in impaired insulin signaling and β‐cell function [343]. Additionally, the secretion of TNF‐α and IFN‐γ by abnormal MAIT cells can induce insulin resistance in T2DM patients; the effect of TNF‐α is mediated by serine phosphorylation‐mediated impairment of insulin signaling, whereas the effect of IFN‐γ is mediated by downregulation of the insulin receptor [341, 344, 345]. These findings suggest that the secretion of TNF‐α, IFN‐γ, and IL‐17 by MAIT cells is involved in T2DM pathogenesis.

#### 5.5.3 Obesity

Obesity is a complex multifactorial disease characterized by the accumulation of excess body fat, which results from an imbalance between calorie consumption and expenditure. The pathogenesis of obesity involves multiple factors, including epigenetics, the social environment, the gut microbiome, and dietary quality. This condition is associated with a high risk of death and has become common worldwide [346].

In obese patients, the frequency of MAIT cells in the peripheral blood is decreased, but these cells are enriched in adipose tissue, where they predominantly secrete IL‐17 and cause insulin resistance [347]. MAIT cells in obese patients can be activated by cytokines such as IL‐12, IL‐18, and IL‐7 or in a TCR‐dependent manner. The recruitment of MAIT cells from peripheral blood to adipose tissue drives them into a chronically activated state and increases their susceptibility to death [341]. Moreover, the increased expression of IL‐17 in MAIT cells in obese patients is linked to an increase in mitochondrial ROS levels. This mitochondrial dysfunction in MAIT cells can be restored by the mitochondria‐targeted antioxidant MitoTEMPO, which in turn reduces IL‐17 production [348]. In addition, MAIT cells from adipose tissue can induce M1 macrophage polarization in a TCR‐dependent manner [35]. Thus, the administration of targeted inhibitory ligands or the reversal of mitochondrial dysregulation represents a potential therapeutic strategy to restore MAIT cell function and alleviate obesity in patients.

### 5.6 Respiratory System Diseases

#### 5.6.1 Asthma

Asthma is a common inflammatory airway disease characterized by cough, wheezing, shortness of breath, chest tightness and airway hyperreactivity in response to nonspecific respiratory irritants, all of which are driven by airway inflammation, and a variety of innate and adaptive immune cells are involved in the physiological and pathological processes of asthma [349, 350]. Several studies have demonstrated that the frequency of MAIT cells is reduced in peripheral blood, sputum, and bronchial biopsy samples from patients with asthma [351, 352, 353, 354, 355]. The expression of the activation marker CD69 and the exhaustion marker PD‐1 in MAIT cells from peripheral blood was significantly greater in neutrophilic asthma (NA) patients than in healthy controls, suggesting that the reduced frequency of MAIT cells in NA patients may be due to their apoptosis and depletion induced by overactivation [354]. The frequency of MAIT cells is correlated with disease severity [351, 354]. Asthma is a common disease in children. A recent study revealed that a higher frequency of MAIT cells in peripheral blood at 1 year of age was associated with a decreased risk of asthma at 7 years of age. Furthermore, an increased MAIT cell frequency is positively correlated with IFN‐γ production by activated CD4^+^ T cells, indicating that MAIT cells preferentially produce Th1 cytokines that suppress Th2 responses and contribute to asthma protection [356].

However, another study demonstrated that although there was no significant difference in the frequency of circulating MAIT cells between children with and without asthma exacerbations, children with exacerbated asthma exhibited a greater proportion of MAIT17 cells, which was positively correlated with the number of severe exacerbations and negatively correlated with the Asthma Control Test score [357]. Moreover, the frequency of MAIT17 cells in the BALF was significantly greater than that in the blood in children with severe asthma (SA); additionally, patients in the high‐MAIT17 group experienced more severe exacerbations than those in the low‐MAIT17 group did [358]. Furthermore, the levels of IL‐17A and TNF‐α secreted by MAIT cells are increased in NA patients, whereas IFN‐γ secretion is decreased, indicating that MAIT cells in NA patients are biased toward the Th17 subtype [354]. In asthma patients, IL‐7 levels are elevated in peripheral blood and induced sputum, whereas IL‐7R expression on circulating MAIT cells is reduced, suggesting that MAIT cells may be activated by the cytokine IL‐7 to secrete the proinflammatory cytokines IL‐17A and TNF‐α [354]. Increased levels of IL‐17A and TNF‐α act synergistically to induce IL‐6 and IL‐8 secretion from respiratory epithelial cells, leading to neutrophil recruitment to inflamed areas and worsening asthma symptoms [354]. In addition, CD69^+^ MAIT cells are positively correlated with airflow limitation in patients with asthma [359]. After 1 year of treatment with mepolizumab, the frequency of circulating CD69^+^ MAIT cells in patients with SA significantly decreased compared with that at baseline, and most patients demonstrated improved asthma control [360]. However, this reduction was not significantly associated with treatment efficacy, indicating a concomitant phenomenon rather than a direct cause of symptom improvement [360]. In summary, these findings indicate that MAIT17 cells are associated with asthma symptoms and that MAIT cells may play a role in asthma pathogenesis.

#### 5.6.2 Chronic Obstructive Pulmonary Disease

COPD is caused by exposure to inhaled noxious particles such as tobacco smoke and air pollutants combined with genetic, developmental, and social factors and is characterized by persistent airflow obstruction and respiratory symptoms [361]. Like those in asthma patients, circulating MAIT cell levels, especially CD8^+^ MAIT cell subsets, are significantly reduced in COPD patients, whereas the frequency of MAIT cells in the sputum is not significantly reduced in patients with stable COPD [362, 363]. Compared with patients with mild COPD, patients with moderate to severe COPD presented significantly lower circulating MAIT cell levels. Additionally, elevated serum C‐reactive protein levels and a reduced the forced expiratory volume in 1 s/forced vital capacity ratio are associated with decreased MAIT cell frequency in COPD patients [362]. These findings indicate that a reduced MAIT cell frequency reflects inflammatory activity and disease severity in COPD patients. Moreover, MAIT cell counts in peripheral blood and the expression of CD38 and LAG‐3 on MAIT cells are independently correlated with the risk of hospitalization, suggesting that COPD progression might be related to increased MAIT cell activation and the recruitment of MAIT cells to the airways [364]. Compared with those from healthy individuals, MAIT cells from COPD patients produced more IL‐17 and less IFN‐γ and presented increased expression of the activation marker HLA‐DR, but MR1 expression in lung tissues between the COPD group and the control group was not significantly different [365]. These findings suggest that MAIT cells are more likely to be activated through the cytokine pathway rather than the TCR pathway and are polarized toward Th17 cells, thereby playing a potential role in COPD immunopathology [365]. In contrast, another study revealed that the mRNA expression levels of MR1 in the blood of COPD patients are significantly greater than those in healthy controls [363]. Furthermore, when human pulmonary macrophages were cocultured with nontypeable *H. influenzae*, the most common bacterium that colonizes the airways of COPD patients, MR1 expression levels on the surface of human pulmonary macrophages increased, which promoted MAIT cell activation and induced their secretion of IFN‐γ [116]. This process can be blocked by MR1‐neutralizing antibodies, suggesting that MAIT cells are activated in a TCR–MR1‐dependent manner rather than in a TCR‐independent manner [116, 366]. Moreover, studies have indicated that multiple components of cigarette smoke (CS) can competitively bind MR1, thereby inhibiting the activation of MAIT cells [366, 367]. Long‐term exposure to CS alters the phenotype and function of MAIT cells, potentially increasing susceptibility to infection and disease exacerbation [367].

### 5.7 Skin Diseases

#### 5.7.1 Dermatitis Herpetiformis

Dermatitis herpetiformis (DH) is an inflammatory skin disease that affects primarily gluten‐sensitive adults and is characterized by intense itch and blisters, and symmetrical rashes on the elbows, knees, and buttocks and is a common extraintestinal manifestation of CeD [368]. Research has shown that the frequency of MAIT cells is similar in normal skin and healthy PBMCs and that skin MAIT cells highly express the cutaneous lymphocyte antigen and the tissue residency marker CD103, suggesting the presence of resident MAIT cells in normal human skin [369]. Moreover, the percentage of MAIT cells in psoriasis and alopecia areata lesions is similar to that in normal skin but significantly elevated in DH lesions [369]. MAIT cells can secrete TNF‐α, IFN‐γ, and IL‐17, all of which are associated with DH; therefore, researchers have speculated that MAIT cells may be involved in DH pathogenesis through these cytokines [369].

#### 5.7.2 Atopic Dermatitis

Atopic dermatitis (AD) is the most common chronic inflammatory skin disease and affects more than 200 million people worldwide [370]. Its primary characteristics include eczematous eruptions accompanied by intense itch [370]. Compared with that in healthy controls, the proportion of MAIT cells in peripheral blood was significantly lower in AD patients; additionally, the expression of CD69 in MAIT cells was significantly lower, whereas CD38 expression was significantly greater. Moreover, the numbers of polyfunctional GzmB and TNF‐α‐producing MAIT cells with upregulation of CD38 expression and IL‐22 production were significantly greater, suggesting that MAIT cells might play a role in the pathogenesis of this disease [371]. In an MC903‐induced murine model of AD, skin bacterial metabolites presented by MR1 are recognized by TCR on MAIT cells, triggering MR1‐dependent MAIT cell activation, which subsequently drive eosinophil activation and the recruitment of IL‐4‐ and IL‐13‐producing cells, exacerbating itch and barrier disruption to promote disease progression [372].

### 5.8 Cancer

In addition to conventional T cells, nonconventional T cells participate in tumor immunity. When they span the innate–adaptive continuum, these cells rapidly respond to nonpeptide antigens via conserved TCRs or by cytokine activation, thereby coordinating multiple facets of the immune response, mediating both antitumor and protumor activities [373]. MAIT cells have been identified in individuals with various cancers, including solid tumors such as colorectal cancer [374], cervical cancer [375], lung cancer [376], HCC [377], and renal cell carcinoma [378] and malignant hematologic diseases such as chronic lymphocytic leukemia (CLL) and multiple myeloma (MM). Cancer affects the frequency, phenotype, and function of MAIT cells, which play both protective and pathogenic roles, depending on the cancer type and microenvironment [1].

#### 5.8.1 Solid Tumors

In solid cancers, the formation of the local tumor microenvironment (TME) is correlated with disease progression and can reduce the efficacy of immune checkpoint blockade and adoptive cell therapies. The TME is composed primarily of tumor‐associated macrophages (TAMs), myeloid‐derived suppressor cells (MDSCs), cancer‐associated fibroblasts, and innate T‐like cells; among these, MAIT cells exhibit moderate tumor cell killing capacity and possess both antitumor and anti‐TAM/MDSC functions [379]. In most tumor patients, circulating MAIT cell counts are reduced, while tumor sites show substantial MAIT cell accumulation, as observed in colorectal cancer patients [374]. With respect to solid cancers, MAIT cells can be engineered to target and modify the TME. Additionally, MAIT cells can undergo chimeric antigen receptors (CAR)‐T‐cell engineering to increase antitumor activity [380].

MAIT cells exhibit both antitumor and protumor effects. Angiogenesis plays a critical physiological role in tumors, regulating tumor growth and metastasis through the formation of new blood vessels. These new vessels supply oxygen and nutrients to tumor cells while removing metabolic byproducts, thereby counteracting hypoxia and cell death [381]. Cytokines and chemokines secreted by MAIT cells, such as IL‐17, CXCL8 (IL‐8), and GM‐CSF, play roles in inducing angiogenesis and tumor cell proliferation, thereby triggering metastasis [377, 381], as noted in colorectal cancer [382] and HCC [377]. Furthermore, MAIT cells may exert protumor effects by suppressing antitumor cell functions, such as accelerating tumor metastasis by inhibiting the antitumor activity of NK cells [383]. With respect to antitumor function, studies using mouse tumor models have revealed that the number of MAIT cells is negatively correlated with solid tumor weight and that the presence of CD8^+^ T cells is strongly associated with reduced tumor burden [384]. Following the selective activation of MAIT cells using 5‐OP‐RU combined with the TLR9 agonist cytosine–guanine (CpG), liver MAIT cell expansion increased up to 70‐fold, which was accompanied by an increase in the frequency of CD8^+^ T cells in liver tumors, exhibiting a distinct effector memory (CD44^+^CD62L^−^) phenotype; these cells produced antitumor molecules such as granzyme, perforin, and IFN‐γ and secreted the protumor factor IL‐17A [384]. Compared with controls, 5‐OP‐RU+CpG combination treatment reduced IL‐17A expression while enabling MAIT cells to produce greater amounts of antitumor effector molecules and lyse tumor cells [384]. Furthermore, 5‐OP‐RU stimulation induces substantial IFN‐γ and TNF production, increasing antitumor effects through IFN‐γ‐dependent NK cell activation [385]. Benjamin Ruf et al. demonstrated that MAIT cell‐mediated antitumor immunity is independent of MR1 expression on tumor cells [384]. Conversely, MR1‐dependent MAIT cell activation has been demonstrated in other mouse cancer models [383], promoting tumorigenesis and metastasis [386].

In summary, the precise role of MAIT cells in solid tumors remains unclear, as they may exhibit both antitumor and protumor effects. However, analysis of their antitumor activity in animal models and humans suggests that MAIT cells still hold promise for cancer immunotherapy.

#### 5.8.2 Hematologic Malignancies

Since MAIT cell‐specific effector molecule targets are expressed in multiple hematologic malignancies, MAIT cells may also represent attractive targets for cellular therapy or immunotherapy for patients with hematologic malignancies [387]. The studies describe MAIT cells in patients with a hematologic malignancy focused on MM and reveal significantly reduced MAIT cell frequencies, particularly the CD8^+^, CD8^−^CD4^−^ subset cells [388, 389], with a dysfunctional Th1 response and reduced IFN‐γ and TNF‐α production [390]. The precise role of MAIT cells in MM remains unclear; however, studies have demonstrated that MAIT cells mediate the killing of myeloma cell lines, suggesting the potential for MAIT cell‐based immunotherapy [388, 391]. A study of acute myeloid leukemia (AML) patients revealed significantly reduced MAIT cell levels in newly diagnosed patients, with the degree of reduction correlated with high‐risk cytogenetic profile and isocitrate dehydrogenase 1 and 2 (IDH1/2) mutations. These findings suggest that reduced MAIT cell numbers or loss of MAIT cell function may be associated with AML disease progression [392]. MR1 expression has been detected in MM cells and leukemia cells, suggesting that MAIT cells may participate in the development and progression of malignant hematologic tumors via MR1 molecules on monocytes or B‐cell‐related tumor cells [387, 388]. Wallace et al. reported MAIT cell deficiency in CLL patients [393], although the authors did not establish a causal relationship between MAIT cell deficiency and CLL pathogenesis or suggest potential associated mechanisms. Thus, MAIT cells play a significant role in the development and suppression of hematologic malignancies. No definitive conclusions exist regarding whether MAIT cells exert antitumor or tumor‐promoting effects across different tumors or microenvironments. The potential role of MAIT cells in hematologic malignancies warrants further investigation.

The intratumoral microbiota also plays an important role in the TME and is associated with tumor progression [28]. Tumor‐resident microbes secrete metabolites such as 5‐OP‐RU to activate MAIT cells, which mediate antitumor activity toward target cells. However, the roles of microbial metabolites in the context of cancer are complex and can promote the divergent outcomes of MAIT cells by supporting tumor progression and immune regulation [28]. Moreover, the response of MAIT cells to tumor microbial metabolites can remodel the TME and increase overall immune reactivity [28].

Overall, MAIT cells play multiple roles in various immune‐related diseases. Owing to the high structural flexibility of MR1, MAIT cells can recognize a large variety of chemically diverse ligands with diverse chemical structures. In addition, cytokines from an inflammatory environment can upregulate MR1 expression and provide nonspecific signal to prime MAIT cell activation, especially in individuals with autoimmune diseases [21]. The alteration of gut homeostasis disrupts the balance among different bacteria and the levels of ligands they produce in the context of immune‐related diseases. This dysbiosis constitutes another possible source of TCR‐dependent activated MAIT cells. In addition, MAIT cells can respond to inflammatory cytokines without TCR ligation [24]. Although etiological heterogeneity exists among immune‐related diseases, the frequency of MAIT cells decreases in the peripheral blood but increases in inflamed tissues, which is attributed to the expression of adhesion molecules, integrins, and chemokines. These molecules can trigger the migration of MAIT cells to inflamed tissues. Additionally, in individuals with many autoimmune and liver diseases, such as cirrhosis, PSC, NAFLD, NASH, and SLE, persistently activated MAIT cells express increased levels of activation and exhaustion markers (such as CD38, PD‐1, and TIM‐3) and exhibit an exhausted phenotype in peripheral blood and target tissues. This state is driven by long‐term IL‐12 and IL‐18 stimulation [22, 23, 212, 325, 394]. Moreover, dysregulated MAIT cells primarily exhibit a Th17 phenotype and secrete IL‐17 and IL‐22, which play pathogenic roles in various inflammatory diseases. The levels of IL‐17 and IL‐22 are positively associated with disease severity. In addition, the cytotoxic factors secreted by MAIT cells can impair target tissues and cells, such as β‐cells, in patients with diabetes, which suggests another pathogenic role of MAIT cells in immune‐related diseases. Furthermore, in experimental autoimmune encephalomyelitis, models overrepresentation of T cells expressing the Vα 19i TCR transgene (Vα19i T cells) decrease the production of the cytokines IFN‐γ, IL‐17, and TNF but increase the production of IL‐10, which suggests that Vα19i T cells can mediate the suppression of autoimmune diseases [249]. In NAFLD patients, increased IL‐4 but decreased IFN‐γ and TNF‐α levels in MAIT cells have been detected. These shifts in cytokine levels can induce anti‐inflammatory macrophage polarization and protect against the inflammatory response [325]. These findings suggest that the roles of MAIT cells can be either pathogenic or protective and that the functions of MAIT cells are orchestrated by distinct activation modes.

## 6 Applications of MAIT Cells for Disease Treatment

As described above, there is considerable evidence indicating the multiple roles of MAIT cells in various pathogenic and immune‐related diseases. Therefore, the development of tools such as MR1‐loaded ligands or MAIT‐cell‐based adjuvants for MAIT cell activation and expansion has facilitated researchers to explore their functions in preclinical animal experiments. Moreover, the microbiota can be utilized via a “bug–drug” strategy to improve the functions of MAIT cells. Furthermore, the application of CAR‐modified MAIT cells has made it possible to strengthen the effects of MAIT cells and broaden their response to microbial infections. The following sections describe the modification strategies and applications of MAIT cells in the treatment of various diseases.

### 6.1 MAIT Cell Vaccines

MAIT cells have demonstrated unique potential in immune responses, particularly in the fields of vaccine design and immune enhancement. The abundance, their intrinsic effector memory phenotype, and mucosal residency of MAIT cells make them excellent candidates for the development of vaccines [7]. MAIT cell‐activating ligands can be used as adjuvants to synergize with therapeutic vaccines, thereby amplifying MAIT cell responses during microbial infections. To date, the development of MAIT cell‐targeting vaccines has focused primarily on three strategies [395]. First, a MAIT cell agonist (5‐OP‐RU) is used as a mucosal adjuvant that is coadministered with microbial antigens to activate MAIT cells, which can secrete proinflammatory cytokines and coordinate with DCs through CD40–CD40L interactions, subsequently promoting Tfh cell differentiation and inducing robust humoral immunity. Treatment with 5‐OP‐RU can promote MAIT cell expansion and decrease the bacterial loads of *Mtb* in an IL‐17A‐dependent manner [67]. Second, viral vectors and mRNA vaccines trigger an innate immune response and increase the levels of IL‐12, IL‐18, and Type I IFN. These cytokines can activate MAIT cells in a cytokine‐dependent manner. Enhanced antigen‐specific CD8^+^ T‐cell responses have been observed in response to nonreplicating adenoviral vector (ChAdOx) administration, whereas enhanced B‐cell responses have been observed after the administration of replication‐competent adenoviral vectors. Third, 5‐OP‐RU can be used in combination with replicating viruses to activate MAIT cells, thereby promoting their polarization toward the MAIT1 phenotype. This leads to increases in the levels of IFN‐γ or TNF‐α and antigen‐specific CD8^+^ T‐cell responses. In addition, other vaccines, such as BCG, can activate MAIT cells via both TCR‐dependent and cytokine‐dependent pathways, and vaccine adjuvants that synergize with inactivated or protein vaccines can also trigger MAIT cells via TLR pathways [203].

In terms of vaccine design, MAIT cells can be used as cellular adjuvants to promote the activation of local immune cells, which can increase humoral and cellular immune responses. During SARS‐CoV‐2 or influenza virus infection, the coadministration of a MAIT cell agonist with viral antigens mediate DC activation and prime Tfh cells to induce protective humoral immunity [162]. As a vaccine adjuvant, 5‐OP‐RU can coordinate with viral vaccines to expand MAIT cells in multiple tissues and reprogram them to the MAIT1 phenotype to induce virus‐specific CD8^+^ T‐cell responses, which can improve the antiviral protection of vaccines [161]. In response to chimpanzee Ad Ox1 (ChAdOx1) immunization, the IFN‐α, IL‐18 and TNF secreted by plasmacytoid DCs and monocytes can activate MAIT cells. These activated MAIT cells can increase vaccine‐induced CD8^+^ T‐cell immunity in humans [396]. The cytokine profile depends on vaccination with the Ad backbone, which can bypass TCR‐mediated activation and provoke a strong proinflammatory response [397]. These findings offer valuable insights for exploring activation strategies in vaccine development.

### 6.2 Microbial “Bug–Drug” Strategies for MAIT Cells

Owing to the dysbiosis of the microbiota during the progression of several diseases, microbe‐based strategies are effective in improving the effects of MAIT cells in related diseases. Several approaches, such as probiotic supplementation and genetic engineering of bacteria, can increase the production of riboflavin metabolites and promote MAIT cell activation and improve their ability to fight diseases [28]. For instance, in an appendix organoid model derived from patients with acute pediatric appendicitis, *E. coli*‐infected appendix organoids synthesized and presented the bacterial MR1 ligand 5‐OP‐RU, triggering MAIT cell‐mediated cytotoxicity to kill the organoid cells [398]. *E. coli* is a strong stimulator that presents riboflavin metabolites to MAIT cells in a TCR‐dependent manner, and the activated MAIT cells secrete IFN‐γ, TNF, and GzmB to drive fine‐tuned functional responses [399]. Thus, the integration of microbe‐based strategies and MAIT cell therapy provides a promising reference for the development of novel therapeutics [28].

### 6.3 MAIT Cell Therapy

CAR‐T‐cell therapy is an autologous immunotherapy that involves the collection of a single unit of the patient's cells, the genetic modification of their T cells in vitro to express synthetic receptors that bind specific tumor antigens and the reinfusion of the modified T cells back into the patient [400]. Engineering immune cells with CARs represents a promising strategy for disease immunotherapy. In addition to classical cytotoxic CD8^+^ T cells, innate cell types have been utilized to generate CAR‐T or CAR‐NK cells [401, 402]. These include well‐established candidates such as γδT cells [403], macrophages [404], and nonclassical cells like MAIT cells [405], which have also been tested as CAR‐modified cells programmed to become effective CAR–MAIT cells. CAR–MAIT cell therapy has emerged as a novel cancer immunotherapy approach. Studies have demonstrated that the expression of anti‐CD19 and anti‐Her2 CARs on activated primary human MAIT cells and CD8^+^ T cells, followed by their in vitro expansion, allows effectively targeting of B‐cell lymphoma and breast cancer cells. CAR–MAIT cells are highly toxic to their target cells, at a level comparable to that of CD8^+^ CAR‐T cells. In the presence of the vitamin B2 metabolite 5‐A‐RU, MAIT cells killed MR1‐expressing breast cancer and B‐cell lymphoma cells in a dose‐dependent manner, demonstrating promising therapeutic efficacy [405]. Designing CAR molecules is the most critical step in CAR‐T‐cell therapy. The components typically included in CAR design are the CD8α signal peptide, a single‐chain antibody (scFv) targeting the antigen (conferring specific recognition ability), the CD8 hinge region, a transmembrane region, the 4‐1BB (CD137) intracellular domain, the CD3ζ domain (providing costimulatory signals), and tissue‐homing chemokine receptors. CAR–MAIT cells have shown dual cancer cell‐killing capabilities—broad‐spectrum targeting and precision targeting—by embedding scFv‐targeting microbial or tumor antigens into the 4‐1BB and ‐CD3ζ signaling scaffold while preserving the intrinsic MR1 antigen presentation function of the MAIT cell [405, 406].

MAIT cells combine the innate mucosal and barrier tissue‐directed migration, rapid effector function, and sensitivity to microbial metabolites inherent in MAIT cells [11, 214]. Compared with CAR‐T cells, CAR–MAIT cells may exhibit lower immunogenicity, increased tissue infiltration capacity. Consequently, they hold unique therapeutic promise for treating diseases [405]. CAR–MAIT cells have demonstrated breakthrough therapeutic potential in multidrug‐resistant infections and immune‐mediated liver diseases, and their utility extends further to include noninfectious conditions such as cancer, autoimmune disorders, and chronic inflammatory diseases [228, 407]. In infectious diseases, CAR–MAIT cells theoretically serve as an immune defense while precisely targeting pathogen‐infected cells or pathogen‐associated antigens, which promotes the release of proinflammatory factors and toxic molecules to combat infected cells [1]. Furthermore, leveraging the highly coupled characteristics of MAIT cells with the gut microbiota and IL‐17 axis in inflammation is promising for designing CAR–MAIT molecules that target inflammation in specific tissues. This approach enables their directed entry into lesion sites in typical “gut–joint/gut–skin axis” immune diseases. These MAIT cells can then exert local immunomodulatory effects, eradicate pathogenic microorganisms, and eliminate abnormal APCs [206, 286, 298, 408, 409]. In the future, the key issues that need to be resolved for CAR–MAIT cells in infectious and immune diseases include how to balance CAR signaling with endogenous TCR/MR1 signaling to avoid inflammation or cell exhaustion, how to optimize the IL‐17/IFN‐γ signature through a costimulatory domain design for tailored disease contexts, and how to sustain durable yet controllable effector function in microenvironments characterized by chronic inflammation. By integrating the feasibility and safety advantages demonstrated with MAIT cell‐based CAR engineering strategies in tumor immunity [405, 406, 410], CAR–MAIT cells hold promise for translational applications in controlling drug‐resistant microbial infections and IL‐17‐driven immune disorders, and will become a significant future direction in the development of cell therapies.

### 6.4 Preclinical Experiments in Animal Models

MAIT cells have shown numerous applications and various effects in mouse and nonhuman primate models (Table 1). There are several ways to generate *MR1^−/−^* mice on a C57BL/6 genetic background. First, standard gene targeting techniques can be used to delete exons 2 and 3 (encoding the α1 and α2 domains) of the mouse *MR1* gene, followed by intercrossing the heterozygous mice carrying this deletion to obtain *MR1^−/−^* mice [10]. Second, *Vα19iCα^−/−^MR1^−/−^* mice can be crossed with C57BL/6 mice to obtain the F1 generation, which is subsequently inbred to generate *MR1^−/−^* mice [145]. Third, the CRISPR/Cas9 system can be used to delete exons 1–4 and the promoter region of the *MR1* gene in the C57BL/6 background to generate *MR1^−/−^* mice [411]. In a previous study, researchers amplified fragments containing the Vα19–Jα33 MAIT cell junction from the genomic DNA of enriched CD4^−^ CD8^−^ (DN) TCRαβ^+^ cells to construct a *Vα19i* expression plasmid and then injected the purified linear *Vα19i* transgene fragment into C57BL/6 one‐cell embryos to generate *Vα19i* transgenic founder mice [412]. These mice were subsequently crossed with TCR‐α‐deficient (*TCRα^−/−^*) mice on a C57BL/6 background *(Cα^−/−^)* and *MR1^−/−^* mice to generate *Vα19i* TCRα chain‐deficient (*Cα^−/−^*) and MR1‐sufficient (*MR1^+/+^*) and *Vα19iCα^−/−^MR1^−/−^* mice. Compared with wild‐type C57BL/6 mice, *Vα19iCα^−/−^MR1^+/+^* mice presented a significantly greater frequency of MAIT cells, whereas *Vα19iCα^−/−^MR1^−/−^* mice lacked MAIT cells [412]. *Vα19i* transgenic mice have been used to investigate the role of MAIT cells in *Francisella tularensis* (*F. tularensis*) [413, 414] and mycobacterial [415, 416] infections. Additionally, a study demonstrates that the frequency of MAIT cells in clean wild‐derived inbred CAST/EiJ mice is 20 times greater than that in C57BL/6J mice [417]. By crossing CAST/EiJ mice expressing the MAIT^hi^ phenotype with C57BL/6 (B6) mice for more than 10 generations, researchers generated B6‐MAIT^CAST^ mice, which exhibit high numbers of MAIT cells [417]. Researchers who used CAST/EiJ mice have revealed that MAIT cells confer protection against *Mtb* infection [68]. Moreover, B6‐MAIT^CAST^ mice have been used to investigate the functions of MAIT cells in nephritis [418], liver fibrosis [419], skin injury [54], and cancer [385, 420, 421]. A previous study used CRISPR/Cas9 to delete the *Traj33* gene in C57BL/6 blastocysts, generating chimeric founder mice; these founders were subsequently backcrossed and intercrossed with wild‐type C57BL/6 mice to obtain Traj33‐deficient (*Traj33^−/−^*) mice, which presented a severe reduction in the number of MAIT cells [422]. *Traj33^−/−^* mice have been utilized to investigate the role of MAIT cells in Vogt‒Koyanagi‒Harada (VKH) disease [423] and colitis [424]. Recently, researchers successfully reprogrammed MAIT cells isolated from the lung tissue of male C57BL/6 mice into iPSCs [425]. These iPSCs were injected into 8‐cell‐stage embryos of ICR mice to generate male chimeras, which were then crossed with C57BL/6NJcl females to screen and breed mice carrying the rearranged TCRα or TCRβ locus characteristic of MAIT cells, designated Vα19 and Vβ8 mice, respectively [426]. These strains exhibit significantly increased numbers of MAIT cells and have been used to explore the roles of MAIT cells in cancer and allergic airway inflammation [426, 427].

**TABLE 1:** Application and effects of MAIT cells in mouse and nonhuman primate models.

	Animal model	Application(s)	Effects	Reference(s)
Mice	MR1*^−^* ^/^ *^−^*	Exploring the developmental mechanisms of MAIT cells; serving as a negative control for functional studies	MR1 influences the development of MAIT cells and modulates their anti‐infective and antitumor functions.	[10, 145, 411]
	*Vα19iCα^−/−^MR1^+/+^ Vα19iCα^−/−^MR1^−/−^*	Investigating the role of MAIT cells in infections caused by *F. tularensis*	MAIT cells exhibit activity against *F. tularensis*. The development of optimal MAIT cells depends on MR1 expression, and MR1 deficiency reduces the proinflammatory response of MAIT cells.	[412, 413, 414]
	CAST/EiJ	Investigating the effects of MAIT cells on *Mtb*	MAIT cells exert a protective effect against *Mtb* infection.	[68, 417]
	B6‐MAIT^CAST^	Investigating the role of MAIT cells in nephritis, liver fibrosis, skin injury, and cancer	MAIT cells contribute to the suppression of inflammatory myeloid cells in immune‐mediated nephropathy. MAIT cells can promote liver fibrosis, tissue repair and antitumor activity.	[54, 385, 418, 419, 420, 421]
	*Traj33^−/−^*	Investigating the role of MAIT cells in VKH and colitis	MAIT cells have a protective role in VKH but exacerbate colitis disease progression.	[423, 424]
	C57BL/6NJcl	Investigating the antitumor activity of MAIT cells	MAIT cells were isolated from the lung tissue of C57BL/6NJcl mice, reprogrammed, and redifferentiated into m‐reMAIT cells, which proved their antitumor activity.	[426]
	*Rag^−/−^*	Investigating the role of MAIT cells in *Alternaria*‐induced airway inflammation	MAIT cells restrict *Alternaria*‐induced airway inflammation and hyperresponsiveness.	[411]
	αβT cell‐deficient	Investigating the effects of MAIT cells on *V. cholerae* infection	MAIT cells can promote B‐cell differentiation and *V. cholerae*‐specific IgA antibody responses.	[89]
	MR1*^−^* ^/^ *^−^* P301S	Studying the role of MAIT cells in neuroinflammation	MAIT cell deficiency exacerbates neuroinflammation in P301S human tau transgenic mice.	[428]
Nonhuman primates	RMs	Investigating the role of MAIT cells in SIV infection	The increase in the numbers of TNF‐α‐ and IFN‐γ‐producing MAIT cells indirectly inhibits SIV by promoting B‐cell activation.	[71, 133, 429]
	MCMs	Investigating the effects of *Mtb* and SIV coinfection on MAIT cells	*Mtb*/SIV coinfection leads to MAIT‐cell dysfunction.	[59]
	PTM	Employing the PTM model to investigate MAIT cell dynamics during SIV infection	In a PTM model of SIV/simian HIV infection, MAIT cells proliferated and upregulated the α4β7 homing receptor, which suggests that the absence of MAIT cells in the intestinal mucosa may prevent their depletion during chronic infection.	[430]

*Abbreviations*: *Vα19iCα^−/−^*, *Vα19i* TCRα chain‐deficient; *MR1^−/−^*, MR1‐deficient; *Traj33^−/−^*, Traj33‐deficient; *Rag^−/−^*, RAG‐deficient; RMs, rhesus macaques; MCMs, Mauritian cynomolgus macaques; PTM, pigtail macaques; *F. tularensis*, *Francisella tularensis*; *Mtb*, *Mycobacterium tuberculosis*; VKH, Vogt‒Koyanagi‒Harada; *V. cholerae*, *Vibrio cholerae*; SIV, simian immunodeficiency virus.

MAIT cells have been used to fight diseases through adoptive transfer into various animal models. In a recent study, researchers performed a peritumoral injection of purified in vivo‐expanded MAIT cells into a mouse model of colon cancer and reported a significant reduction in tumor volume and weight; further analysis revealed that activated MAIT cells exerted antitumor effects by promoting the secretion of IFN‐γ, IL‐17, GM‐CSF, and eotaxin‐1, as well as by recruiting and activating eosinophils [431]. Furthermore, multiple researchers have investigated the roles of MAIT cells in various diseases and their potential as therapeutic targets by adoptively transferring them into RAG‐deficient (*Rag^−/−^*), *MR1^−/−^*, and αβT cell‐deficient mice. Ye et al. transferred MAIT cells into *Rag^−/−^* mice followed by *Alternaria* challenge; the influx of IL‐5 and IL‐13 produced by lung Group 2 innate lymphoid cells was markedly decreased, as was BAL eosinophilic infiltration, and airway hyperresponsiveness was restricted by the overexpression of the anti‐inflammatory molecule IL‐4‐induced gene 1 [411]. Zhang et al. reported that the adoptive transfer of MAIT cells into *MR1^−/−^* P301S mice could increase tau pathology, hippocampal atrophy, meningeal barrier integrity, and neuroinflammation [428]. Jensen et al. reported that the adoptive transfer of MAIT cells into αβT cell‐deficient mice following mucosal challenge with *V. cholerae* promoted B‐cell differentiation and enhanced *V. cholerae*‐specific IgA antibody responses [89].

The combination of 5‐OP‐RU and TLR agonist stimulation is an effective strategy to induce the activity of MAIT cells against pathogenic and nonpathogenic diseases. In mouse models of liver tumors, subcutaneous tumors, and lung metastases treated with a combination of 5‐OP‐RU and CpG, researchers reported a negative correlation between the number of tumor‐infiltrating MAIT cells and tumor weight. The activation of MAIT cells significantly inhibited tumor growth, indicating that the activation and expansion of MAIT cells can induce potent and broad antitumor responses [384]. Moreover, Zhao et al. demonstrated that systemic vaccination of mice with 5‐OP‐RU in combination with a CpG adjuvant led to the systemic activation and expansion of MAIT cells, driving their polarization toward the MAIT1 phenotype [432]. Upon challenge with *F. tularensis* systemic infection or *L. longbeachae* pulmonary infection, this vaccination regimen significantly reduced bacterial loads and improved survival, suggesting that vaccination can provide protection against both systemic and localized bacterial infections [432]. Sakai et al. reported that prophylactic vaccination with 5‐OP‐RU plus CpG in mice delayed the priming of *Mtb*‐specific CD4^+^ T cells and failed to confer protection against subsequent *Mtb* challenge; conversely, treatment with 5‐OP‐RU during chronic infection markedly increased the number of MAIT cells and reduced the bacterial load through an IL‐17A‐dependent mechanism [67]. IL‐23 also serves as a costimulatory signal to trigger the proliferation and activation of MAIT cells in vivo [433]. Wang et al. demonstrated that combining IL‐23 with a 5‐OP‐RU conjugate vaccine significantly increased MAIT cell activation and expansion, leading to better control of pulmonary *Legionella* infection in mice [433]. Furthermore, Li et al. developed a novel Mg/Al LDH‐5‐OP‐RU/mRNA nanovaccine [434]. After intranasal administration, this mRNA vaccine efficiently delivered antigens to the lung parenchyma and draining mediastinal lymph nodes, inducing DC maturation and MAIT cell activation, and leading to significant prevention of lung metastasis as well as exerting therapeutic effects against established metastatic tumor nodules in the lungs [434]. Rashu et al. reported that 5‐OP‐RU could serve as a nonclassical but potent and versatile vaccine adjuvant against respiratory viral pathogens [161]. 5‐OP‐RU synergizes with viral vaccines to markedly expand MAIT cells in multiple tissues by relying on the replicative capacity of the viral vaccine, TLR3 engagement, and IFN‐I receptor signaling, skewing them toward a proinflammatory MAIT1 phenotype, enhancing virus‐specific CD8^+^ T‐cell responses and improving protection against heterosubtypic influenza infection [161].

In contrast, when the MR1 ligands Ac‐6‐FP and i6‐FP are used in a mouse model, the activation of MAIT cells and the secretion of proinflammatory cytokines can be reduced, which also facilitates the fight against diseases. In a mouse model of T2DM with hindlimb and myocardial ischemia, researchers inhibited the activation of MAIT cells by injecting Ac‐6‐FP, which reduced the secretion of proinflammatory factors such as CC chemokine ligand 3 like‐1 (CCL3L1) and macrophage polarization toward a proinflammatory phenotype, thereby promoting angiogenesis and arteriogenesis, decreasing fibrosis, and alleviating impaired collateralization in diabetic mice [435]. Yan et al. confirmed that MAIT cells are activated by the recognition of MR1 expressed on tumor cells, subsequently suppressing the effector functions of NK cells in a partially IL‐17A‐dependent manner, thereby promoting tumor progression [383]. Anti‐MR1 therapy significantly increases the antitumor activity of NK cells and CD8^+^ T cells, suggesting that blocking MR1 or using inhibitory ligands such as Ac‐6‐FP to interfere with MAIT cell activation may represent a novel strategy for cancer immunotherapy [383]. Murayama et al. treated SLE mice with i6‐FP, which inhibited MAIT cell activation, the production of the proinflammatory cytokines TNF‐α, IL‐17, and IFN‐γ, and the expression of CD40L; this led to reductions in serum anti‐dsDNA antibody levels and glomerulonephritis severity and alleviated the course of lupus [215]. In addition, Yasutomi et al. demonstrated that i6‐FP treatment suppressed MAIT cell activation in mice with colitis and reduced the production of IFN‐γ and TNF‐α by MAIT, iNKT, γδ, and CD4^+^ T cells, thereby increasing the survival rate and alleviating weight loss and colon shortening in these mice [332].

In addition, nonhuman primate models such as Mauritian cynomolgus macaques (MCMs), pigtail macaques (PTMs), and rhesus macaques (RMs) are widely used to study the immunological effector functions of MAIT cells during infection and treatment [59, 69, 71, 133, 429, 430, 436, 437]. By utilizing the MCM animal model to study the effects of SIV and *Mtb* coinfection, researchers report that SIV does not affect the migration of MAIT cells to TB infection sites but impairs their functional capacity, which directly or indirectly disrupts the control of TB [59]. Moreover, the use of a PTM model reveals that MAIT cells exhibit unique low α4β7 intestinal homing characteristics without intestinal mucosal enrichment; moreover, the proliferation of MAIT cells is increased, the expression of the gut‐homing marker α4β7 is upregulated, and the rectal cell frequency is elevated following SIV infection. These findings suggest that the absence of MAIT cells in the intestinal mucosa may prevent their depletion during chronic infection, potentially offering new therapeutic strategies for disease management [430]. Furthermore, compared with that in uninfected RMs, the frequency of MAIT cells in the blood remains unchanged during acute SIV infection; after early ARV treatment, the frequency of MAIT cells in the blood increase significantly but remains extremely low in mesenteric lymph nodes [437]. Another study reveals that vaccinating RMs with an SIV vaccine, followed by SIV infection, significantly increases the frequency of MAIT cells in the blood and increases the number of TNF‐α‐ and IFN‐γ‐expressing MAIT cells [133]. MAIT cells can promote B‐cell activation, class switching, antibody secretion, and the generation of tissue‐like memory B cells in RMs infected with SIV postvaccination, thereby offering a novel target for vaccine design [133]. Although 5‐OP‐RU treatment of *Mtb*‐infected mice promotes MAIT cell expansion and increase their ability to control the bacterium, the same treatment strategy applied to *Mtb*‐infected macaques yields the opposite results [67, 69]. Although 5‐OP‐RU activates MAIT cells, it fails to induce their expansion; instead, PD‐1 expression on MAIT cells is markedly upregulated, which impairs their cytokine‐producing function [69]. In uninfected macaques, vaccinated with 5‐OP‐RU plus CpG, PD‐1 blockade only partially restores MAIT cell function but fails to induce MAIT cell expansion [69]. These findings indicate fundamental differences in MAIT cell responses to TCR stimulation between mouse and nonhuman primate models, suggesting potential barriers to the direct translation of immunotherapy strategies from mouse models to humans or primates and great challenges in the application of MAIT cell‐targeted therapies for TB.

### 6.5 Clinical Trials of MAIT Cells

In accordance with data from the Chinese Clinical Trial Registry (ChiCTR) (https://www.chictr.org.cn/index.html) and ClinicalTrials.gov (https://clinicaltrials.gov/) from the United States, a total of 11 clinical trials related to the study of MAIT cells have been conducted (Table 2). Ten of them were observational studies that focused on detecting the levels of MAIT cells and surface protein markers such as CD56, CD16, and chemokine receptors in patients with psoriasis, IBD, and spontaneous and recurrent miscarriage. Additionally, some studies further explored the correlations among the frequencies, phenotypes, biomarker expressions, and function of MAIT cells in the contexts of infection and noninfectious diseases, such as post‐transplant diseases, ischemic stroke, psoriasis, IBD, microbial infections, severe intestinal inflammatory events, antisynthetase syndrome, and interstitial lung disease. The remarkable features of these clinical trials are the use of MAIT cells as an observational index and analyses of MAIT cell association with disease severity. An interventional study (NCT03313102; Table 2) also used MAIT cells as an indicator of whether corticoids are effective at attenuating giant‐cell arteritis. To date, we have not found a specific clinical trial in which MAIT cells were used as an interventional variable to explore their direct effect on human diseases.

**TABLE 2:** Clinical trials registered on ChiCTR and ClinicalTrials.gov for studying MAIT cells.

Registration number	Registration title	Country	Primary sponsor	Objectives of the study	Study type	Study phase	Samples collected
ChiCTR2500095349	A prospective cohort study evaluating the correlation between MAIT cell functional marker expression in G‐CSF‐mobilized donor grafts and post‐transplant complications by using full‐spectrum flow cytometry	China	Peking University People's Hospital	Analyze the correlation between chemokine receptors and the expression of functional markers in different MAIT cell subsets from G‐CSF‐mobilized grafts and their association with posttransplant complications (such as GVHD, pulmonary infections and infectious diarrhea) by full‐spectrum flow cytometry; explore the role of diverse chemokine receptors across MAIT cell subsets; develop predictive models for intestinal GVHD and other posttransplant complications.	Observational	Exploratory research/pretrial	Blood
ChiCTR2400083709	The alterations of circulating mucosal‐associated invariant T cells and IL‐17 in acute ischemic stroke	China	Luoyang Central Hospital Affiliated to Zhengzhou University	Explore the relationships among circulating MAIT cells and IL‐17 and the onset and prognosis of stroke; find new potential therapeutic targets; provide new methods for predicting and improving the prognosis of ischemic stroke patients.	Observational	Retrospective	Blood
ChiCTR2400079484	The impact of ustekinumab treatment on peripheral blood MAIT cells in patients with psoriasis	China	Department of Dermatology, Tongji Hospital	Explore the changes in the level and functions of MAIT cells in the peripheral blood of psoriasis patients after treatment with ustekinumab.	Observational	Not indicated	Blood
NCT05598346	Significance of MAIT cells in inflammatory bowel disease	Egypt	Assiut University	Examine the level and function of MAIT cells in patients with inflammatory bowel disease for comparison with disease activity.	Observational	Unknown	Not provided
NCT04492098	Mucosal‐associated invariant T cells in cases of miscarriage	Egypt	Assiut University	Measure the percentage of MAIT cells and the expression of CD56 and CD16 on MAIT cells in cases of spontaneous and recurrent miscarriage.	Observational	Completed	Decidual tissues
NCT02403089	Ontogeny of MAIT cells in neonates and hematopoietic stem cell transplant recipients (NEOMAIT)	France	Assistance Publique–Hôpitaux de Paris	Primary objectives: first, quantify the rate and kinetics of MAIT cell expansion and maturation in neonates relative to gestational age and in pediatric HSCT recipients relative to donor stem cell source; second, to establish correlations between gut microbiota diversity or function and the maturation or function of MAIT cells in both neonates and HSCT recipients; and third, elucidate the associations among MAIT cells, gut microbiota composition, and the incidence of microbial infections and severe intestinal inflammatory events in term or preterm neonates and HSCT recipients.	Observational	Completed	Mononuclear cells from cord blood; rectal swabs; gastric aspirates
NCT06720818	Mucosal associated invariant T cell during viral pneumonia and acute respiratory distress syndrome (MAIT‐VECTORS)	France	University Hospital, Tours	Investigate the implications and potential for harnessing MAIT cells in patients with severe viral pneumonia and associated acute respiratory distress syndrome.	Observational	Recruiting participants	Blood and respiratory samples
NCT05984394	Evaluation of antigen‐specific T cells in patients with antisynthetase syndrome and interstitial lung disease (CYTILDASS)	France	Central Hospital, Nancy	Explore the correlation between BAL antigen‐specific Th1 and Th17 cells (including MAIT cells) at ILD diagnosis and clinical evolution after 6 months of treatment on the basis of initial ILD severity.	Observational	Recruiting participants	Bronchoalveolar fluid sample
NCT05054361	Crosstalk between mucosal‐associated invariant T (MAIT) cells and the gut microbiota and mucosa in the development of Type 1 diabetes in children (MAIT‐DT1)	France	Institut National de la Santé Et de la Recherche Médicale	Prospectively investigate changes in MAIT cells frequency, phenotype and function associated with the gut microbiota, gut integrity and the presence of Coxsackie virus B in pediatric patients with a high genetic risk of Type 1 diabetes and pediatric patients with recently diagnosed T1D for comparison with control cohorts.	Observational	Recruiting participants	Blood, stools, urines, and gut mucosa samples
NCT03313102	Study of T lymphocytes in patients with Horton disease (GAMAIT)	France	Centre Hospitalier Universitaire Dijon	Include many patients with giant‐cell arteritis to show that the persistence of a low number of circulating MAIT cells in patients treated with corticoids is a predictor of relapse. To carry out a pilot study to obtain preliminary data on these new markers.	Interventional	Unknown	Blood
NCT03886350	Implication of unconventional T lymphocytes in cystic fibrosis (UNLOCk)	France	University Hospital, Tours	Study the properties of unconventional T cells and their functions in cystic fibrosis patients using blood and sputum samples for correlation analysis with comprehensive clinical and microbiological data.	Observational	Completed	Blood samples and sputum samples

*Data sources*—Chinese Clinical Trial Registry (ChiCTR) (https://www.chictr.org.cn/index.html) and ClinicalTrials.gov (https://clinicaltrials.gov/).

*Abbreviations*: G‐CSF, granulocyte‐colony stimulating factor; GVHD, graft‐versus‐host disease; HSCT, hematopoietic stem cell transplantation; ILD, interstitial lung disease; T1D, Type 1 diabetes.

Overall, the use of MAIT cells has led to the development of several applications for treating diseases. MAIT cells can be activated and amplified upon stimulation with exogenous ligands or cytokines via vaccine immunization through the response to riboflavin metabolites from microbial stimulation or through adoptive transfer in mice. Animal models such as *Rag^−/−^*, *MR1^−/−^*, and *TCRα^−/−^* mice have been used to evaluate the efficacy of MAIT cells in combating various pathogenic or nonpathogenic diseases. In addition to having antitumor effects, CAR–MAIT cells have been shown to have a strong ability to control drug‐resistant microbial infections, although a balance between CAR signaling and endogenous TCR/MR1 signaling is needed to avoid excessive inflammation and cell exhaustion. To promote the application of MAIT cells, it is important to further translate these preclinical studies, whether theoretical or are applied in interventional clinical trials.

## 7 Conclusions and Prospects

Since they were discovered more than 30 years ago, MAIT cell applications have attracted increasing attention from scholars. The overall focus has been on their biology and functions in human health and various diseases, ranging from microbial infection to immune‐related diseases. The functions of MAIT cells in different contexts depend on their localization, circumstances, and activation mode. During homeostasis, commensal populations in hosts can secrete riboflavin to induce partial activation of MAIT cells in the gut, lung, and skin. After being activated, MAIT cells are involved in sustaining mucosal integrity and repairing tissue at sites of injury. In disease contexts, MAIT cells can be activated by different stimuli and play multiple roles [1, 24, 50]. Although the functions of MAIT cells in various infectious diseases are related to reducing the bacterial load, inhibiting viral replication and inducing apoptosis or autophagy in infected cells, the activation modes and functions of MAIT cells in the context of some fungal infections (such as *S. cerevisiae*, Mucorales, and *Pneumocystis*) are not clear. Moreover, the secretion of proinflammatory cytokines (IL‐17, IL‐23, and TNF‐α) by MAIT cells plays a pivotal role in exacerbating inflammation and the pathogenesis of many immune‐related diseases. MAIT cells can also trigger angiogenesis and cell proliferation in tumors. Some unknown mechanisms of MAIT cells, such as anti‐inflammatory and cytotoxic responses, may exert protective effects. Thus, deciphering the effects of the secreted cytokines is necessary for studying the mechanisms of MAIT cells in immune‐related diseases. To date, several concerns have not been fully addressed, and further exploration is needed.

First, it has been confirmed that the activities of MAIT cells are tailored by their tissue localization and the nature of the stimulus. Given that the frequency of MAIT cells varies among distinct tissues and disease context, how specific tissue environment influences the function of MAIT cells remains unknown. This may be related to the unclear level of MAIT cell activation; the lack of knowledge concerning the roles driving MAIT cell localization to specific tissues; the complexities of ligand stimulation; the upregulation of distinct transcription factors; and the secretion of a variety of cytokines, chemokines, and cytotoxic factors in different tissues or contexts. These speculations have not been verified until now. In addition, the association between MAIT cells and their resident tissue needs to be elucidated in the future.

Second, therapeutic strategies targeting exhausted MAIT cells require further investigation. Chronic and excessive microbial stimulation can induce the excessive exhaustion of MAIT cells, leading to functional impairment and reduced cell counts in the blood and tissues of hosts, thereby exacerbating disease progression. Therefore, modulating the exhausted state of MAIT cells and restoring their number and function represent critical therapeutic targets for disease remission, improvement, and treatment. This can be accomplished by targeting factors that induce MAIT apoptosis and exhaustion, thereby increasing the abundance and functionality of MAIT cells. For example, in ALD and AILD patients, sustained activation of IL‐12, IL‐18, and IL‐8 induces MAIT cell apoptosis [23, 317], and MAIT cells highly express the levels of PD‐1 and LAG‐3 [316]. These pathways can serve as targets for disease improvement, such as by the addition of neutralizing antibodies against exhaustion factors (anti‐PD1 and anti‐LAG3) to inhibit the exhaustion pathway. Moreover, CAR modification of MAIT cells has become a novel approach for treating multiple infections and immune‐related diseases. By incorporating scFvs, 4‐1BB–CD3ζ signaling, and tissue‐homing chemokine receptors, CAR–MAIT cells are endowed with both tissue‐directed migration properties and rapid effector function. However, the presence of endogenous TCR and MR1 signaling may increase CAR–MAIT cell activation and subsequently trigger their exhaustion, which is similar to recent indications that CD3/CD28 costimulation induces faster activation and subsequent fading than CAR in triple‐parameter reporter Jurkat T cells. The slower and prolonged activation of T cells by CAR signaling may promote their shift to an abnormal state [438, 439]. Recently, several CAR engineering approaches have focused on IL‐15 integration [440, 441]. Engineering LiPSC–GR1.1 with a mesothelin (MSLN)‐targeting CAR and IL‐15 promotes the robust redifferentiation of iPSCs into mature cells, activates iNK cells, which can kill tumor cells. Low expression levels of the cytokine inducible SH2‐containing protein (*CISH*), TGFB receptor 2 (*TGFBR2*), and basic leucine zipper ATF‐like transcription factor (*BATF*) of in MSLN‐targeting CAR–IL‐15 are detected. CAR–IL‐15 iNK cells can optimize their antitumor responses to reduce the risk of exhaustion [440]. The generation of allogeneic CD33‐directed CAR–NKT cells results in high yield, purity, and robustness. These cells are highly capable of homing to the BM and effectively targeting BM‐resident malignant blast cells. The incorporation of IL‐15 into this CAR construct can increase its persistence and long‐term effects, thereby mitigating the effects of exhaustion [441]. However, future studies are needed to explore targeted drugs and modification strategies for reversing the exhausted phenotype of MAIT cells.

Third, given that clinical trials related to MAIT cells have primarily been observational, the application of engineered MAIT cell technology, such as CAR–MAIT‐cell therapy, or the use of adjuvants should be accelerated. To date, a total of six CAR‐T‐cell products have been approved by the United States Food and Drug Administration and have focused mainly on hematologic malignancies [442]. Another statistical analysis revealed that a total of 1580 clinical trials related to CAR‐T‐cell therapy up to April 2024 were registered at ClinicalTrials.gov, the majority of which involved hematological malignancies (71.6%), whereas the fewest involved anti‐infective trials [443]. These reports suggest that the application of CAR‐engineered immune cells against infectious diseases is relatively lacking. Moreover, MAIT cell‐targeted vaccines can provide a promising strategy for inducing protective immunity. The bridge function of MAIT cells localized in mucosal tissues is important for vaccine efficacy [395]. MAIT cells can be triggered to prime antibody production and facilitate B‐cell or CD8^+^ T‐cell responses through three strategies: the coadministration of 5‐OP‐RU and microbial antigens, activation through the cytokines secreted by viral vectors and mRNA vaccines, and combined engagement of 5‐OP‐RU and replicating virus [203, 395]. However, viral vectors and mRNA vaccines can prime hosts to continuously secrete proinflammatory cytokines. This raises an important question: how can excessive MAIT cell activation be prevented from impairing antigen‐specific antibody production or MAIT cell exhaustion? The proper function of MAIT cells is essential for maintaining vaccine efficacy. The challenge in fine‐tuning the MAIT cell response is to precisely control TLRs, which are modulated by cytokines [161]. Solving this challenge represents a major step in the translation of MAIT cells into vaccine adjuvants.

Taken together, the data presented in this review not only provide a deeper understanding of the functional plasticity of MAIT cells in healthy and disease contexts but also broaden our knowledge of the potential applications of engineered MAIT cell technology and its limitations. With advances in techniques for fine‐tuning MAIT cell modifications, these cells can be precisely manipulated to balance metabolic and general health of individuals or restore homeostasis in various disease contexts.

## Author Contributions

Conceptualization: Yi‐Qun Kuang. Writing – original draft: Yu Zhao, Dan Liang, and Feng‐Mi Ou. Writing – review and editing: Yu Zhao, Dan Liang, Yang Gao, ‐Qing‐Qing Li, and Yi ‐ Qun Kuang. Visualization: Feng‐Mi Ou. All authors have read and approved the final manuscript.

## Funding

This work was supported by the National Natural Science Foundation of China (82460391 and 82360328), the “Xingdian Talents” Support Project of Yunnan Province (RLQB20220013), the Yunnan Health Training Project of High‐Level Talents (L‐2024027 and H‐2025027), the Joint Special Fund of the Department of Science and Technology of Yunnan Province–Kunming Medical University (202401AY070001‐237), the Research and Development Project for Key Technologies and Clinical Translation of Precision Diagnosis and Treatment of Common Major Kidney Diseases in Yunnan Province (202505AJ310005), the First‐Class Discipline Team of Kunming Medical University (2024XKTDYS09), and the Yunnan Key Laboratory of Organ Transplantation (202449CE340016).

## Conflicts of Interest

The authors declare no conflicts of interest.

## Ethics Statement

The authors have nothing to report.

## Data Availability

No new data were generated or analyzed in support of this review.
